# Race-Specific Spirometry Equations Do Not Improve Models of Dyspnea and Quantitative Chest CT Phenotypes

**DOI:** 10.1016/j.chest.2023.07.019

**Published:** 2023-07-26

**Authors:** Amy L. Non, Barbara Bailey, Surya P. Bhatt, Richard Casaburi, Elizabeth A. Regan, Angela Wang, Alfonso Limon, Chantal Rabay, Alejandro A. Diaz, Arianne K. Baldomero, Greg Kinney, Kendra A. Young, Ben Felts, Carol Hand, Douglas J. Conrad

**Affiliations:** a Department of Anthropology, University of California San Diego, La Jolla, CA; b Department of Medicine, University of California San Diego, La Jolla, CA; c Department of Mathematics and Statistics, San Diego State University, San Diego, CA; d Division of Pulmonary, Allergy, and Critical Care Medicine, University of Alabama at Birmingham, Birmingham, AL; e Rehabilitation Clinical Trials Center, Lundquist Institute for Biomedical Innovation at Harbor-UCLA Medical Center, Torrance, CA; f Division of Rheumatology and Department of Medicine, National Jewish Health, Denver, CO; g Oneirix Labs, Carlsbad, CA; h Division of Pulmonary and Critical Care Medicine, Brigham and Women’s Hospital, Harvard Medical School, Boston, MA; i Pulmonary, Allergy, Critical Care and Sleep Medicine Section, Minneapolis VA Health Care System, Minneapolis, MN; j Department of Epidemiology, Colorado School of Public Health, University of Colorado Anschutz Medical Campus, Aurora, CO; k Advanced Mathematical Computing, San Diego, CA

**Keywords:** ethnicity, pulmonary function test, race, reference equations, spirometry

## Abstract

**Background:**

Race-specific spirometry reference equations are used globally to interpret lung function for clinical, research, and occupational purposes, but inclusion of race is under scrutiny.

**Research Question:**

Does including self-identified race in spirometry reference equation formation improve the ability of predicted FEV_1_ values to explain quantitative chest CT abnormalities, dyspnea, or Global Initiative for Chronic Obstructive Lung Disease (GOLD) classification?

**Study Design and Methods:**

Using data from healthy adults who have never smoked in both the National Health and Nutrition Survey (2007-2012) and COPDGene study cohorts, race-neutral, race-free, and race-specific prediction equations were generated for FEV_1._ Using sensitivity/specificity, multivariable logistic regression, and random forest models, these equations were applied in a cross-sectional analysis to populations of individuals who currently smoke and individuals who formerly smoked to determine how they affected GOLD classification and the fit of models predicting quantitative chest CT phenotypes or dyspnea.

**Results:**

Race-specific equations showed no advantage relative to race-neutral or race-free equations in models of quantitative chest CT phenotypes or dyspnea. Race-neutral reference equations reclassified up to 19% of Black participants into more severe GOLD classes, while race-neutral/race-free equations may improve model fit for dyspnea symptoms relative to race-specific equations.

**Interpretation:**

Race-specific equations offered no advantage over race-neutral/race-free equations in three distinct explanatory models of dyspnea and chest CT scan abnormalities. Race-neutral/race-free reference equations may improve pulmonary disease diagnoses and treatment in populations highly vulnerable to lung disease.

## Graphical Abstract

**Figure:**
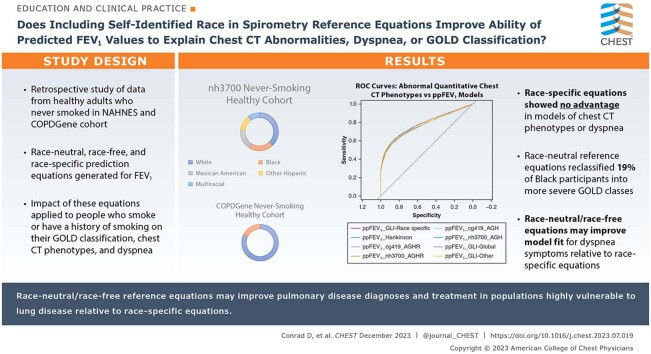

FOR EDITORIAL COMMENT, SEE PAGE 1348

**Take-home Points**

**Study Question:** Does including self-identified race in the formation of spirometry reference equations improve the ability of predicted FEV_1_ values to explain quantitative chest CT abnormalities, dyspnea, or Global Initiative for Chronic Obstructive Lung Disease classification?

**Results:** Race-neutral and race-free equations reclassified up to 19% of Black individuals who smoke to worse Global Initiative for Chronic Obstructive Lung Disease classes in the COPDGene smoking cohort, with the greatest effects seen in individuals with mild smoking-related disease. The generated percent predicted FEV_1_ values from race-neutral and race-free spirometry equations showed no significant improvement in model fit of dyspnea or quantitative chest CT phenotypes (emphysema, air trapping, airway wall thickness).

**Interpretation:** Race-neutral/free reference equations may improve pulmonary disease diagnoses and treatment in populations highly vulnerable to lung disease relative to race-specific equations.

Interpretation of spirometry results has traditionally relied upon reference equations to provide an estimate of “normal” lung function for an individual’s age, gender, height—and controversially—race/ethnicity. These equations are used for clinical, research, and occupational purposes to diagnose pulmonary disease, assess disease progression, and explain radiographic abnormalities, as well as determine disability and evaluate fitness for higher risk jobs, and thus have enormous clinical and financial importance. The inclusion of race in these equations is based on large cross-sectional, population-wide studies that consistently show lower measures of lung function for some racial/ethnic minority groups, specifically up to 10% to 15% lower FEV_1_ for Black individuals.^1^^,^^2^ However, the clinical value of race adjustments has increasingly been questioned.^3^, ^4^, ^5^, ^6^ Although recent studies found no prognostic benefit of race-specific equations compared with “race-neutral” equations in mortality or respiratory events,^7^, ^8^, ^9^, ^10^, ^11^ others continue to defend the use of race in prediction equations.^12^^,^^13^ Race-specific equations are recommended by the most recent US and European guidelines^14^ and are still used in clinical care and pulmonary research worldwide. However, applying race-specific equations may mask developmental or acquired lung damage among racial and ethnic minority groups^15^, ^16^, ^17^ and risks underdiagnosing damaged lungs in marginalized groups at high risk of respiratory disease,^18^, ^19^, ^20^, ^21^ thereby exacerbating racial health inequalities.

We examined how the percent predicted FEV_1_ (ppFEV_1_) values calculated from race-specific, race-neutral, and race-free reference equations differentially affect pulmonary phenotypes in two large cohorts of individuals who smoke. First, using a selected sample of healthy adults who have never smoked from both National Health and Nutrition Examination Survey (NHANES) (2007-2012) and COPDGene cohorts (e-Fig 1), we generated new race-free equations that entirely exclude race from model formation and race-specific prediction equations for FEV_1_ and FVC. Second, these equations were compared with the Global Lung Initiative (GLI) race-specific equations,^2^ the race-specific equations of Hankinson et al,^1^ and the race-neutral GLI-Other (uses a universal race-correction) and the GLI-Global (weights racial groups in the reference population) equations.^2^ Third, we applied these spirometry prediction equations and determined how they differentially: (1) affect the Global Initiative for Chronic Obstructive Lung Disease (GOLD) severity classification in both the NHANES and COPDGene smoking cohorts; and (2) model quantitative chest CT scan phenotypes and dyspnea in the COPDGene study participants. Our intent was to compare how the different reference equations model clinically important pulmonary phenotypes.

## Study Design and Methods

Details on formation and characterization of asymptomatic nonsmoking (nh3700 and cg419 cohorts) and smoking (nh785 and COPDGene Phase I) cohorts are presented in e-Appendix 1, e-Figures 1 to 3, and Table 1.

**Table 1:** Characteristics of the NHANES (N = 3,700) and COPDGene (N = 419) Healthy Cohorts Who Have Never Smoked by Race/Ethnicity

Characteristic	NHANES, All (n= 3,700)	NHANES, White (n = 1,420 [38.4%])	NHANES, Black (n = 762 [20.6%])	NHANES, Mexican American (n = 682 [18.4%])	NHANES, Other Hispanic (n = 473 [12.8%])	NHANES, Other/Mixed (n = 363 [9.8%])	COPDGene, All (n = 419)	COPDGene, White (n = 342 [81.6%])	COPDGene, Black (n = 77 [18.4%])
Age, y	51.0 (19.0)	52.0 (21.0)	52.0 (18.0)	49.0 (11.0)a	51.0 (20.0)	49.0 (17.0)a	59.1 (15.4)	61.4 (15.4)	55.1 (9.6)a
Female, n (%)	2,160 (58.4)	803 (56.5)	446 (58.5)	396 (58.1)	302 (63.8)	213 (58.7)	239 (57.0)	194 (56.7)	45 (58.4)
Height, cm	165.1 (14.4)	168.4 (15.1)	167.7 (13.4)	160.9 (12.7)a	160.3 (14.4)a	162.1 (12.9)a	168.6 (14.0)	168.7 (14.7)	168.0 (12.7)
Weight, kg	78.8 (25.1)	81.8 (26.1)	86.5 (27.0)a	75.4 (18.9)a	75.1 (22.7)a	66.1 (19.4)a	78.0 (23.1)	76.5 (23.9)	80.0 (19.2)
BMI, kg/m^2^	28.7 (7.4)	28.6 (7.6)	30.4 (8.2)a	29.2 (6.0)	28.6 (6.5)	24.9 (5.5)a	27.1 (6.0)	26.8 (5.9)	28.3 (5.11)
FEV_1_, L	2.83 (1.11)	3.07 (1.19)	2.57 (0.98)a	2.89 (1.01)a	2.70 (1.03)a	2.65 (0.94)a	2.83 (1.11)	2.92 (1.19)	2.62 (0.85)b
FVC, L	3.55 (1.41)	3.90 (1.51)	3.16 (1.16)a	3.58 (1.32)a	3.39 (1.25)a	3.30 (1.22)a	3.55 (1.38)	3.60 (1.45)	3.16 (1.20)a
FEV_1_/FVC	0.80 (0.07)	0.79 (0.07)	0.81 (0.07)a	0.81 (0.06)a	0.80 (0.06)a	0.80 (0.07)a	0.80 (0.08)	0.80 (0.07)	0.82 (0.07)a
ppFEV_1_ GLI^d^	99.0 (17.1)	99.8 (16.3)	98.0 (18.7)c	100.4 (16.2)	97.2 (16.4)c	96.8 (16.7)^c^	101.0 (18.7)	101.6 (18.6)	99.9 (16.7)
ppFEV_1_ GLI-Other^e^	102.61 (20.0)	107.1 (17.5)	90.5 (17.4)a	107.7 (17.4)	104.4 (17.6)c	96.8 (16.7)a	106.6 (20.3)	109.0 (20.0)	92.3 (16.2)a
qCT parameters									
Pi10							1.77 (0.42)	1.75 (0.41)	1.94 (0.44)c
Air trapping (n = 321)	…	…	…	…	…	…	7.18 (8.90)	7.20 (8.70)	6.80 (10.01)
Percent emphysema	…	…	…	…	…	…	0.84 (1.94)	0.88 (2.16)	0.62 (1.19)c
mMRC score (0-4)	…	…	…	…	…	…	0.00 (0.00)	0.00 (0.00)	0.00 (0.00)

Values of continuous variables are presented as medians (interquartile range). GLI = Global Lung Initiative; mMRC = modified Medical Research Council; NHANES = National Health and Nutrition Examination Survey; Pi10 = airway wall thickness estimate based on square root of wall area of a 10 mm lumen perimeter^22^; ppFEV_1_ = percent predicted FEV_1_; qCT = quantitative chest CT.

Indicates significant difference relative to White group at: ^a^*P* < .0001, ^b^*P* < .001, ^c^*P* < .05, according to the Kruskal-Wallis test for comparison of continuous variables between (non-Hispanic) Black and White racial/ethnic groups in COPDGene, and analysis of variance with Tukey ad hoc comparisons for continuous variables between each racial/ethnic group relative to White participants in the NHANES data.

d Guideline-based application of GLI race/ethnic-specific reference equations. The GLI equations for White/European individuals were used to estimate ppFEV_1_ for the NHANES Mexican American and Other Hispanic groups, following other studies (8). The GLI-predicted FEV_1_ values for the NHANES group “Other/Mixed Race” used the GLI-Other equations.

e The GLI-Other equation was used to generate race-neutral estimates of ppFEV_1_ for all racial/ethnic groups.

### Race-Specific and Race-Neutral Prediction Models

The predicted FEV_1_ and lower limit of normal (LLN) values for those that never smoked from the GLI equations were obtained using the GLI website (https://gli-calculator.ersnet.org/index.html, version 2.0, April 2023). Predicted and LLN FEV_1_ values by Hankinson et al^1^ (NHANES III) were calculated using published equations. For both never-smoking data sets (the NHANES data set of 3,700 healthy individuals [nh3700] and the COPDGene data set of 419 healthy individuals), multivariable linear quantile regression was used to generate predicted (median quantile) and LLN (fifth quantile) models and associated R1 values (Table 2). The R1 value is a measure of explained variability of the data in quantile regression and is used to compare models.^23^ Predictors in the race-specific equations included age (years), height (centimeters), gender (male/female), and self-identified race/ethnicity. Predictors in race-free equations included only age, height, and gender (e-Tables 1-3). A similar approach was used to generate models for predicted and LLN values for log (FVC). This approach generated four race-specific and four race-neutral/race-free models for the predicted log (FEV_1_) and log (FVC) from different source populations (e-Tables 1-3, Table 2, Table 3). Identity and probability density plots of the differences between the predicted race-specific, race-neutral, and race-free models were used to explore the effect of race in the models.

**Table 2:** Models of Predicted log (FEV_1_) Median and Fifth Percentile Quantile Regression Coefficients for NHANES (N = 3,700) and COPDGene (N = 419)

Model	β0 (Intercept)	β1 (Age in Years)	β2 (Female Gender)	β3 (Height in Centimeters)	β4 (Race)	R1
NHANES: log (FEV_1_)						
Median predicted: AGH	–0.30931	-0.00966	–0.16027	0.01177	...	0.485
Median predicted: AGHR (White)	–0.42116	–0.00922	–0.14935	0.01253	Reference	0.547
AGHR (African American)					–0.17048	
AGHR (Mexican American)					–0.00454	
AGHR (Hispanic Other)					–0.0219	
AGHR (other/mixed)					–0.09983	
5th percentile: AGH	–0.37667	–0.01069	–0.16976	0.01091	...	...
5th percentile: AGHR (White)	–0.55486	–0.01094	–0.15568	0.01258	Reference	...
African American					–0.19925	
Mexican American					–0.00657	
Hispanic Other					–0.03935	
Other/Mixed					–0.09986	
COPDGene: log (FEV_1_)						
Median predicted: AGH	–0.40813	–0.00958	–0.17036	0.01262	...	0.476
Median predicted: AGHR (Black)	–0.37885	–0.01077	–0.15455	0.01294	–0.16483	0.531
5th percentile: AGH	–0.04169	–0.01084	–0.17910	0.00946	...	...
5th percentile: AGHR (Black)	–0.14623	–0.01251	–0.15592	0.01097	–0.17822	...

The equation used to model the predicted log (FEV_1_) is as follows: predicted (or lower limit of normal) FEV_1_ (L)= e^(β0 + Age ∗ β1 + Gender code term ∗ β2 + Height ∗ β3 + Race code term ∗ β4)^. In both cohorts, race is coded as Black (1) relative to NHW (0) as the reference group. In data from the NHANES data set of 3,700 healthy individuals, race is modeled with NHW (0) as the reference group**,** and other racial/ethnic groups were coded as 1 if present and multiplied by the corresponding race coefficient (β4). In both cohorts, gender code term for male subjects is 0 and 1 for female subjects. The log of FEV_1_ (post-bronchodilator values were used when available for NHANES data and always for COPDGene data) in liters was used because it optimized the explained variability compared with modeling raw FEV_1_ values. The racial terminology used is consistent with that of the published cohorts. AGH = models including age, gender, and height only; AGHR = models including age, gender, height, and race/ethnicity; NHANES = National Health and Nutrition Examination Survey.

**Table 3:** Summaries of Source Data, Covariates, and Nomenclature for Predicted FEV_1_ Models

Predicted FEV_1_ Model	Type	Race	Source Population	Source Sample Size by Race/Ethnicity	Source Age Range	Covariates	Reference
GLI race-specific	QR-Linear	Specific	International	Total: n = 74,187 White: n = 57,395 Black: n = 3,545 NE Asian: n = 4,992 SE Asian: n = 8,225	3-95 y	AGHR	3
Hankinson	OLS-Linear	Specific	NHANES-1999	Total n = 7,429 White: n = 2,281 Black: n = 2,508 Mexican American: n = 2,639	8-80 y	AGHR	2
cg419_AGHR	QR-Linear	Specific	COPDGene never-smoking cohort	Total: n = 419 White: n = 342 Black: n = 77	45-82 y	AGHR	Table 2
nh3700_AGHR	QR-Linear	Specific	NHANES 2007-2012 healthy individuals who formerly smoked	Total: n = 3,700 White: n = 1,420 Black: n = 762 Mexican American: n = 682 Other Hispanic: n = 473 Other: n = 363	35-79 y	AGHR	Table 2
GLI-Other	GAMLSS	Neutral	International	n = 74,187	3-95 y	AGH^a^	3
GLI-Global	GAMLSS	Neutral	International	n = 74,185	3-95 y	AGH^b^	24
cg419_AGH	QR-Linear	Free	COPDGene healthy never-smoking	n = 419	45-82 y	AGH	Table 2
nh3700_AGH	QR-Linear	Free	NHANES 2007-2012 healthy never-smoking cohort	n = 3,700	35-79 y	AGH	Table 2

Details about models and source populations used to develop each of the race-specific, race-neutral, and race-free models used in this study. GLI-Other and GLI-Global equations are race-neutral, but are not race-free, as they averaged race/ethnicity estimates across four major racial/ethnic groups. AGH = age, gender, and height; AGHR = age, gender, height, and race; cg419 = COPDGene data set of 419 healthy individuals; GAMLSS = General Additive Models for Location Scale and Shape; GLI = Global Lung Initiative; NE = northeast; nh3700 = National Health and Nutrition Examination Survey data set of 3,700 healthy individuals; NHANES = National Health and Nutrition Examination Survey; OLS = ordinary least squares regression; QR = quantitative regression; SE = southeast.

a GLI-Other was calculated by taking “its mean and CoV adjustments the corresponding adjustments for the four main ethnic groups, averaged over group and sex”.^3^

b For GLI-Global, an inverse probability weight was applied for each of the four racial groups included in the data set.

### GOLD Classification Changes

Each individual in both smoking cohorts was assigned a GOLD spirometry class (GOLD 1-4), the preserved ratio impaired spirometry class,^25^ or GOLD 0 (ie, FEV_1_/FVC ratio > 0.7 and ppFEV_1_ ≥ 80%) using the different race-specific and race-neutral equations. The percentage of individuals who changed GOLD class from the GLI standard (race-specific) was calculated in the total data set and within each racial group for both NHANES participants who formerly smoked and COPDGene participants who currently smoke.

### Modeling Pulmonary Phenotypes

#### Sensitivity/Specificity Modeling

Measured FEV_1_ values were classified as above or below the LLN to assess the sensitivity and specificity of each ppFEV_1_ reference equation to model abnormal chest CT scan phenotypes in COPDGene phase I participants. Chest CT scan phenotypes were defined as abnormal if: (1) the percent emphysema was > 5%; (2) the percent air trapping was > 15%; or (3) the airway wall thickness estimate based on square root of wall area of a 10 mm lumen perimeter was > 2.5.^26^ The sensitivity, specificity, negative predictive value, positive predictive value, and the area under the curve (AUC) were calculated for each model in the overall population and within each race. A parallel approach assessed the ability of the LLN of each model to predict a modified Medical Research Council dyspnea score (mMRC) > 1.

#### Logistic Regression Models of Abnormal Chest CT Scan Phenotypes and Dyspnea

Because complex demographic factors (smoking status and history, gender, FEV_1_/FVC ratio, and scanner type) and the ppFEV_1_ influence quantitative chest CT scan metrics,^27^^,^^28^ multivariable logistic regression models were generated of abnormal chest CT scan phenotypes using these covariates and the ppFEV_1_ values derived from each of the race-neutral and race-specific equations (e-Tables 4A, 4B). Models were compared by using Akaike and Bayesian information criteria. A parallel approach was used to model dyspnea and included covariates of FEV_1_/FVC ratio, pack-year smoking history, age, weight, height, 6-min walking distance, total lung capacity from the CT scan,^27^ and the pre-/post-bronchodilator difference of FEV_1_ and ppFEV_1_ values calculated from the different race-neutral and race-specific equations (e-Tables 5A, 5B).

#### Random Forest Models

The random forest algorithm was used to compare models of the abnormal chest CT phenotypes and dyspnea using the same covariates as the logistic regression models (e-Tables 4C, 4D, e-Tables 5C, 5D). The classification error rates were compared to assess model performance using the different race-specific and race-neutral ppFEV_1_ values.

#### Software

All analyses used features in the base R (version 4.0.5) program (R Foundation for Statistical Computing). Additional packages included rspiro (v.2), quantreg (v5.55), pROC (v1.18.0), randomForest (v4.6-14), and rfPermute (v2.1.81).

## Results

The nh3700 never-smoking, healthy cohort consisted of 38% White, 21% Black, 18% Mexican-American, 13% other Hispanic, and 10% mixed racial or “other” race individuals (Table 1). Relative to White participants, Black participants had similar height but a higher median weight. All other groups had lower median weight and height than White participants. All racial/ethnic groups had a lower median FEV_1_ and FVC, and a higher median FEV_1_/FVC ratio, compared with White participants. White and Mexican-American individuals had higher GLI race-specific ppFEV_1_ values than the other ethnic groups.

The COPDGene never-smoking, healthy cohort consisted of 18% Black and 82% White participants. Relative to White participants, Black participants were younger, had lower FEV_1_ and FVC values, and had higher FEV_1_/FVC ratios, consistent with other studies.^1^^,^^8^ Black participants had significantly higher airway wall thickness estimate based on the square root of wall area of a 10 mm lumen perimeter and lower percent emphysema relative to White participants (e-Fig 2, Table 1). Compared with the nh3700 never-smoking cohort, the COPDGene healthy participants who had never smoked were older and less racially diverse, but they were otherwise similar in demographic, anthropometric, and spirometry assessments (Table 1). Compared with the COPDGene smoking cohort, the nh785 cohort of individuals who formerly smoked was younger, with higher BMI and higher FEV_1_ and FVC values, suggesting less severe smoking-related disease (e-Fig 3).

### Comparison of Models Among Those Who Never Smoked

There was a high correlation between predicted values generated using all race-specific equations for FEV_1_ and for LLN (e-Fig 4). Probability density plots of the differences between the FEV_1_ values generated using the different race-specific equations show minor differences in the predicted FEV_1_ values between Black and White populations (e-Fig 5). These high correlations show the validity of the generated healthy data sets and the modeling approach. In contrast, race-neutral equations generated higher predicted FEV_1_ and LLN values than race-specific equations for the Black participants but unchanged or minimally shifted to lower values in the White participants of both healthy, never-smoking cohorts (e-Figs 6, 7). Race-free models generated even higher predicted FEV_1_ and LLN values than those of GLI-Global (e-Fig 8). There were no differences between any racial groups in the density plots of predicted FEV_1_ when using race-neutral equations. Including race in the FEV_1_ prediction equations improved model fit as measured by slightly higher R1 values in both never-smoking, healthy populations, but it can also obscure measured differences in FEV_1_ between White and Black individuals in both never-smoking, healthy populations (e-Fig 9, e-Table 1, Table 2). To a lesser extent, this effect was also seen between White and other/mixed race populations in the nh3700 population (e-Fig 10). Using alternative anthropometric measurements instead of height did not improve model fit or mitigate racial differences (e-Fig 11).

#### Race-Specific vs Race-Neutral Equations in Individuals Who Smoke

**Figure 1:**
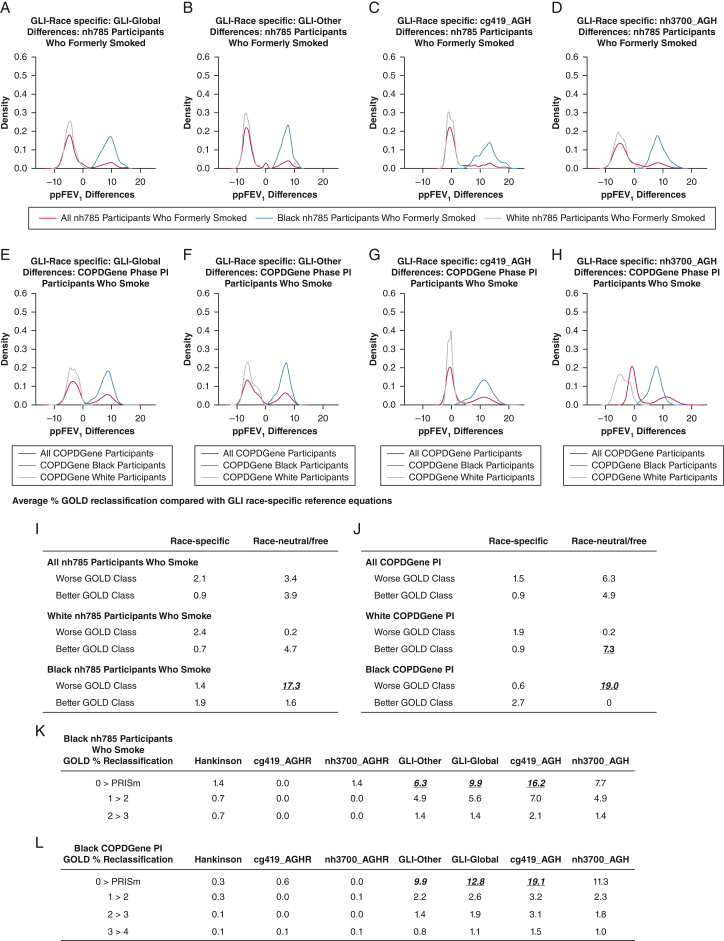
A-L, Effects of race-specific vs race-neutral/free equations on predicted FEV_1_ values and GOLD reclassification. Probability density of the differences in ppFEV_1_ values in NHANES and COPDGene participants who smoked between GLI race-specific and GLI-Global (A, E), GLI Race-specific and GLI-Other (B, F); GLI race-specific and cg419_AGH (C, G), and GLI race-specific and nh3700_AGH (D, H). Differences in ppFEV_1_ were calculated by subtracting each ppFEV_1_ estimate derived from race-neutral or race-free equations from the estimate derived from the GLI standard (race-specific) equation. Red lines = all COPDGene PI participants; blue lines = Black phase I participants; gray lines = White phase I participants. Average GOLD reclassification rates of the three race-specific (Hankinson, cg419AGHR, and nh3700AGHR) and three race-neutral/race-free (GLI-Other, cg419_AGH, and nh3700_AGH) prediction equations were subtracted from the standard GLI equation in the combined Black/White cohort, Black, and White participants in the nh785 smoker cohort (I) and the COPDGene PI cohort (J), reported as the average percent reclassified. GOLD 0 class is defined as FEV_1_/FVC ratio > 0.7 and ppFEV_1_ > 80%. PRISm class is defined as FEV_1_/FVC ratio < 0.7 and ppFEV_1_ < 80%. GOLD reclassification rates (percent reclassified of total Black population) are shown in Black nh785 participants who formerly smoked (K) and Black COPDGene particiants who smoke (L) from the GLI race-specific equations by GOLD class transitions (rows) and models (columns). AGH = age, gender, and height; AGHR = age, gender, height, and race; cg419 = COPDGene dataset of 419 healthy individuals; COPDGene PI = COPDGene Study Phase I; GLI = Global Lung Initiative; GOLD = Global Initiative for Chronic Obstructive Lung Disease; nh3700 = National Health and Nutrition Examination Survey data set of 3,700 healthy individuals; NHANES = National Health and Nutrition Examination Survey; ppFEV_1_ = percent predicted FEV_1_; PRISm = preserved ratio impaired spirometry.

The ppFEV_1_ value generated from the new race-specific equations were within 3% of the estimates generated using GLI race-specific equations for the total COPDGene smoking population (e-Fig 12). In contrast, the differences between GLI race-specific and race-neutral/race-free ppFEV_1_ values in both smoking cohorts created bimodal curves with the Black population shifted positively, corresponding to a lower ppFEV_1_ by an average of approximately 7% to 11% (Figs 1A-1H); the curves in the White population shifted negatively, which would result in slightly higher ppFEV_1_ values. Similar to values seen in the healthy never-smoking cohort, the race-free equations generated lower ppFEV_1_ values than race-neutral equations in COPDGene participants who smoked (e-Fig 13).

### Model Effects on GOLD Classification

To identify the differential effects of the ppFEV_1_ equations on GOLD classification, we identified the number of individuals who were reclassified using the newly developed race-specific equations compared with the GLI race-specific equations. In the two smoking cohorts, the GOLD reclassification rate on average changed 1% to 3% when the calculated ppFEV_1_ values using new race-specific reference equations were subtracted from those calculated by using GLI race-specific reference equations (Figs 1I, 1J). When stratified according to race, across all four race-neutral and race-free equations, an average of 17.3% of Black participants in the nh785 smoking cohort were reclassified to worse GOLD classes. In the more severely diseased COPDGene cohort (e-Fig 3), 19.0% of the Black participants were reclassified to worse GOLD classes using race-neutral/race-free equations. Most of these GOLD reclassifications were transitions from GOLD 0 (ie, ppFEV_1_ > 80 and FEV_1_/FVC ratio > 0.7) to the preserved ratio impaired spirometry class (Figs 1K, 1L).

### Quantitative Chest CT Scan and Dyspnea

**Figure 2:**
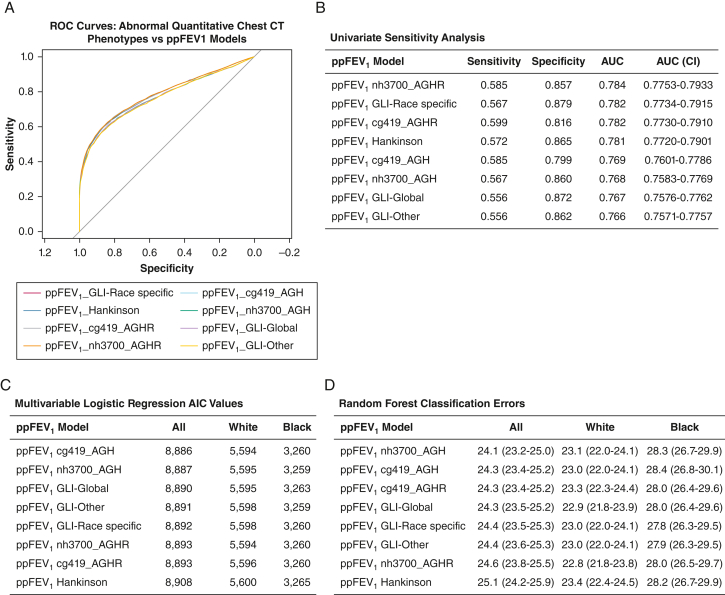
A, ROC curves of any abnormal quantitative chest CT phenotypes: (1) percent emphysema > 5%; (2) percent air trapping > 15%; and (3) airway wall thickness if the airway wall thickness estimate based on square root of wall area of a 10 mm lumen perimeter. B, Sensitivity, specificity, AUC and AUC confidence intervals (CI) of the ROC curve of the ppFEV_1_ to predict any abnormal chest CT scan phenotype in the COPDGene phase I cohort. The sensitivity and specificity analyses used the lower limit of normal or the fifth percentile for each of the models. C, AIC from the multivariable logistic regression models of any abnormal quantitative CT phenotype with the following covariates: FEV_1_/FVC ratio, smoking history (pack-years), scanner maker, smoking status, gender, and ppFEV_1_. The AIC value generated from the models using the different race-specific and race-neutral equations for the ppFEV_1_ are listed for the total cohort and also for the Black and White participants individually. D, Supervised random forest models of the abnormal chest CT scan phenotypes were generated using the same covariates as the logistic regression models. The classification error rates of the models using the different race-specific and race-neutral equations for the ppFEV_1_ are listed for the total cohort and also for the Black and White individuals individually. The randomForest (v4.6-14) and rfPermute (v2.1.81) packages were used to obtain the classification error rates, mean decrease in accuracy, and P values. The default settings were used with ntree and nrep set to 500. AGH = age, gender, and height; AGHR = age, gender, height, and race; AUC = area under the curve; AIC = Akaike information criterion; cg419 = COPDGene data set of 419 healthy individuals; GLI = Global Lung Initiative; nh3700 = National Health and Nutrition Examination Survey data set of 3,700 healthy individuals; ppFEV_1_ = percent predicted FEV_1_; ROC = receiver-operating characteristic.

Three distinct models were used to compare the utility of the ppFEV_1_ and respective LLN values from race-specific and race-neutral equations to model abnormal chest CT scan phenotypes and increased dyspnea (ie, mMRC score > 1). The sensitivity and specificity of each ppFEV_1_ reference equation to model any abnormal chest CT scan findings were within 9%. Furthermore, the equations generated overlapping receiver-operating characteristic curves and thus similar AUC values. This univariate analysis showed no clinically relevant advantage of the race-specific equations over race-neutral equations to model chest CT scan abnormalities (e-Fig 14, Figs 2A, 2B).

The multivariable logistic regression models of abnormal chest CT scan phenotypes showed similar Akaike and Bayesian information criteria values using the race-specific and race-neutral equations in the total COPDGene phase I cohort as well as in the Black and White cohorts individually (e-Tables 4A, 4B, Fig 2C).

Finally, a supervised random forest approach was used to model the presence of any abnormal chest CT phenotype in the total COPDGene phase I population. Classification error rates differed minimally across equations in the total, Black, and White populations, and they also differed minimally between each of the quantitative chest CT scan phenotypes. There were no instances in which the race-specific equations offered any significant decrease in classification errors over the race-neutral or race-free equations (e-Tables 4C, 4D, Fig 2D).

**Figure 3:**
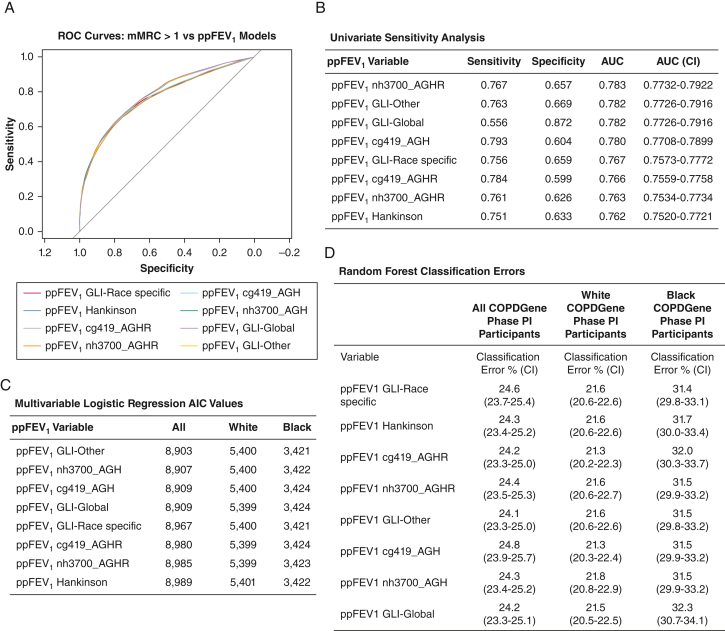
A, ROC curves of dyspnea in COPDGene PI participants who smoked as predicted by ppFEV_1_. Each colored line corresponds to a ROC curve using ppFEV_1_ values derived from the listed race-specific and race-neutral model equations. B, Sensitivity, specificity, and AUC of the ROC curve of the ppFEV_1_ to predict dyspnea (ie, mMRC score > 1). C, AIC from the multivariable logistic regression models of dyspnea (ie, mMRC score > 1), with the following covariates: FEV_1_/FVC ratio, smoking history (pack-y), scanner maker, smoking status, gender, and the ppFEV_1_. D, Classification error rates are presented from supervised random forest models of dyspnea (ie, mMRC score > 1), which were generated using the same covariates as the logistic regression models, for the total COPDGene PI cohort, and for White and Black participants individually. AGH = age, gender, and height; AGHR = age, gender, height, and race; AUC = area under the curve; AIC = Akaike information criterion; COPDGene PI = COPDGene phase I; cg419 = COPDGene data set of 419 healthy individuals; GLI = Global Lung Initiative; mMRC = modified Medical Research Council; nh3700 = National Health and Nutrition Examination Survey data set of 3,700 healthy individuals; ppFEV_1_ = percent predicted FEV_1_; ROC = receiver-operating characteristic.

Findings from models assessing dyspnea revealed trends similar to the models of quantitative chest CT phenotypes. Specifically, the sensitivity/specificity, receiver-operating characteristic, and AUC values were nearly identical using race-specific and race-neutral/race-free equations and their respective LLNs (e-Fig 15, Figs 3A, 3B). In univariate analysis, the sensitivity and AUC values of the ppFEV_1_ models were higher in White relative to Black COPDGene participants, regardless of whether race-specific or race-neutral/race-free ppFEV_1_ models were used (e-Fig 15). The Akaike information criterion values from the multivariable logistic regression models of dyspnea were lower using the race-neutral or race-free vs race-specific ppFEV_1_ values in the total COPDGene phase I population; this finding suggests improved model fit, but the clinical significance of this difference is unclear (e-Tables 5A, 5B, Fig 3C). Finally, classification error rates in the supervised random forest models were within 1% of each other using race-neutral/race-free vs race-specific equations, indicating no advantage in predicting dyspnea using race-specific equations in the total COPDGene phase I population or in analyses stratified according to race (e-Tables 5C, 5D, Fig 3D). The similarity in model fit when using GLI-Global vs race-free equations is unsurprising considering the very similar predicted FEV_1_ distributions in identity and density plots of healthy populations (e-Figs 8, 16).

## Discussion

This study investigates the role of race-specific, race-neutral, and race-free ppFEV_1_ reference equations in evaluating disease severity and pulmonary phenotypes. Using three different modeling approaches, we show that race-specific equations offer no advantage relative to either race-neutral or race-free equations in modeling quantitative chest CT scan phenotypes or dyspnea in two independent smoking cohorts. Specifically, we found that, compared with race-specific equations, race-neutral/race-free equations reclassified up to 19.0% of Black participants into more severe GOLD classes, and they may improve models of dyspnea. Use of race-neutral/race-free equations may result in additional pulmonary disease diagnoses, as well as more aggressive treatment in populations highly vulnerable to lung disease.

Our findings are in line with other recent studies that found no prognostic benefit of race-specific over race-neutral spirometry reference equations.^7^, ^8^, ^9^, ^10^, ^11^ Specifically, two studies found that the use of a race-neutral equation better predicted survival than race-specific equations using the NHANES III and 2007 to 2012 data sets, consistent with earlier findings.^9^^,^^29^ Similarly, Baugh et al^7^ found that race-neutral equations improved prediction of respiratory symptom burden in individuals who smoke at risk for COPD. Another prospective study identified no benefits of race-specific equations for predicting symptom burden or mortality from chronic lower respiratory disease in a mixed-race population.^8^ Finally, Liu et al^10^ noted that race-specific equations relative to race-neutral equations may be underdiagnosing emphysema among Black participants.

Others justify the ongoing use of race/ethnicity to determine spirometry reference values, citing risk of discriminatory hiring practices, denial of health insurance,^13^ or risk of overdiagnosis of lung disease in Black individuals and underdiagnosis in White individuals.^12^ We believe that these risks are of lesser potential harm than the risk of underdiagnosing the group with the highest rate of respiratory illness, which already receives poorer pulmonary care.^30^^,^^31^ There is an urgent moral obligation to reduce risk to the group most historically harmed and also most at risk for lung disease. In terms of discriminatory hiring, our primary concern is for the health of the individuals who may be at risk of exacerbating illness if granted hazardous jobs. All patients, regardless of race or baseline lung function, should be evaluated prior to being granted a hazardous job, and followed up longitudinally with risk counseling. Clinicians should also not rely on a single predicted spirometry value for risk assessment and instead gather longitudinal measures and contextualize clinical findings with lifetime exposure data, particularly for patients with borderline values.

Our data, along with the growing base of similar literature,^7^, ^8^, ^9^, ^10^ support discontinuation of use of race-specific equations, particularly in clinical settings. Although including race or genetic ancestry may minimally improve model fit,^32^ which may be of interest in certain epidemiologic research contexts, the use of race-specific equations has no demonstrable *clinical benefit* in improving diagnosis or classification of lung disease. Conversely, there is risk of clinical harm in continuing the use of race-specific equations for Black and other/mixed race patients who may be underdiagnosed for lung disease, as also shown in other studies.^7^^,^^20^^,^^21^ In particular, we found that a shift away from race-specific equations has a bigger impact on the mild end of the spectrum of GOLD classification, which may enable clinicians to catch more early-stage disease among Black patients, potentially preventing more disease progression. Moreover, these equations reinforce false assumptions about genetic differences between groups, while obscuring the role of environmental factors.^4^

The assumption that Black individuals have innately lower lung capacity dates back to slavery era observations.^33^ This assumption has carried through to modern medicine where biological differences are prioritized over social or environmental factors. Mounting evidence shows that racial/ethnic minority groups are disproportionately exposed to respiratory toxins via air pollution,^34^ occupational hazards,^35^ and harmful prenatal and childhood exposures, including preterm birth, very low birth weight, *in utero* smoke exposure, and childhood respiratory illnesses.^15^^,^^16^^,^^36^^,^^24^ Social stressors, such as community or family violence,^37^, ^38^, ^39^ and socioeconomic disadvantage^19^ have also been linked with worse lung function in early life and are likely interacting with genetic and epigenetic effects. These exposures are influenced by structural inequalities that shape living conditions among marginalized groups. Until we see strong and specific genetic evidence for innate racial differences in lung function, we believe it is not justified to use different criteria to diagnose non-White individuals. These social/environmental factors contribute to reduced lung function among minority racial/ethnic groups, and race-specific equations can mask their damaging effects. Use of ancestry instead of race in these equations would not solve this problem, as ancestral alleles track with the same environmental factors as race.^7^ Even if there were ancestral anthropometric differences, the consequences of these variations are not clearly linked to disease or dysfunction. Furthermore, not all individuals are likely to fit the average expectation for the group,^5^ particularly when the racial/ethnic populations used to create these equations originally were relatively small and not necessarily all healthy.^1^

## Interpretation

As this debate continues, race-specific equations are still recommended by the most recent US and European guidelines^14^ and are used in clinical care and pulmonary research worldwide. Although recent studies recommend the race-neutral GLI-Other equation for universal use,^7^^,^^8^ and the latest race-neutral GLI “Global” equation (based on an equally weighted and more balanced racial/ethnic dataset than GLI-Other),^40^ these equations are not race free as GLI-Other applies a universal race correction, and GLI-Global uses weights to balance racial/ethnic diversity. These approaches both still consider race in their formation, and thus assume racial differences in respiratory physiology in healthy individuals, as opposed to the race-free approach. Our findings suggest that race-free equations may serve equally well, at least for adults aged > 35 years.

The primary strengths of our study are its careful selection of healthy individuals for developing race-free reference equations by eliminating asymptomatic individuals with abnormal airway physiology and the consistent findings across two distinct and relatively large data sets with variable racial/ethnic proportions, using multiple modeling techniques. However, our findings should be interpreted in light of certain limitations. The Black population sample in the COPDGene data was relatively small, and the available data sets were not representative of all racial/ethnic groups. Socioeconomic and environmental factors were outside the scope of our study but are clearly important variables to examine in future studies.^19^^,^^35^^,^^41^^,^^42^ Although our analyses were cross-sectional, and thus cannot address directions of effect, we always used a distinct data set to test the fit of equations developed in an independent data set. Future studies should also use prospective longitudinal data to improve the predictive potential of these equations.

With rising awareness of structural racism and misconceptions about race in medicine,^43^, ^44^, ^45^, ^46^ this is a critical moment for pulmonary clinicians to reconsider the value of continuing to use race when interpreting spirometry measures. The effect of adding race as a covariate only marginally improves the fit for some models with the risk of introducing bias driven by environment and social factors. In light of these concerns, along with the large amount of unexplained variability and the dynamic nature of self-identified race, we maintain that continued use of race-specific equations is not justified. The findings presented here contribute to the growing literature that we hope will be considered when revisions to clinical guidelines are made.

## Funding/Support

This work was supported by the National Heart, Lung, and Blood Institute [Grants U01 HL089897 and U01 HL089856]. The COPDGene study^47^ is also supported by the COPD Foundation through contributions made to an Industry Advisory Committee composed of AstraZeneca, Bayer Pharmaceuticals, Boehringer Ingelheim, Genentech, GlaxoSmithKline, Novartis, Pfizer, and Sunovion. A. L. N. is supported by a UCSD Academic Senate Grant and a UCSD Division of Social Sciences Research Grant.

## Financial/Nonfinancial Disclosures

The authors have reported to *CHEST* the following: A. L. N. receives support from UCSD Academic Senate and UCSD Division of Social Sciences Research grants. A. A. D. declares speaker fees from Boehringer Ingelheim, outside of the submitted work. A. K. B. is funded by the National Institutes of Health (NIH) [Grants KL2TR002492 and UL1TR002494], unrelated to the current work. S. P. B. is supported by the NIH [Grants R01 HL151421 and NIH UH3HL155806], Nuvaira, and Sanofi; and has received royalties from Springer Humana and consulting fees from Boehringer Ingelheim, Sanofi/Regeneron, and IntegrityCE, unrelated to the current work. R. C. has been supported by grants from AstraZeneca, Regeneron, and Genentech; consulting fees from Regeneron, Genentech, Inogen, and Boehringer Ingelheim; and honoraria from GlaxoSmithKline, unrelated to current work. R. C. is also on the Board of Directors for the COPD Foundation and President of the Pulmonary Education and Research Foundation. None declared (B. B., E. A. R., A. W., A. L., C. R., G. K., K. A. Y., B. F., C. H., D. J. C.).

## Supplementary Data

e-Online Data

e-Online Data

e-Online Data
