# Supplementary material for: Race-Specific Spirometry Equations Do Not Improve Models of Dyspnea and Quantitative Chest CT Phenotypes
Source: Chest. 2023 Jul 26;164(6):1492–504. doi: 10.1016/j.chest.2023.07.019 (PMC10925545; doi:10.1016/j.chest.2023.07.019)
Supplement: e-Online Data [file mmc1.docx]

**Supplemental Materials**

Race-specific equations do not improve models of dyspnea and quantitative chest CT phenotypes.

Authors: Amy L. Non, PhD^1^, Barbara Bailey, PhD^2^, Surya P. Bhatt, MD^3^, Richard Casaburi, MD^4^, Elizabeth A. Regan, MD, PhD^5^, Angela Wang, MD^6^, Alfonso Limon, PhD^7^, Chantal Rabay, BS^1^, Alejandro A. Diaz, MD^8^, Arianne K. Baldomero, MD^9^, Greg Kinney, PhD^10^, Kendra A. Young, PhD^10^, Ben Felts, MS,^2^ Carol Hand, MS,^11^ Douglas J. Conrad^6^, MD*

Affiliations: ^1^Department of Anthropology, University of California San Diego, La Jolla, CA; ^2^Department of Mathematics and Statistics, San Diego State University, San Diego, CA; ^3^Division of Pulmonary, Allergy, and Critical Care Medicine, University of Alabama at Birmingham, Birmingham, AL.; ^4^Rehabilitation Clinical Trials Center, Lundquist Institute for Biomedical Innovation at Harbor-UCLA Medical Center, Torrance, CA; ^5^Division of Rheumatology and Department of Medicine, National Jewish Health, Denver, CO; ^6^Department of Medicine, University of California San Diego, La Jolla, CA

^7^Oneirix Labs, Carlsbad, CA; ^8^Division of Pulmonary and Critical Care Medicine, Brigham and Women’s Hospital, Harvard Medical School, Boston, MA; ^9^Pulmonary, Allergy, Critical Care and Sleep Medicine Section, Minneapolis VA Health Care System; ^10^Department of Epidemiology, Colorado School of Public Health, University of Colorado Anschutz Medical Campus, Aurora, CO; ^11^Advanced Mathematical Computing, San Diego, CA

Table of Contents

[Section 1. Formation and Characterization of Non-Smoking and Smoking Cohorts 3](#_Toc137719945)

[eAppendix 1. Methods for formation of study cohorts. 3](#_Toc137719946)

[e-Figure 1. Formation of study cohorts. 4](#_Toc137719947)

[e-Figure 2. Quantitative chest CT phenotypes in the never-smoker, healthy COPDGene cohort (“cg419”). 7](file:////Users/amynon/Dropbox/Lung%20Function%20Race%20Project/Race%20Spirometry%20Shared%20Draft/Chest%20Revision/UploadDocs(in%20progress)/Supp%20Figs_Tables%20V4%20TOC042923Chest.docx#_Toc137719948)

[e-Figure 3. Demographic and spirometric characteristics of the COPDGene and nh785 smoking cohorts. 8](file:////Users/amynon/Dropbox/Lung%20Function%20Race%20Project/Race%20Spirometry%20Shared%20Draft/Chest%20Revision/UploadDocs(in%20progress)/Supp%20Figs_Tables%20V4%20TOC042923Chest.docx#_Toc137719949)

[Section 2. Generation of Race-Specific and Race-Neutral FEV_1_ and FVC Prediction Models from Never-smoking, Healthy Cohorts. 9](#_Toc137719950)

[e-Table 1. Linear quantile regression model coefficients of predicted (median) log (FEV_1_) for NHANES (nh3700) and COPDGene (cg419) datasets. 9](file:////Users/amynon/Dropbox/Lung%20Function%20Race%20Project/Race%20Spirometry%20Shared%20Draft/Chest%20Revision/UploadDocs(in%20progress)/Supp%20Figs_Tables%20V4%20TOC042923Chest.docx#_Toc137719951)

[e-Table 2. Models of predicted log (FVC) median and 5^th^ percentile (LLN) quantile regression coefficients for NHANES (nh3700) and COPDGene (cg419) datasets 10](file:////Users/amynon/Dropbox/Lung%20Function%20Race%20Project/Race%20Spirometry%20Shared%20Draft/Chest%20Revision/UploadDocs(in%20progress)/Supp%20Figs_Tables%20V4%20TOC042923Chest.docx#_Toc137719952)

[e-Table 3. Building models of predicted log(FVC) median quantile regression coefficients for COPDGene (cg419) and NHANES (nh3700) datasets. 11](file:////Users/amynon/Dropbox/Lung%20Function%20Race%20Project/Race%20Spirometry%20Shared%20Draft/Chest%20Revision/UploadDocs(in%20progress)/Supp%20Figs_Tables%20V4%20TOC042923Chest.docx#_Toc137719953)

[Section 3. Comparison of Race-Specific and Race-Neutral FEV_1_ and FVC Prediction Models derived from Never-smoking, Healthy Cohorts. 12](#_Toc137719954)

[e-Figure 4. Identity plots of predicted and LLN FEV_1_ values comparing different race-specific models in both healthy never-smoking datasets 12](file:////Users/amynon/Dropbox/Lung%20Function%20Race%20Project/Race%20Spirometry%20Shared%20Draft/Chest%20Revision/UploadDocs(in%20progress)/Supp%20Figs_Tables%20V4%20TOC042923Chest.docx#_Toc137719955)

[e-Figure 5. Density plots of the differences between standard GLI predicted FEV_1_ and the LLN values and those derived from race-specific equations in healthy never-smokers. 13](file:////Users/amynon/Dropbox/Lung%20Function%20Race%20Project/Race%20Spirometry%20Shared%20Draft/Chest%20Revision/UploadDocs(in%20progress)/Supp%20Figs_Tables%20V4%20TOC042923Chest.docx#_Toc137719956)

[e-Figure 6. Identity plots of predicted and LLN FEV_1_ values comparing GLI-Race specific to Race-neutral values in both healthy, never smoker datasets 14](file:////Users/amynon/Dropbox/Lung%20Function%20Race%20Project/Race%20Spirometry%20Shared%20Draft/Chest%20Revision/UploadDocs(in%20progress)/Supp%20Figs_Tables%20V4%20TOC042923Chest.docx#_Toc137719957)

[e-Figure 7. Density plots of the differences between standard GLI-Race specific predicted FEV_1_ and the LLN values and those derived from race-neutral equations in healthy never-smokers. 15](file:////Users/amynon/Dropbox/Lung%20Function%20Race%20Project/Race%20Spirometry%20Shared%20Draft/Chest%20Revision/UploadDocs(in%20progress)/Supp%20Figs_Tables%20V4%20TOC042923Chest.docx#_Toc137719958)

[e-Figure 8. Density plots of differences of predicted and LLN FEV_1_ (ml) between race-neutral and race-free equations 16](file:////Users/amynon/Dropbox/Lung%20Function%20Race%20Project/Race%20Spirometry%20Shared%20Draft/Chest%20Revision/UploadDocs(in%20progress)/Supp%20Figs_Tables%20V4%20TOC042923Chest.docx#_Toc137719959)

[e-Figure 9. Measured FEV_1_ (L) vs ppFEV_1_ from race-specific and race-neutral equation values 17](file:////Users/amynon/Dropbox/Lung%20Function%20Race%20Project/Race%20Spirometry%20Shared%20Draft/Chest%20Revision/UploadDocs(in%20progress)/Supp%20Figs_Tables%20V4%20TOC042923Chest.docx#_Toc137719960)

[e-Figure 10. The effects of race in FEV_1_ models in the different NHANES race/ethnicity groups 18](file:////Users/amynon/Dropbox/Lung%20Function%20Race%20Project/Race%20Spirometry%20Shared%20Draft/Chest%20Revision/UploadDocs(in%20progress)/Supp%20Figs_Tables%20V4%20TOC042923Chest.docx#_Toc137719961)

[e-Figure 11. The FEV_1_ normalization equations using alternative anthropometric measures in place of height generate similar differences in ppFEV_1_ 19](file:////Users/amynon/Dropbox/Lung%20Function%20Race%20Project/Race%20Spirometry%20Shared%20Draft/Chest%20Revision/UploadDocs(in%20progress)/Supp%20Figs_Tables%20V4%20TOC042923Chest.docx#_Toc137719962)

[Section 4. Effects of New Race-specific and Race-neutral Models on ppFEV_1_ and GOLD Classification in COPDGene and nh785 smokers. 21](#_Toc137719963)

[e-Figure 12. Density plots of differences between the GLI reference and race-specific equation values for ppFEV_1_ in COPDGene smokers 21](file:////Users/amynon/Dropbox/Lung%20Function%20Race%20Project/Race%20Spirometry%20Shared%20Draft/Chest%20Revision/UploadDocs(in%20progress)/Supp%20Figs_Tables%20V4%20TOC042923Chest.docx#_Toc137719965)

[e-Figure 13. Density plots of differences between the GLI-Global race-neutral and the race-free ppFEV_1_ values in smoking cohorts 22](file:////Users/amynon/Dropbox/Lung%20Function%20Race%20Project/Race%20Spirometry%20Shared%20Draft/Chest%20Revision/UploadDocs(in%20progress)/Supp%20Figs_Tables%20V4%20TOC042923Chest.docx#_Toc137719967)

[Section 5. Quantitative Chest CT Phenotype and Dyspnea Models in Smokers 23](#_Toc137719968)

[e-Figure 14. Race-specific sensitivity analysis of quantitative chest CT phenotypes 23](file:////Users/amynon/Dropbox/Lung%20Function%20Race%20Project/Race%20Spirometry%20Shared%20Draft/Chest%20Revision/UploadDocs(in%20progress)/Supp%20Figs_Tables%20V4%20TOC042923Chest.docx#_Toc137719969)

**e-Table 4. Race specific multivariable logistic regression analysis and Random Forests models of quantitative chest CT phenotypes ……………………………………………………………………………….24**

[e-Figure 15. Race specific performance of race-specific and race-neutral equation derived ppFEV_1_ values ability to identify abnormal mMRC values using three distinct modeling approaches. 27](file:////Users/amynon/Dropbox/Lung%20Function%20Race%20Project/Race%20Spirometry%20Shared%20Draft/Chest%20Revision/UploadDocs(in%20progress)/Supp%20Figs_Tables%20V4%20TOC042923Chest.docx#_Toc137719970)

[e-Table 5. Race-specific multivariable logistic regression analysis of dyspnea (MMRC > 1) 28](file:////Users/amynon/Dropbox/Lung%20Function%20Race%20Project/Race%20Spirometry%20Shared%20Draft/Chest%20Revision/UploadDocs(in%20progress)/Supp%20Figs_Tables%20V4%20TOC042923Chest.docx#_Toc137719971)

[e-Figure 16. Comparisons of race-neutral GLI-Global and race-free nh3700_AGH models in two distinct healthy populations………………………………………………………………………………………………..31](file:////Users/amynon/Dropbox/Lung%20Function%20Race%20Project/Race%20Spirometry%20Shared%20Draft/Chest%20Revision/UploadDocs(in%20progress)/Supp%20Figs_Tables%20V4%20TOC042923Chest.docx#_Toc137719970)

#

# **Section 1. Formation and Characterization of Non-Smoking and Smoking Cohorts**

## **e-Appendix 1. Methods for formation of study cohorts.**

*nh3700 never-smoking healthy cohort*: Demographic, spirometric, and anthropometric data were downloaded from the NHANES website from 2007-2012 (https://www.cdc.gov/nchs/nhanes). The NHANES survey and consent documents were approved by the CDC Institutional Review Board. There were 20,050 participants with completed spirometry from five self-identified groups: Mexican American, Other Hispanic, Non-Hispanic White, Non-Hispanic African American and Other/Mixed Race. This dataset was filtered to exclude participants who smoked or have respiratory symptoms to create a never-smoking healthy sample (see Figure E1A). To appropriately use *linear* quantile regression modeling and to more closely match the COPDGene comparator cohort, the analysis restricted this cohort to adults greater than age 35 years. Outliers or individuals with asymptomatic physiologic dysfunction were identified and excluded using the Mahalanobis distance metric (alpha set to 0.2),^20^ resulting in an analytical sample size of n=3700 individuals (hereafter referred to as nh3700, Table 1 and Figure E1A).

*cg419 never-smoking healthy cohort*: The Genetic Epidemiology of COPD (COPDGene) Study is a multicenter cohort of current and former smokers (with at least ten pack-years smoking history) and non-smokers, aged 40-80 with extensive clinical, demographic, radiographic, and multi-omic associated data from self-identified Non-Hispanic Black and Non-Hispanic White individuals.^21^ Post-bronchodilator spirometry data were collected using published protocols,^21^ and inspiratory and expiratory quantitative imaging data (Chest CT) were acquired using standardized protocols.^22^ The institutional review boards of the 21 participating institutions approved this study and all participants provided written informed consent. The COPDGene Study recruited over 500 never-smoking control subjects who underwent the same clinical, radiographic and physiologic evaluation as the smoking cohort. Among these were 442 participants with complete basic demographic data, at least one acceptable spirometry, and a quantitative inspiratory chest CT. Outliers were identified and excluded using the Mahalanobis distance,^20^ resulting in an analytical sample of 419 participants (hereafter referred to as cg419, Table 1 and Figure E1B).

*Smoking cohorts:* Two smoking populations were used to assess the impact of race-specific and race-neutral equations in assessing disease severity in smoking populations. The first dataset, the *nh785 ex-smokers* (n=785 individuals)*,* was derived from all NHANES participants (2007-2012) with acceptable spirometry data and basic demographics and a heavy smoking history (see Figure E1C for definition). *COPDGene Phase I smokers* consisted of all Phase I COPDGene study participants^23^ with a cigarette smoking history >10 pack-years and complete demographic data, at least one acceptable spirometry measure and a quantitative inspiratory chest CT scan (n= 9419).

## **e-Figure 1. Formation of study cohorts.**

**
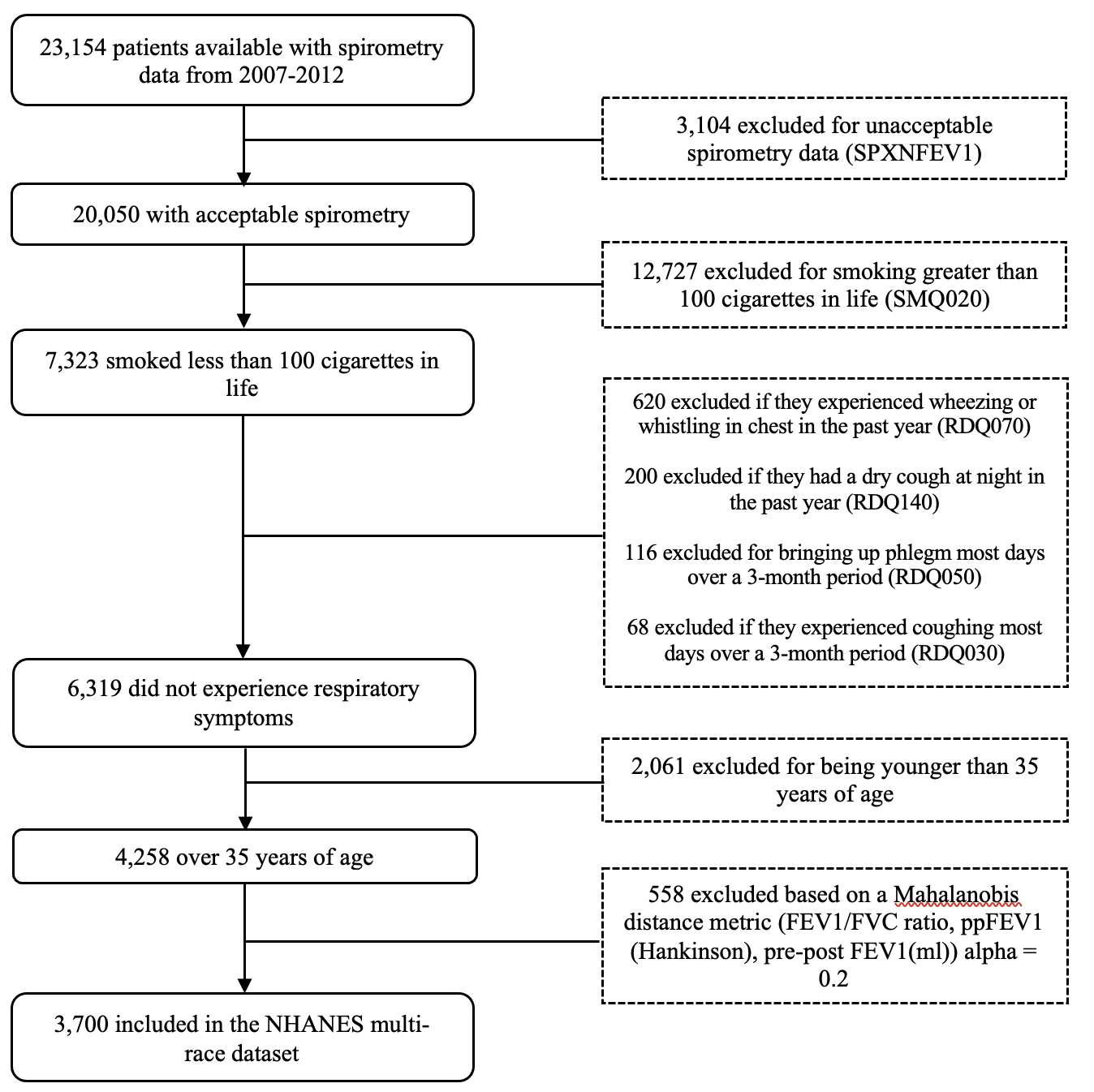
A). NHANES 2007-12 Healthy, never-smokers (“nh3700”)**

e-Figure 1A. Formation of the NHANES healthy, never smoker cohort, “nh3700”. Demographic and respiratory questionnaire data from over 20,000 NHANES participants with spirometry were downloaded (NHANES website). This group was filtered to exclude those with any significant lifetime smoking, any respiratory symptoms (i.e. answered no to questions on smoking (SMG020), or symptoms of wheezing (RDQ070) or dry cough (RDQ140) in the past year, consistent phlegm production (RDQ050) or chronic cough (RDQ031) over three months), and missing demographic data. To appropriately use linear quantile regression modeling, participants over 35 years of age were selected from all participants not missing height, age, gender and racial identity data, resulting in a cohort of 4258 individuals. The Mahalanobis distant metric was used to exclude 558 participant outliers in an unbiased manner using an alpha of 0.2. Input data included ppFEV_1_, FEV_1_/FVC ratio, and the bronchodilator response in ml. If a post-bronchodilator response was available, it was used, otherwise pre-bronchodilator values were used. The resulting 3700 healthy, never smokers comprised the nh3700 cohort.

**B. COPDGene healthy, never-smokers (“cg419”)**

446 never smokers with basic demographics, at least one ATS-acceptable spirometry, and one qCT at total lung capacity

27 excluded from “Normals” based on Mahalanobis distance of .025 using the five variables (ppFEV_1_, FEV_1_/FVC ratio, delta FEV1 (ml), pctEmphysema_Thirona, Pi10)

419 never smokers included

e-Figure 1B. Formation of healthy, never-smoker COPDGene cohort, “cg419”, Over 500 never smokers were recruited during COPDGene Phase I and II. 446 had complete demographics (age, gender, height and self-identified race), one acceptable spirometry and one quantitative chest CT at total lung capacity. An unbiased approach to exclude outliers used the Mahalanobis distance metric and an alpha parameter of .025. Input data included ppFEV_1_, FEV_1_/FVC ratio, the post-bronchodilator response in ml, percent emphysema and Pi10 defined previously. There were 419 participants after excluding 27 which comprised the cg419 healthy never-smoker cohort.

**C. NHANES 2007-12 ex-Smoker Cohort (“nh785 ex-Smokers”)**


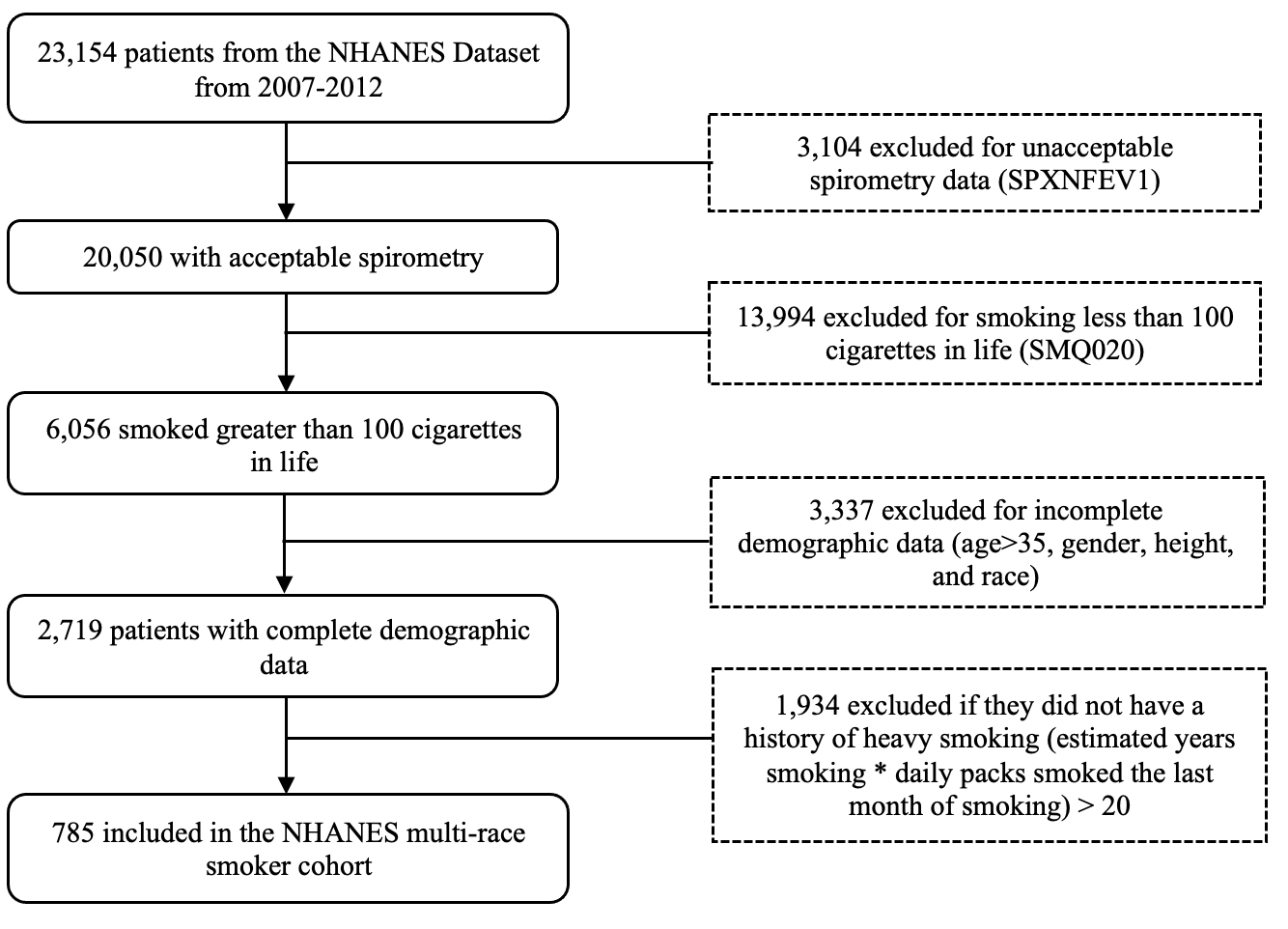


e-Figure 1C. Formation of the NHANES cohort, “nh785 ex-smokers”. Demographic and respiratory questionnaire data from over 20,000 NHANES participants with spirometry were downloaded (NHANES website). All of these participants were ex-smokers. This group was filtered to exclude those who smoked less than 100 cigarettes in their lifetime, or who had incomplete demographic data (age, gender, height and self-identified race or who were under the age of 35. The heaviest smokers were identified by having a product of the estimated number of years smoked by the number of packs smoked during the last month of smoking to be greater than 20. The resulting 785 smokers were the heaviest group of ex-smokers in the data and comprised the nh785 smoking cohort.

## **e-Figure 2: Quantitative chest CT phenotypes in the never-smoker, healthy COPDGene cohort (“cg419”).**

e-Figure 2. Quantitative Chest CT phenotypes of A) percent Emphysema, B) percent Air Trapping and C) Pi10 are compared between Black and White participants in the healthy COPDGene never smoking cohort (cg419). Notch plots demonstrate significantly lower average percent emphysema and higher Pi10 measures in Black relative to White participants. The percent emphysema is the percent of voxels that have a density < -950 HU on the CT scan at full inspiration. The percent air trapping is the percent of voxels that have a density < -856 HU on the CT scan at full expiration. The Pi10 is a measure of airway wall thickness and is the square root of airway wall thickness with an internal diameter of 10mm (1). The gray lines mark the ULN for each CT phenotype used. Horizontal gray lines indicate cutoffs for abnormal values of these measures (2). Black participants demonstrated higher Pi10 and lower percent emphysema values.


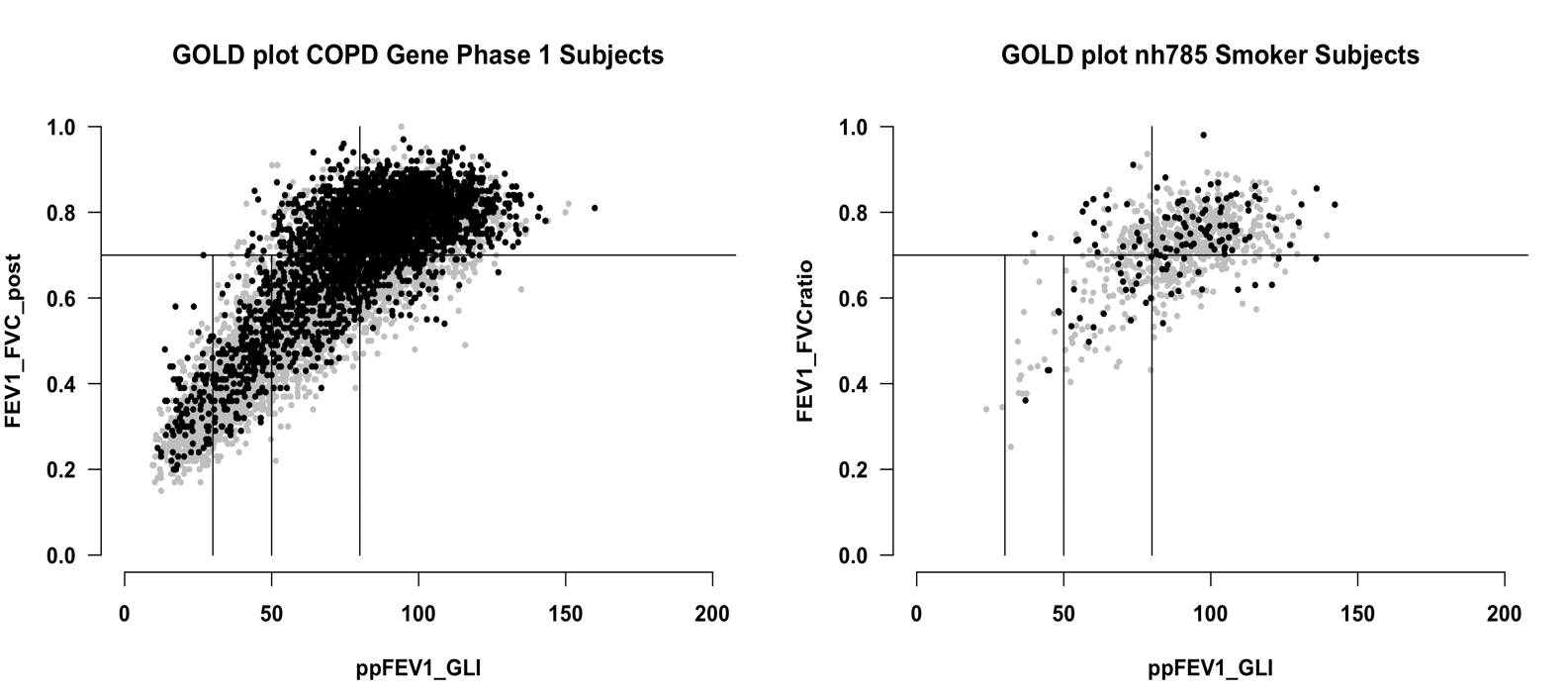

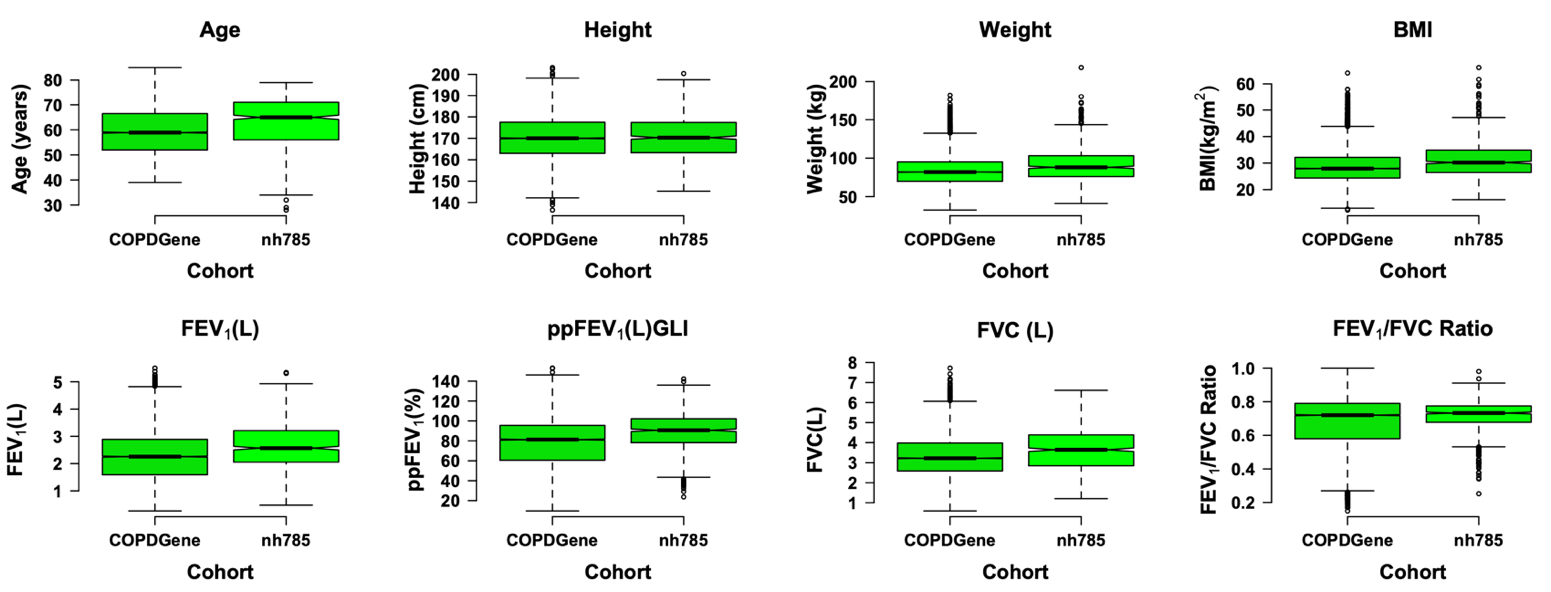


**B.**

**A.**

## **e-Figure 3: Demographic and spirometric characteristics of the COPDGene and nh785 smoking cohorts.**


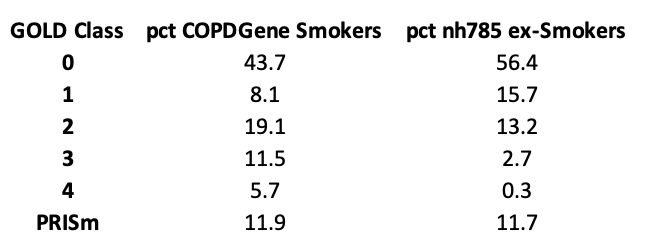


e-Figure 3. Demographic and spirometric characteristics of the COPDGene and NHANES (2007-12) smoking cohorts. A) Notch plots demonstrate significant differences between cohorts by age, height, weight, BMI, FEV1, ppFEV1 (estimated from GLI race-specific equations), and FVC and FEV_1_/ FVC ratio. B) GOLD plot demonstrates the distribution of the COPDGene Phase 1 smoking participants in each GOLD class based on ppFEV_1_ and FEV_1_:FVC ratios. GOLD plot of NHANES Black and White participants with a heavy smoking history. Black dots indicate Black participants, gray dots indicate White participants. The table E3C shows the percent of each cohort the different GOLD classes. GOLD 0 designates smokers with FEV_1_:FVC ratios > .7 and ppFEV_1_ >80%. Overall, despite being the older cohort on average, the nh785 cohort appears to have better lung function, with higher lung function measures, and fewer individuals in GOLD stages above 1 indicating milder lung disease in this smoking cohort.

**C.**

# **Section 2. Generation of Race-Specific and Race-Neutral FEV_1_ and FVC Prediction Models from Never-smoking, Healthy Cohorts.**

## **e-Table 1. Linear quantile regression model coefficients of predicted (median) log (FEV_1_) for NHANES (nh3700) and COPDGene (cg419) datasets.**

|  | **β0**  **(Intercept)** | **β1**  **(Age, years)** | **β2**  **(Female gender)** | **β3**  **(Height, cm)** | **β4**  **(Race)** | **R1** |
| --- | --- | --- | --- | --- | --- | --- |
| **NHANES log (FEV_1_)** |  |  |  |  |  |  |
| Median-Race alone (White) | 1.52017 |  |  |  | ref | 0.035 |
| Black |  |  |  |  | -0.17418 |  |
| Mexican American |  |  |  |  | -0.10044 |  |
| Hispanic Other |  |  |  |  | -0.10602 |  |
| Other/Mixed |  |  |  |  | -0.16511 |  |
| Median predicted – Age | 1.94020 | -0.00977 |  |  |  | 0.151 |
| Median predicted – Gender | 1.54266 |  | -0.31859 |  |  | 0.239 |
| Median predicted – Height | -2.10635 |  |  | 0.01900 |  | 0.316 |
| Median predicted – Age, Height | -1.32514 | -0.00929 |  | 0.01721 |  | 0.444 |
| Median predicted – Age, Gender | 1.79370 | -0.01091 | -0.32064 |  |  | 0.400 |
| **COPDGene log (FEV_1_)** |  |  |  |  |  |  |
| Median predicted – Race (White) | 1.07090 |  |  |  | ref | 0.012 |
| Median predicted – Race (Black) |  |  |  |  | -0.10658 |  |
| Median predicted – Age | 1.85737 | -0.01395 |  |  |  | 0.118 |
| Median predicted – Gender | 1.26469 |  | -0.37106 |  |  | 0.277 |
| Median predicted – Height | -2.34906 |  |  | 0.02013 |  | 0.360 |
| Median predicted – Age, Height | -1.48388 | -0.00893 |  | 0.01810 |  | 0.430 |
| Median predicted – Age, Gender | 2.15235 | -0.01080 | -0.35111 |  |  | 0.393 |

e-Table 1. The coefficients used in building the linear quantile regression models for the predicted FEV_1_. The predicted FEV_1_ (i.e. median quantile of the log FEV_1_) is modeled with each covariate (age, race, gender, height) individually, and also in combinations. The equation for predicted FEV_1_ is as follows: predicted FEV_1_ (L)= e^(β0 + Age* β1 + Gender code term * β2 + Height* β3 +Race code term* β4)^. The log FEV_1_ was modeled since it minimized the unexplained variability as assessed by higher R1 values. In both cohorts, if a post bronchodilator FEV_1_ value was available it was used in the modeling. In models of the cg419 data, the race code term for Black race is 1 and 0 for White race. In nh3700 data, race is modeled with NHW (0) as the reference group, and other racial/ethnic groups were coded as 1 if present and multiplied by the corresponding race coefficient. In both cohorts, male and female gender are coded 0 and 1 respectively.

e-Table 2. Linear Quantile Regression Coefficients for the predicted (median quantile) and the LLN (5^th^ quantile) models of log(FVC). The log(FVC) is modeled with the combined covariates (age, race, gender, height) to generate the race specific equations predFVC_nh3700_AGHR or LLNFVC_cg419_AGHR. Similarly, the log(FVC) is modeled with covariates age, gender, height to generate the race free equations predFVC_nh3700_AGH and LLNFVC_cg419_AGH. The equation for the FVC is as follows: FVC (L) = e^(β0 + Age* β1 + Gender code term * β2 + Height* β3 +Race code term* β4)^. The log FVC was modeled since it minimized the unexplained variability as assessed by higher R1 values. In both cohorts, if a post bronchodilator FVC value was available it was used in the modeling. In models of the cg419 data, the race code term for Black race is 1 and 0 for White race. In nh3700 data, race is modeled with NHW (0) as the reference group, and other racial/ethnic groups were coded as 1 if present and multiplied by the corresponding race coefficient. In both cohorts, male and female gender are coded 0 and 1 respectively. Abbreviations: FVC=Forced Vital Capacity; AGH=models including age, gender, and height only; AGHR=models including age, gender, height, and race/ethnicity.

## **e-Table 2. Models of predicted log (FVC) median and 5^th^ percentile (LLN) quantile regression coefficients for NHANES (nh3700) and COPDGene (cg419) datasets**

|  | **β0**  **(Intercept)** | **β1**  **(Age, years)** | **β2**  **(Female gender)** | **β3**  **(Height, cm)** | **β4**  **(Race)** | **R1** |
| --- | --- | --- | --- | --- | --- | --- |
| **NHANES – log (FVC)** |  |  |  |  |  |  |
| Median predicted – AGH | -0.44468 | -0.00789 | -0.14771 | 0.01335 | -- | 0.472 |
| Median predicted – AGHR (White) | -0.49261 | -0.0076 | -0.14207 | 0.01383 | ref | 0.547 |
| AGHR (Black) |  |  |  |  | -0.18799 |  |
| AGHR (Mexican American) |  |  |  |  | 0.00049 |  |
| AGHR (Hispanic Other) |  |  |  |  | -0.03607 |  |
| AGHR (Other/Mixed) |  |  |  |  | -0.10671 |  |
| 5th percentile – AGH | -0.41954 | -0.00834 | -0.18078 | 0.01185 | -- | -- |
| 5th percentile – AGHR (White) | -0.6777 | -0.00937 | -0.1452 | 0.01418 | ref | -- |
| Black |  |  |  |  | -0.20623 |  |
| Mexican American |  |  |  |  | 0.00247 |  |
| Hispanic Other |  |  |  |  | -0.02700 |  |
| Other/Mixed |  |  |  |  | -0.07427 |  |
| **COPDGene - log (FVC)** |  |  |  |  |  |  |
| Median predicted – AGH | -0.33902 | -0.00747 | -0.15802 | 0.01272 | -- | 0.452 |
| Median predicted – AGHR (White) | -0.34664 | -0.00839 | -0.1503 | 0.01324 | ref |  |
| Median predicted – AGHR (Black) |  |  |  |  | -0.18547 | 0.520 |
| 5th percentile – AGH | -0.31082 | -0.00731 | -0.14842 | 0.01101 | -- | -- |
| 5th percentile – AGHR (White) | 0.11081 | -0.01186 | -0.16323 | 0.01058 | ref | -- |
| 5th percentile – AGHR (Black) |  |  |  |  | -0.21861 |  |

## **e-Table 3. Building models of predicted log(FVC) median quantile regression coefficients for COPDGene (cg419) and NHANES (nh3700) datasets.**

|  | **β0**  **(Intercept)** | | **β1**  **(Age, years)** | | **β2**  **(Gender, Female)** | | **β3**  **(Height cm)** | | **β4**  **(Race)** | | **R1** | |  |
| --- | --- | --- | --- | --- | --- | --- | --- | --- | --- | --- | --- | --- | --- |
| **NHANES log (FVC)** |  | |  | |  | |  | |  | |  | |  |
| Median - Race alone (White) | 1.36021 | |  | |  | |  | | ref | | 0.045 | |  |
| AGHR (Black) |  | |  | |  | |  | | -0.20774 | |  | |  |
| AGHR (Mexican American) |  | |  | |  | |  | | -0.08652 | |  | |  |
| AGHR (Hispanic Other) |  | |  | |  | |  | | -0.14026 | |  | |  |
| AGHR (Other/Mixed) |  | |  | |  | |  | | -0.16568 | |  | |  |
| Median predicted – Age | 1.82824 | | -0.01107 | |  | |  | |  | | 0.112 | |  |
| Median predicted – Gender | 1.48704 | |  | | -0.36309 | |  | |  | | 0.258 | |  |
| Median predicted – Height | -1.99758 | |  | |  | | 0.01973 | |  | | 0.353 | |  |
| Median predicted – Age, Height | -1.39308 | | -0.00739 | |  | | 0.0184 | |  | | 0.437 | |  |
| Median predicted – Age, Gender | 1.93648 | | -0.00899 | | -0.33873 | |  | |  | | 0.369 | |  |
|  |  | |  | |  | |  | |  | |  | |  |
| **COPDGene log (FVC)** |  | |  | |  | |  | |  | |  | |  |
| Median predicted – Race (White) |  | |  | |  | |  | | ref | |  | |  |
| Median predicted – Race (Black) | 1.27815 | |  | |  | |  | | -0.12821 | | 0.015 | |  |
| Median predicted – Age | 1.93601 | | -0.01139 | |  | |  | |  | | 0.086 | |  |
| Median predicted – Gender | 1.47842 | |  | | -0.36295 | |  | |  | | 0.287 | |  |
| Median predicted – Height | -2.32433 | |  | |  | | 0.02135 | |  | | 0.380 | |  |
| Median predicted – Age, Height | -1.6437 | | -0.00616 | |  | | 0.01947 | |  | | 0.424 | |  |
| Median predicted – Age, Gender | 2.02629 | | -0.0096 | | -0.32426 | |  | |  | | 0.358 | |  |
|  | |  | |  | |  | | e-Table 3. The Coefficients used in Building Linear Quantile Regression Models for the predicted FVC. The predicted FVC is modeled with each covariate (age, race, gender, height) individually, and also in combinations. The equation for predicted (median) FVC is as follows: predicted FVC (L) = e^(β0 + Age* β1 + Gender code term * β2 + Height* β3 +Race code term* β4)^. The log FEV_1_ was modeled since it minimized the unexplained variability as assessed by higher R1 values. In both cohorts, if a post bronchodilator FVC value was available it was used in the modeling. In models of the cg419 data, the race code term for Black race is 1 and 0 for White race. In nh3700 data, race is modeled with NHW (0) as the reference group, and other racial/ethnic groups were coded as 1 if present and multiplied by the corresponding race coefficient. Abbreviations: FVC=Forced vital capacity | |  | |  | |

# **Section 3. Comparison of Race-Specific and Race-Neutral FEV_1_ and FVC Prediction Models derived from Never-smoking, Healthy Cohorts.**

## **e-Figure 4. Identity plots of predicted and LLN FEV_1_ values comparing different race-specific models in both healthy never-smoking datasets**


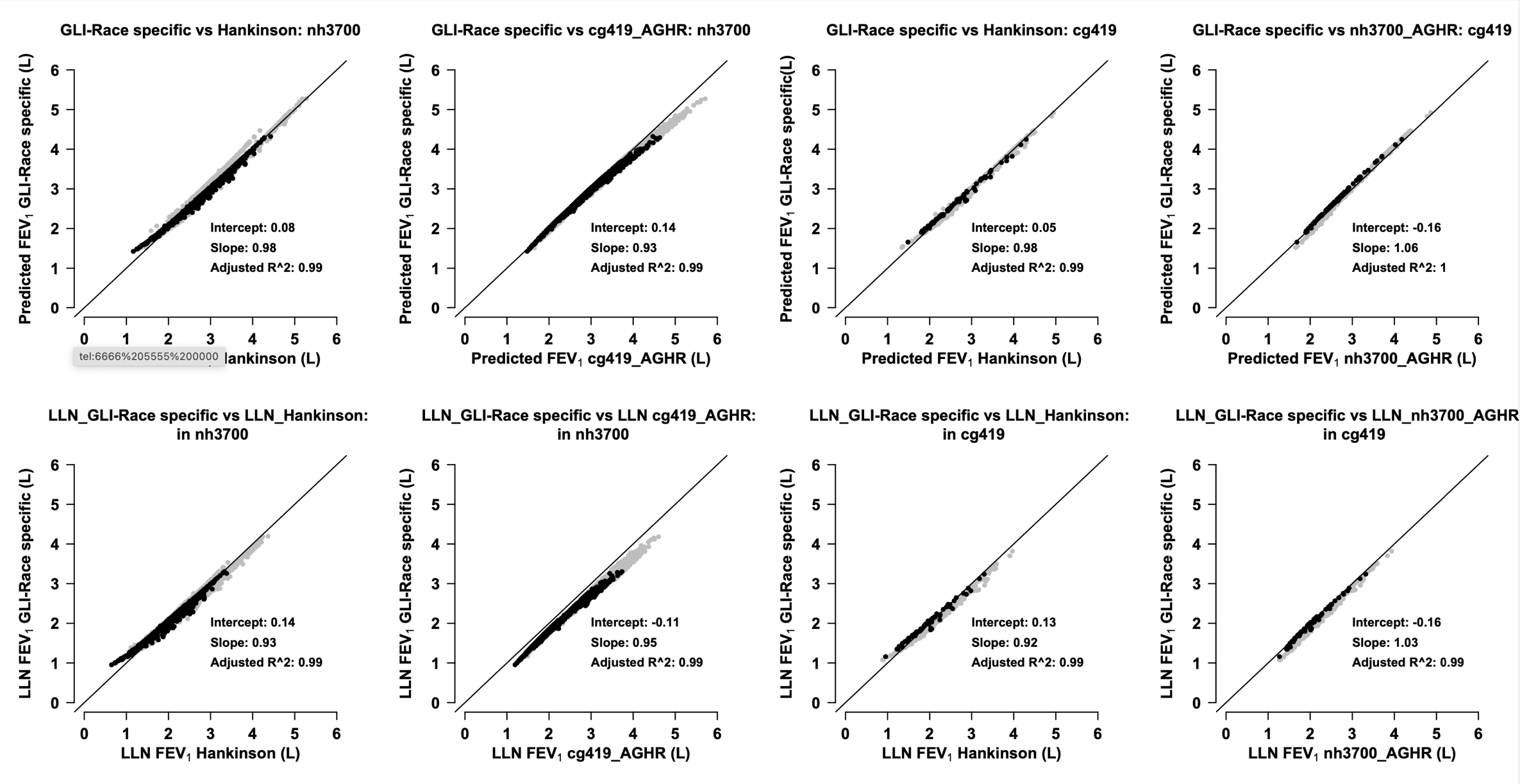


e-Figure 4. Identity plots of the predicted FEV_1_ (top row) and the FEV_1_ lower limit of normal (LLN) values (bottom row) derived from the race-specific equations relative to the GLI reference equations, including the Hankinson equation and the race-specific equations generated from the healthy never smokers in COPDGene (cg419) and NHANES datasets (nh3700). Black dots indicate Black participants, grey dots indicate White participants in both the healthy never smoking nh3700 (left) and cg419 (right) cohorts. *Close correlations between all race-specific estimates across datasets validates the healthy never-smoking datasets and the modeling approach used in these studies.*

e-Figure 5. Density plots of the differences between GLI-Race specific values and the values derived from race-specific equations in the nh3700 (A) and cg419 (B) healthy never smoker cohorts. In each section, the predicted FEV_1_ and the LLN of the FEV_1_ are shown in the top and bottom rows respectively. Differences were calculated by subtracting the predicted FEV_1_ (or LLN) of the stated model from the GLI-Race specific value. The black, gray and red lines demonstrate the densities of the Black, White and the total population respectively.


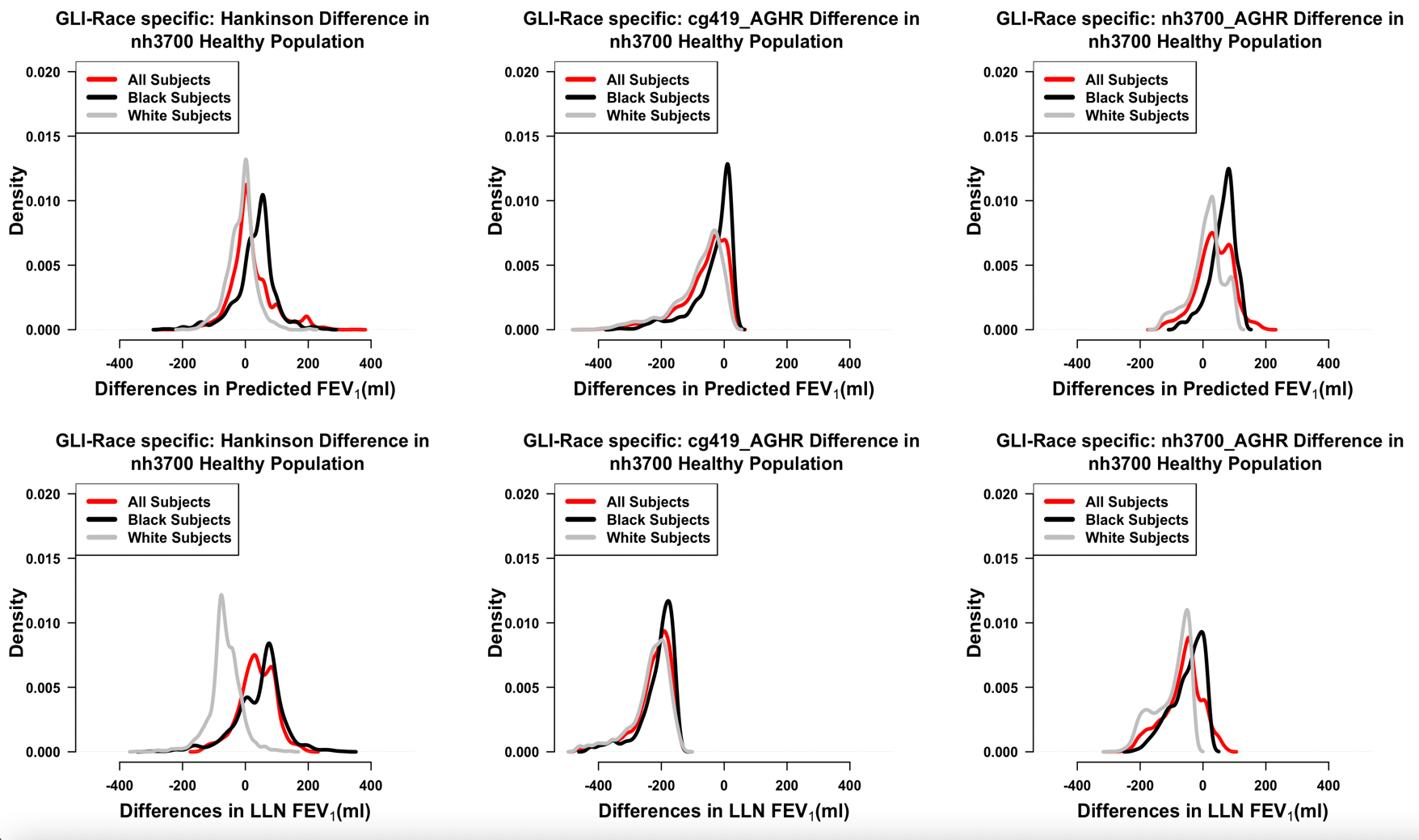

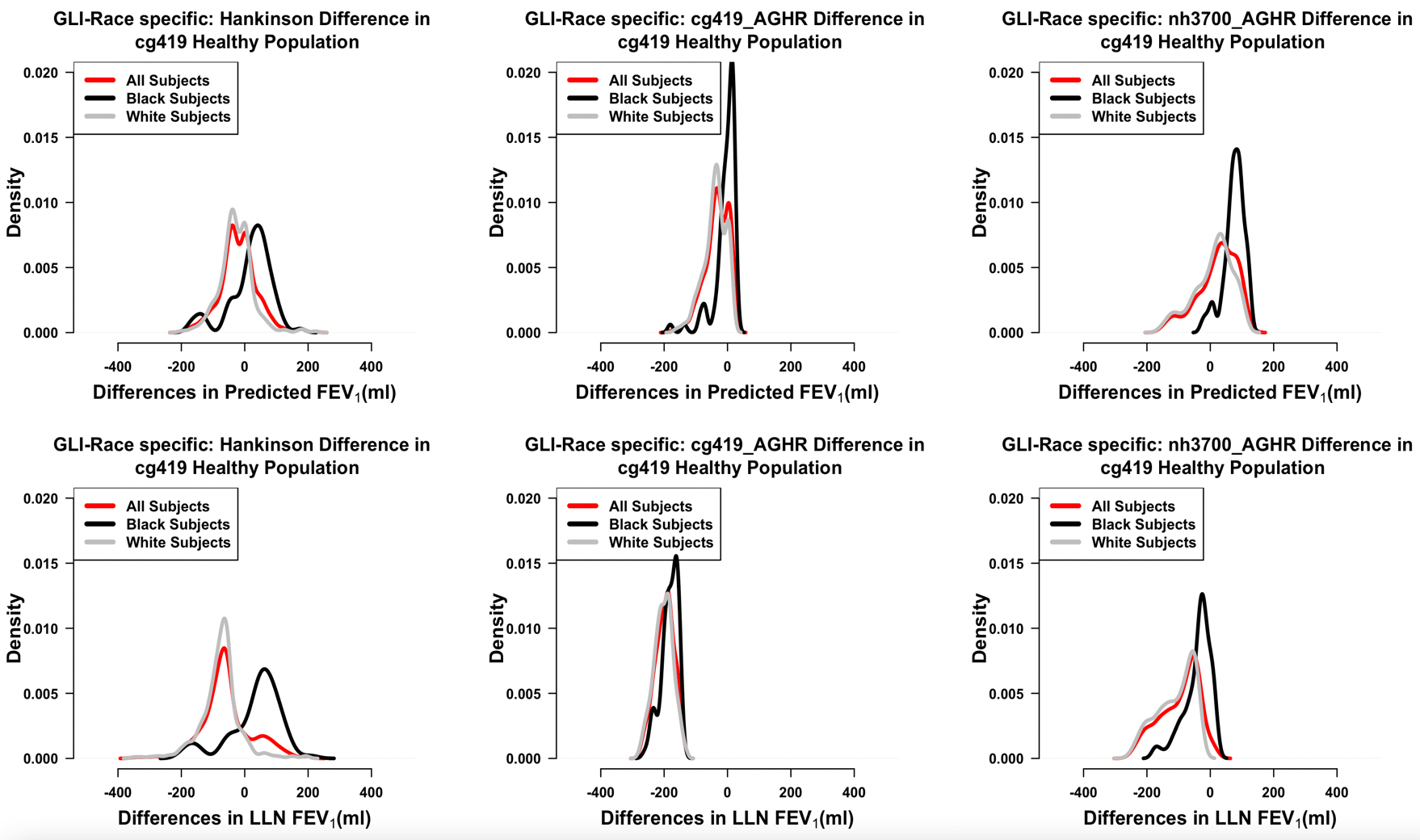


**A.**

**B.**

## **e-Figure 5. Density plots of the differences between standard GLI predicted FEV_1_ and the LLN values and those derived from race-specific equations in healthy never-smokers.**

## **e-Figure 6. Identity plots of predicted and LLN FEV_1_ values comparing GLI-Race specific to Race-neutral values in both healthy, never smoker datasets**


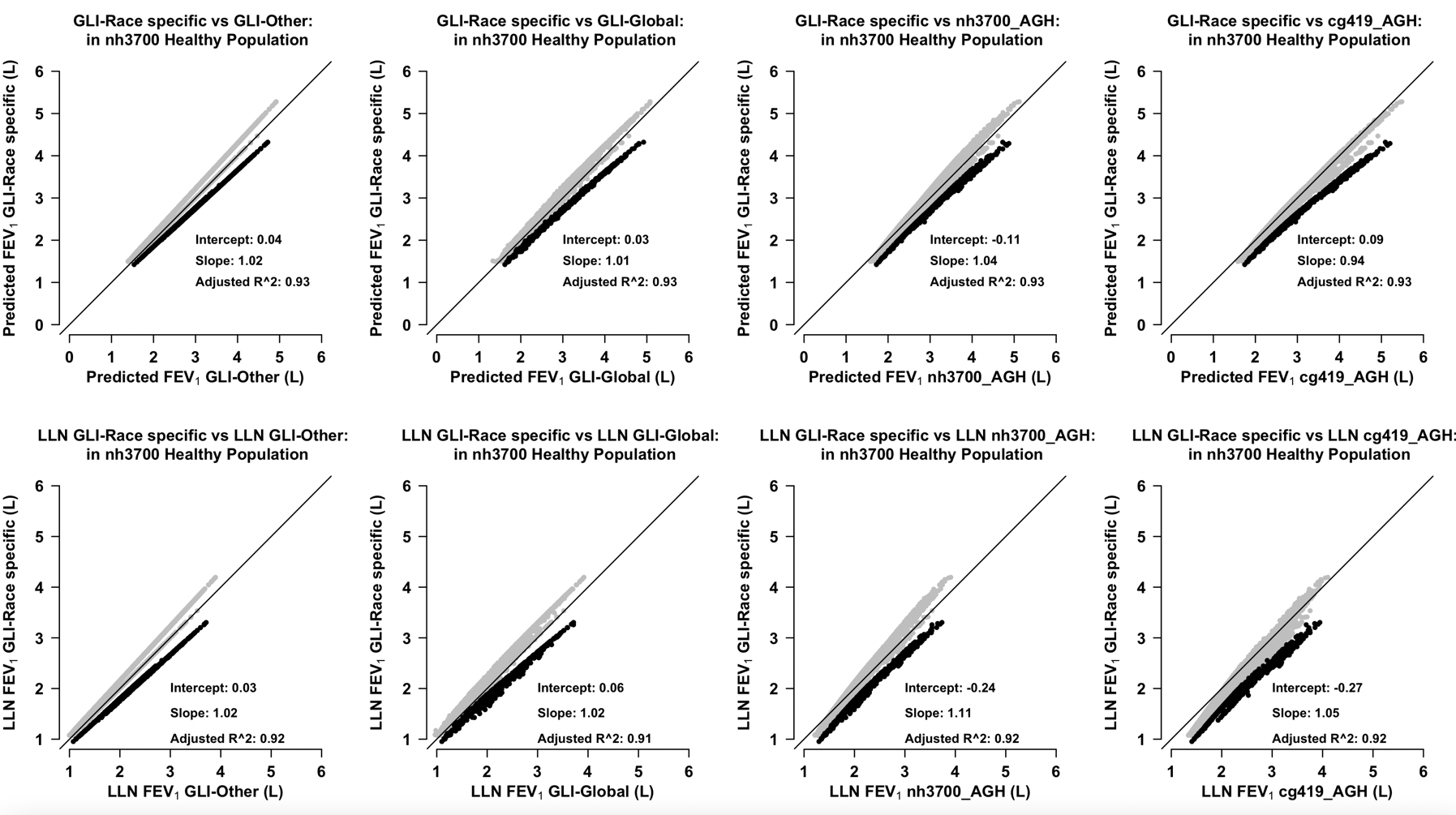

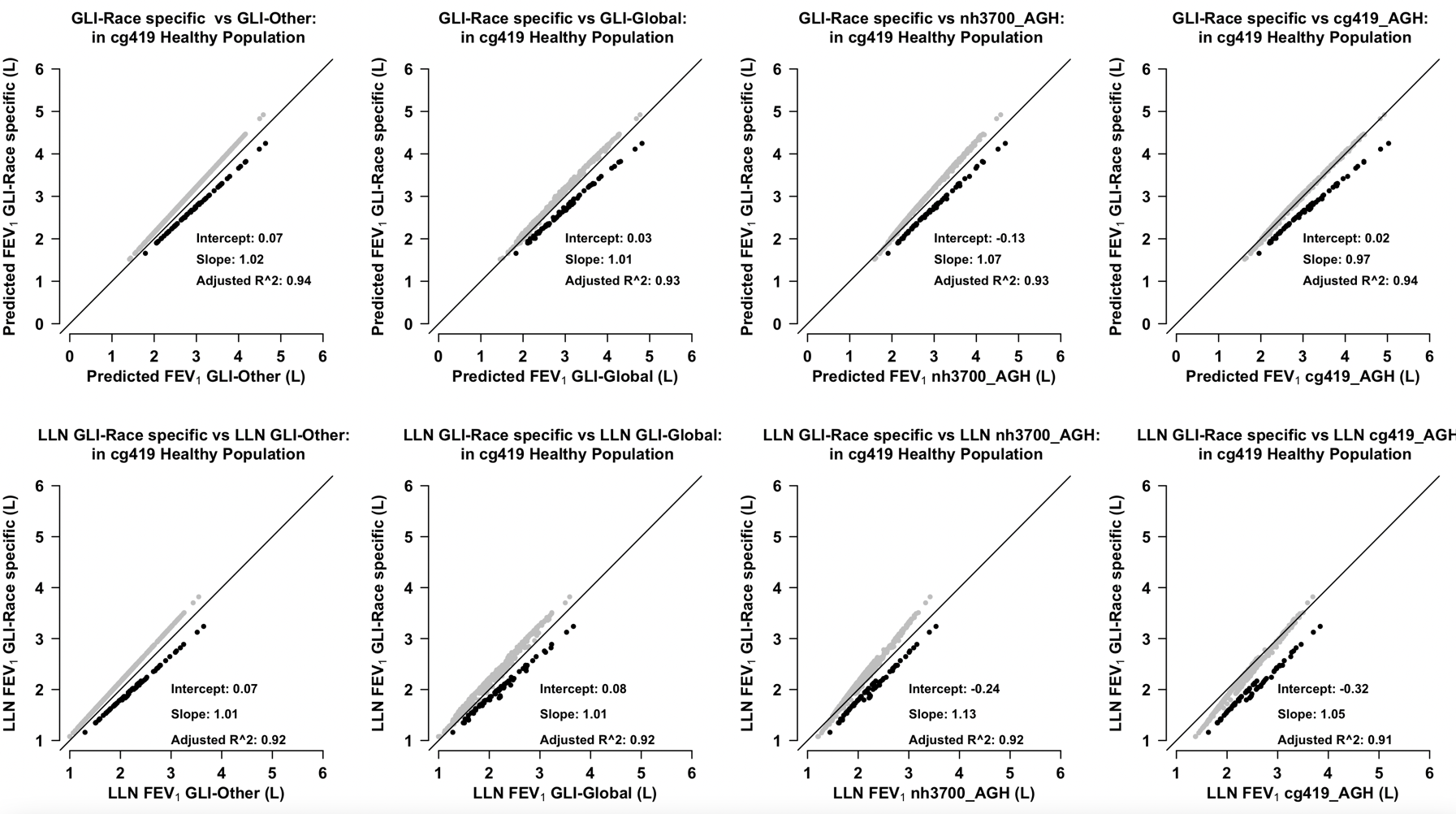


**A.**

**B.**

e-Figure 6. Identity plots of the predicted FEV_1_ and FEV_1_ LLN values in liters of the nh3700 (A) and cg419 (B) healthy never smoker cohorts comparing values from the GLI-Race specific reference equations to the values derived from race-neutral (GLI-Other and GLI-Global) or race-free (cg419_AGH and nh3700_AGH) equations. Black dots indicate Black participants, grey dots indicate White participants. In each section, the predicted FEV_1_ and the LLN of the FEV_1_ are shown in the top and bottom rows respectively. The race-neutral and race-free equations generated higher predicted FEV_1_ and LLN values than the race-specific equations in Black participants (which corresponds to lower percent predicted values and more participants classified as abnormal) in both healthy never smoking cohorts.


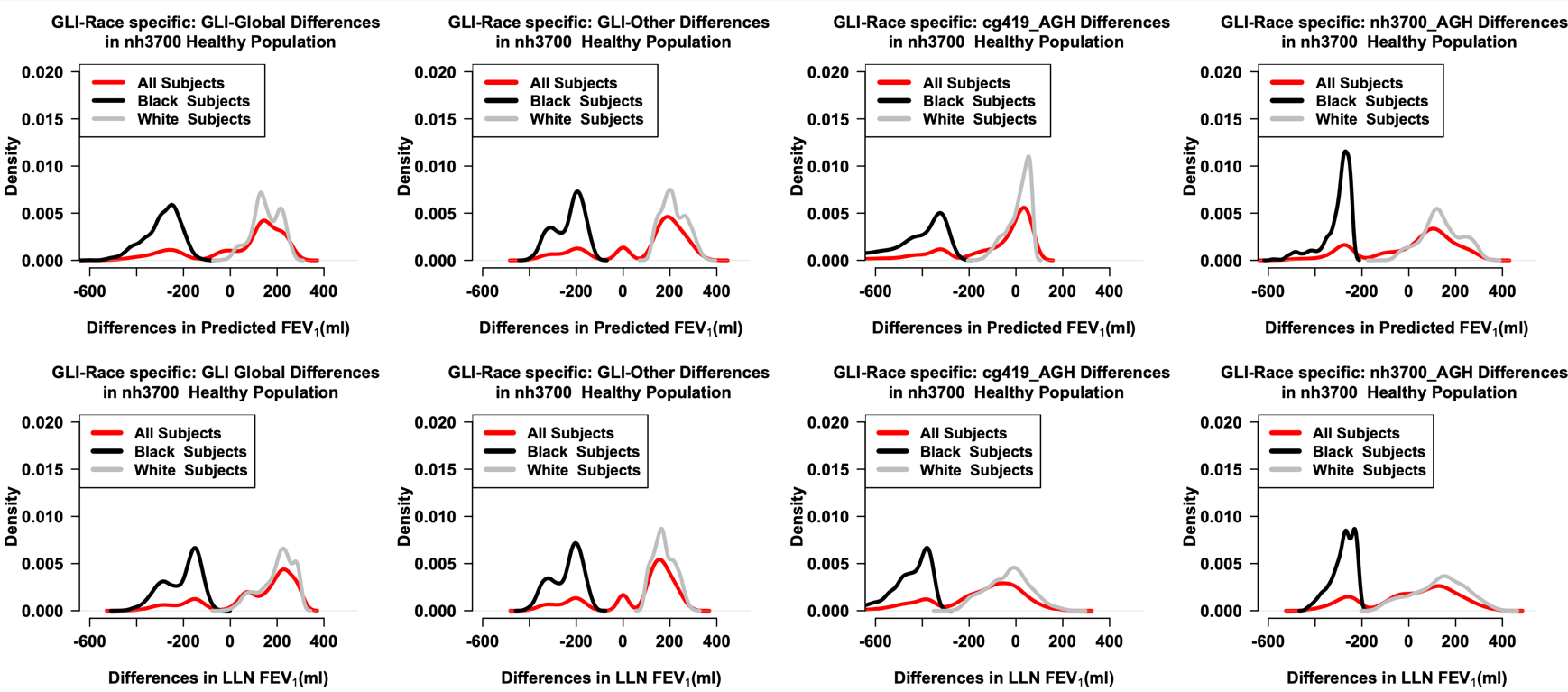

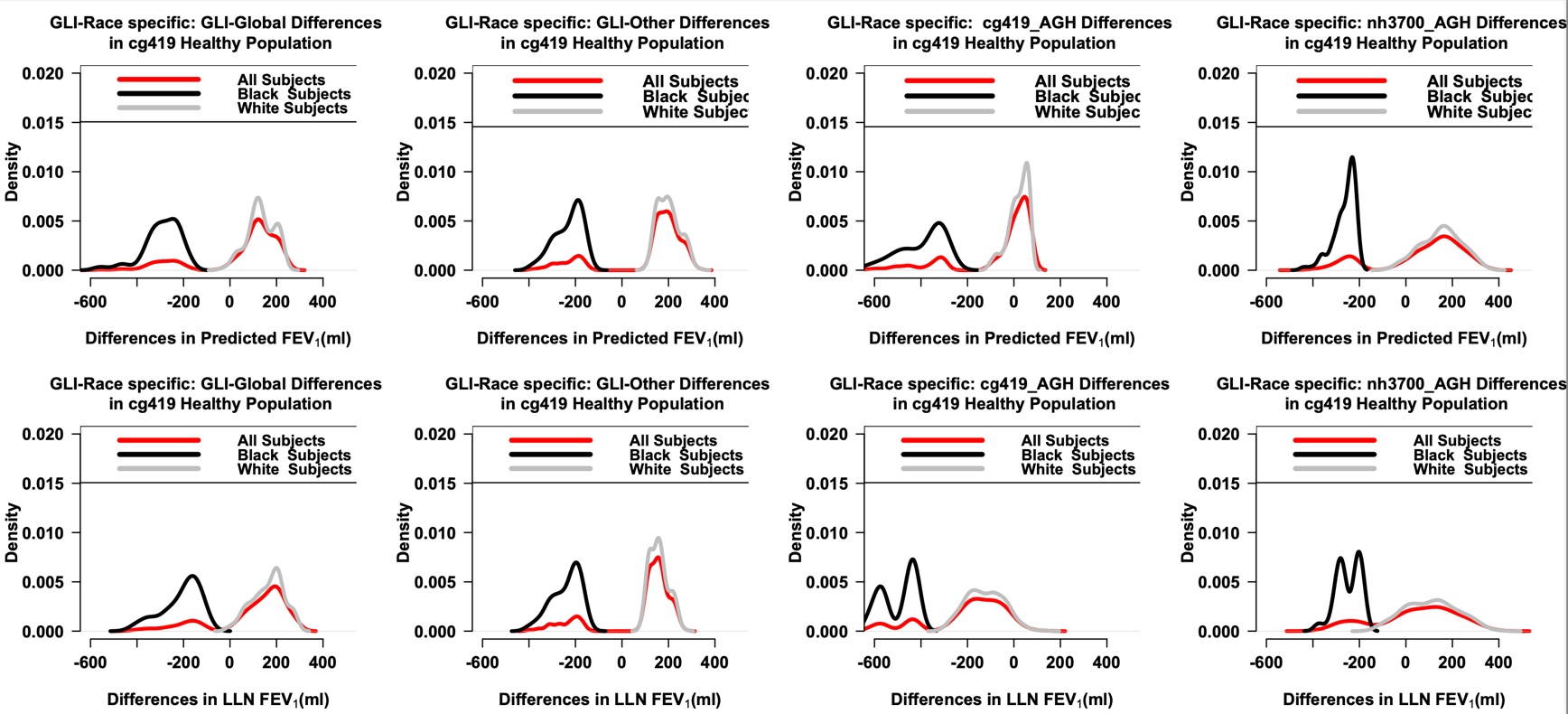


**A.**

**B.**

e-Figure 7. Density plots of the differences between GLI-Race specific estimates of predicted FEV_1_ (ml) or LLN of FEV_1_ (ml) and those derived from race-neutral equations in the nh3700 (A) and cg419 (B) healthy, never smoker cohorts. Differences were calculated by subtracting the predicted FEV_1_ (or LLN) of the stated model from the GLI-Race specific value. In each section, the differences of the predicted FEV_1_ and the LLN of the FEV_1_ values are shown in the upper and lower rows respectively. The black, grey and red lines demonstrate the densities of the Black, White and the total population respectively.

## **e-Figure 7. Density plots of the differences between standard GLI-Race specific predicted FEV_1_ and the LLN values and those derived from race-neutral equations in healthy never-smokers.**

## **e-Figure 8. Density plots of differences of predicted and LLN FEV_1_ (ml) between race-neutral and race-free equations**


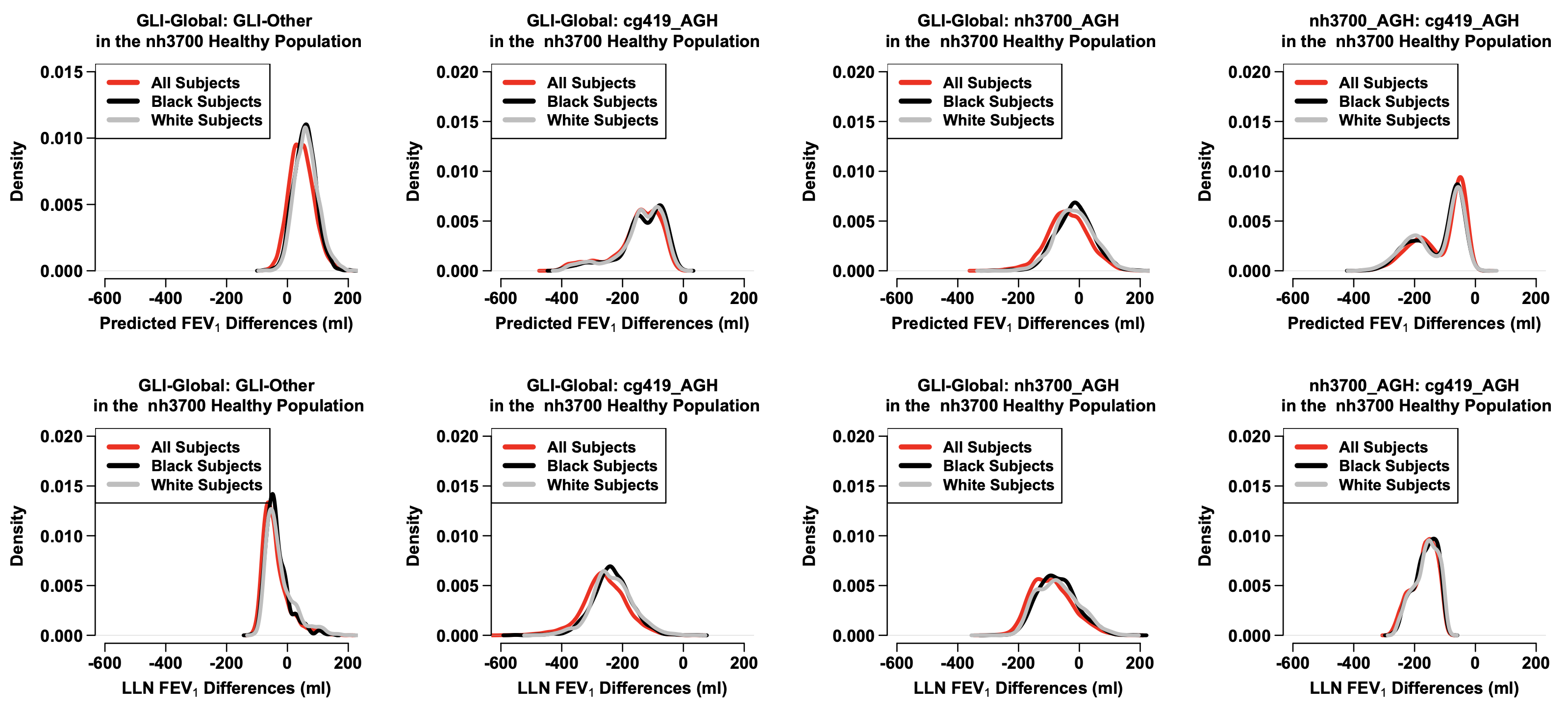


e-Figure 8. Density plots of the differences of the predicted FEV_1_ (or LLN) between the race-neutral GLI-Global values and values derived from GLI-Other and the race-free equations. The figure shows the density plots of the predicted FEV_1_ differences (top row) and the FEV_1_ LLN differences (bottom row) in milliliters. Differences were calculated by subtracting the predicted FEV_1_ (or LLN) of the stated model from the GLI-Global value (columns 1, 2, and 3). The fourth column demonstrates the differences between the two race free models. The black, gray and red lines demonstrate the densities of the Black, White and total populations respectively. Compared to the GLI-Global or GLI-Other values, both race free equations generate predicted FEV_1_ values that are higher (negative differences) and thus would generate lower ppFEV_1_ values. As expected, there were no racial differences with these race-neutral equations.

## **e-Figure 9. Measured FEV_1_ (L) vs ppFEV_1_ from race-specific and race-neutral equation values**


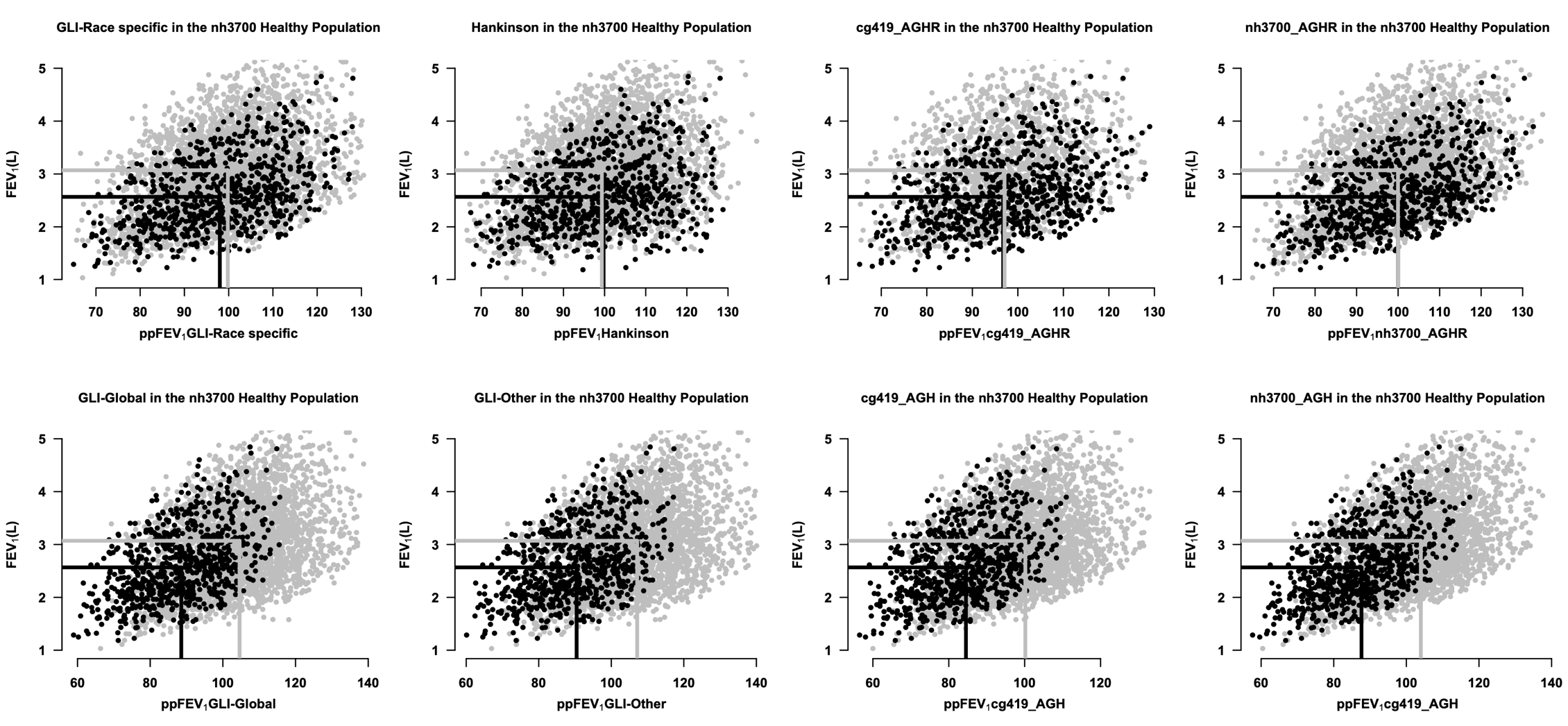

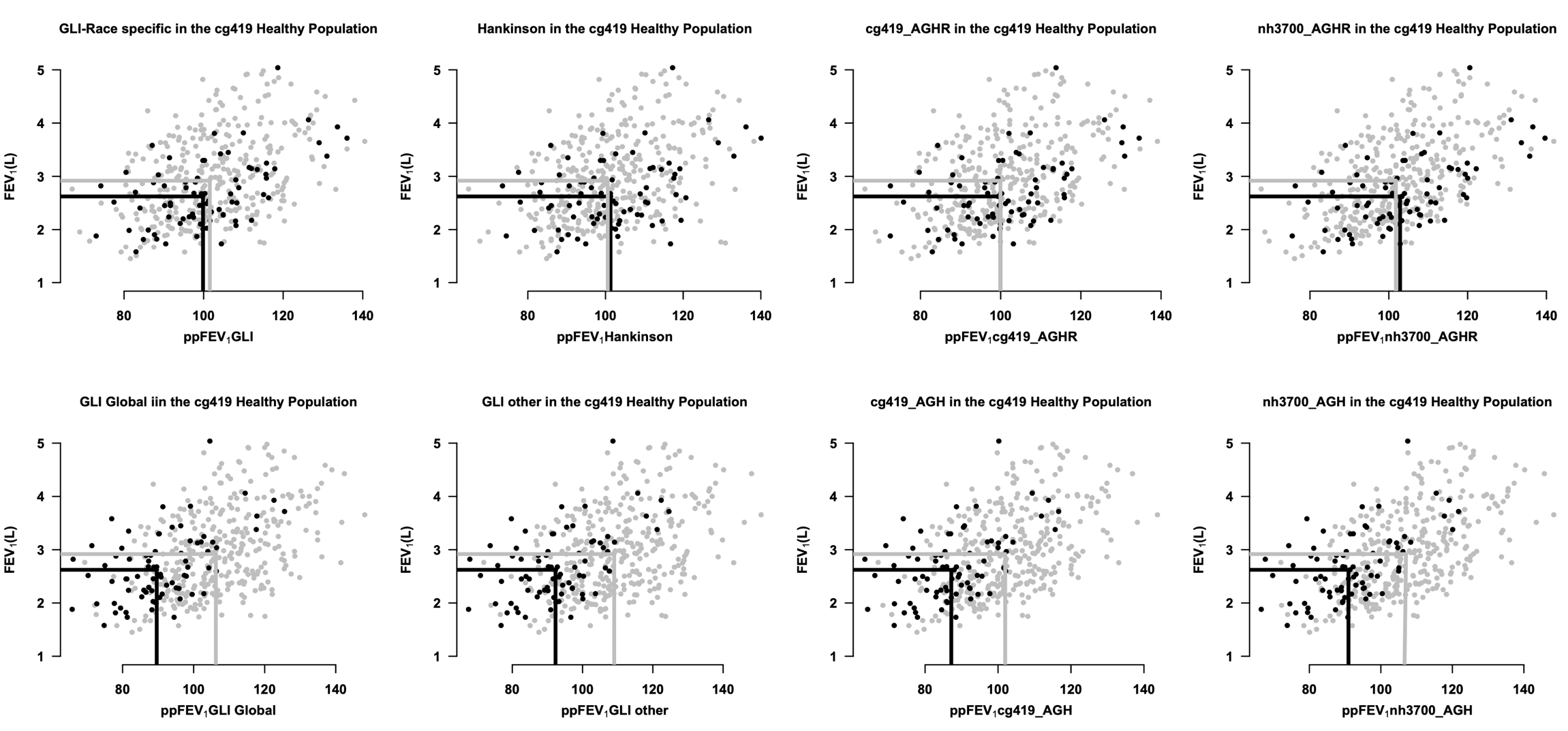


**A.**

**B.**

e-Figure 9. The measured FEV_1_ plotted against ppFEV_1_ derived from race-specific, race-neutral, and race-free spirometry reference equations in the nh3700 (A) and cg419 (B) healthy, never smoker populations. Black and gray points represent the Black and White participants respectively. The horizontal and vertical black lines represent the median values of the measured FEV_1_ and ppFEV_1_ respectively for the Black participants. The horizontal and vertical gray lines represent the median values of the measured FEV_1_ and ppFEV_1_ respectively for the White participants. The overlay of black and grey lines in the plots using race-specific equations demonstrates how the normalization by race obscures the measured differences between Black and White participants in both healthy cohorts. Abbreviations: ppFEV_1_= percent predicted FEV_1_

## **e-Figure 10. The effects of race in FEV_1_ models in the different NHANES race/ethnicity groups**


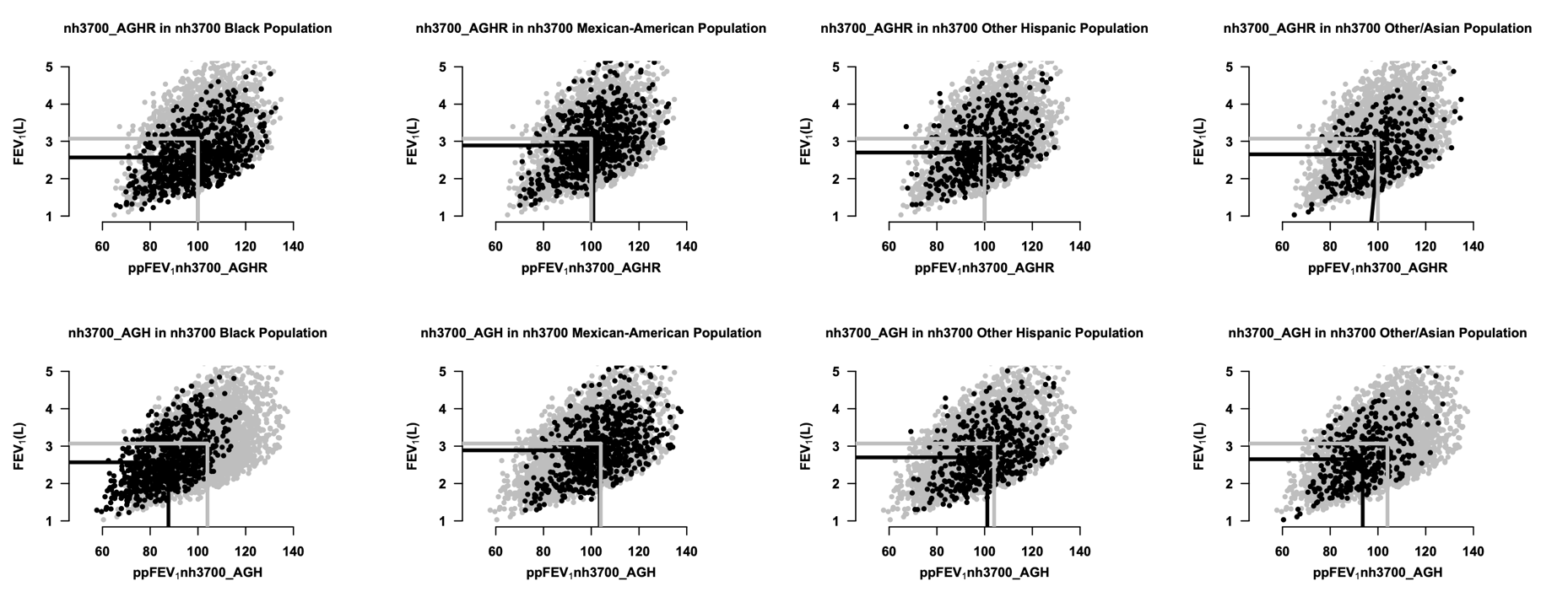


**A.**

**B.**

**C.**

**D.**


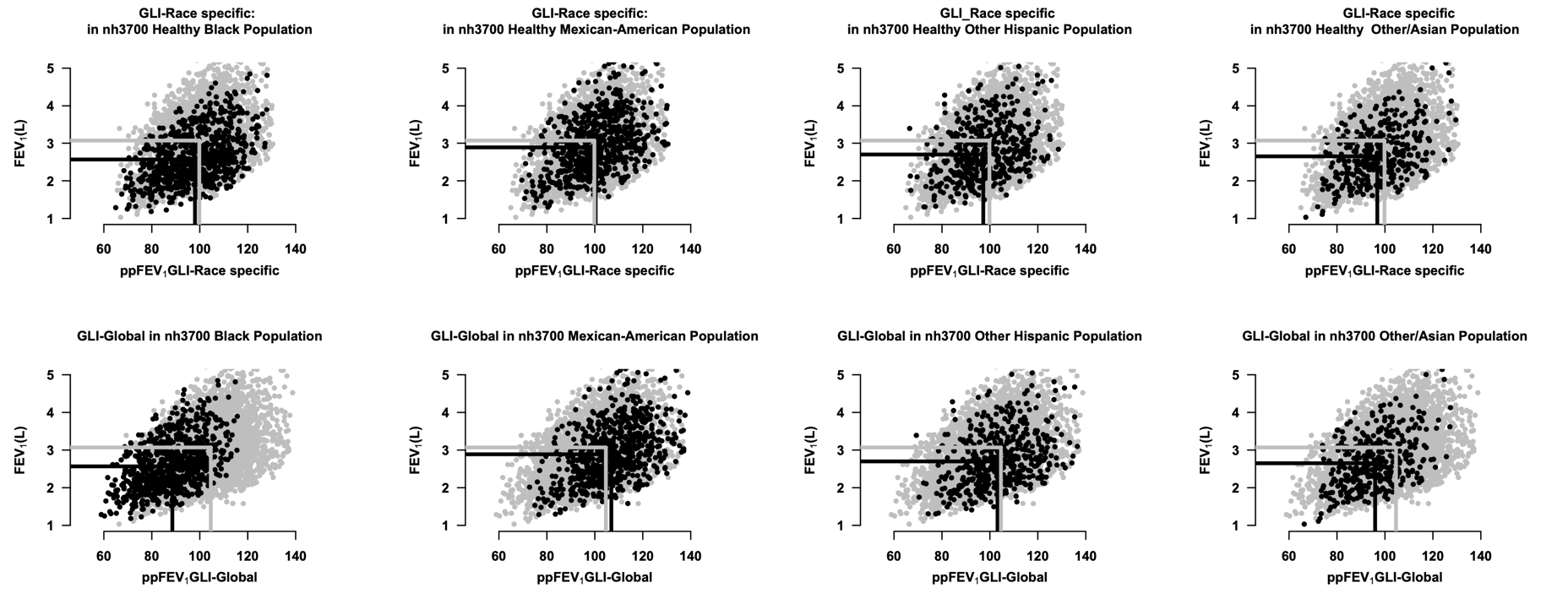


e-Figure 10. The measured FEV_1_ plotted against ppFEV_1_ values derived from the GLI-Race specific (A), GLI-Global (B) , nh3700_AGHR (C), and nh3700_AGH equations (D) in the nh3700 healthy, never-smoker population. The gray points represent the White participants. The black points represent individuals from each different racial/ethnic group: Black individuals (column 1), Mexican-American individuals (column 2), Other Hispanic individuals (column 3), and Other/Asian/mixed race individuals (column 4) in the NHANES cohort. The horizontal and vertical black lines represent the median values of the measured FEV_1_ and ppFEV_1_ respectively for the different racial/ethnicity participants. The horizontal and vertical gray lines represent the median values of the measured FEV_1_ and ppFEV_1_ respectively for the White participants. The largest differences in FEV_1_ values, and the biggest race normalization effects are seen in the Black and Mixed Race/Other/Asian groups relative to the White participants. The racial coefficient is minimally different for Mexican Americans or Other Hispanics relative to White participants.

## **e-Figure 11. The FEV_1_ normalization equations using alternative anthropometric measures in place of height generate similar differences in ppFEV_1_**


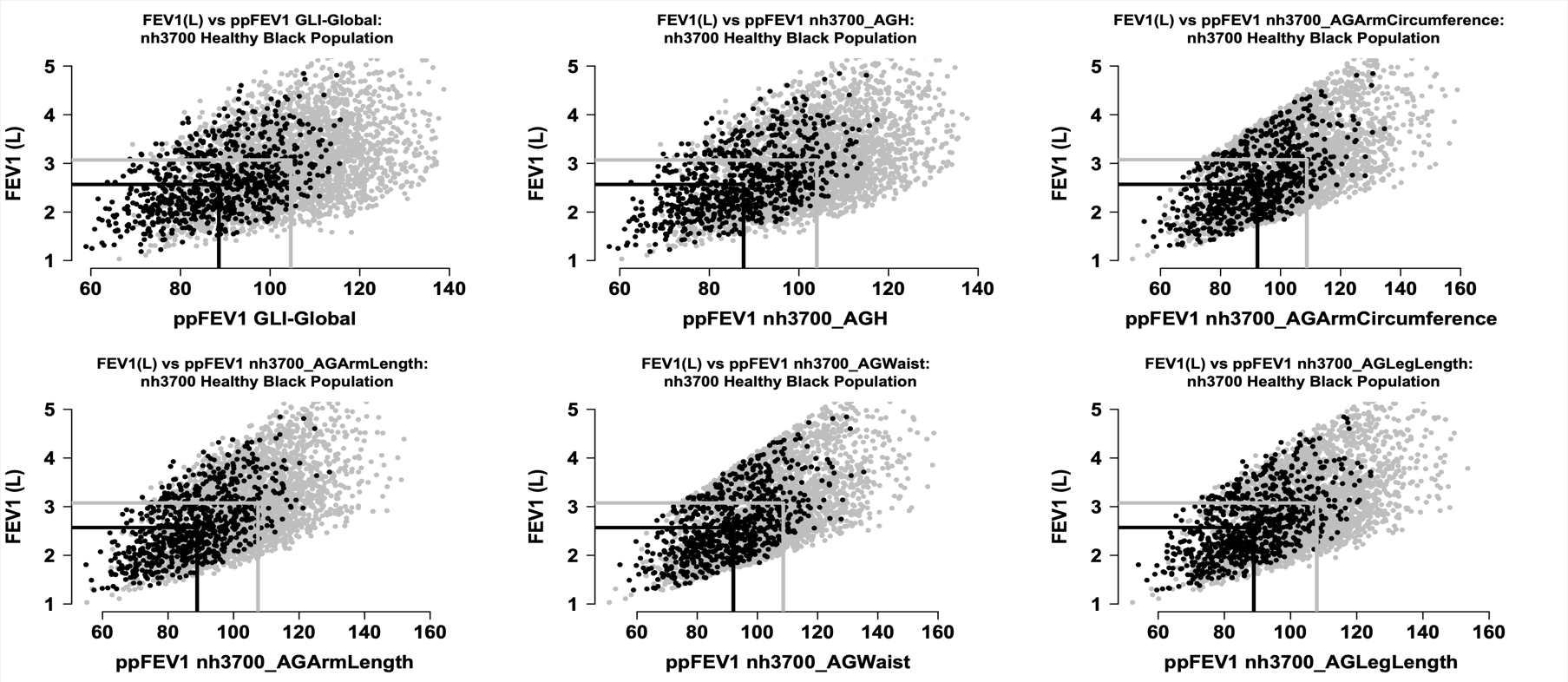

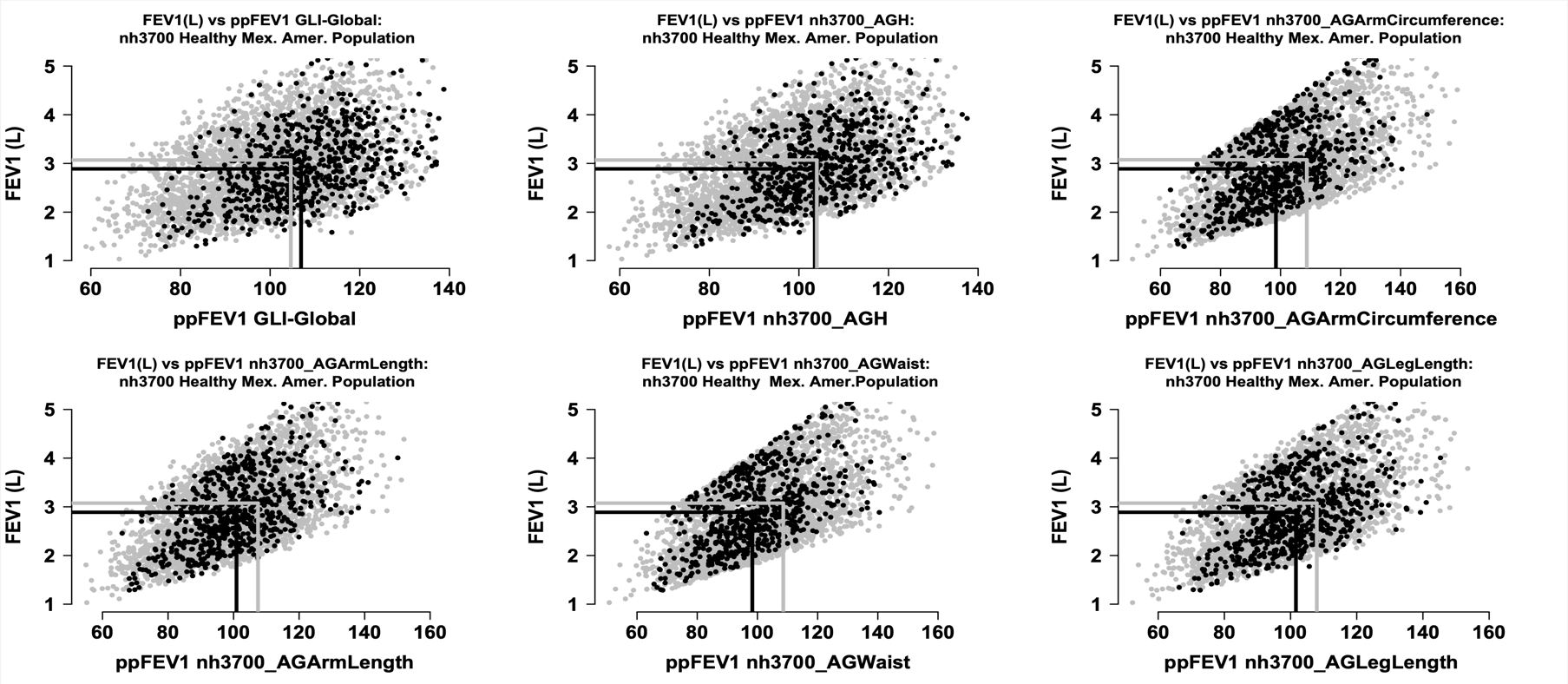

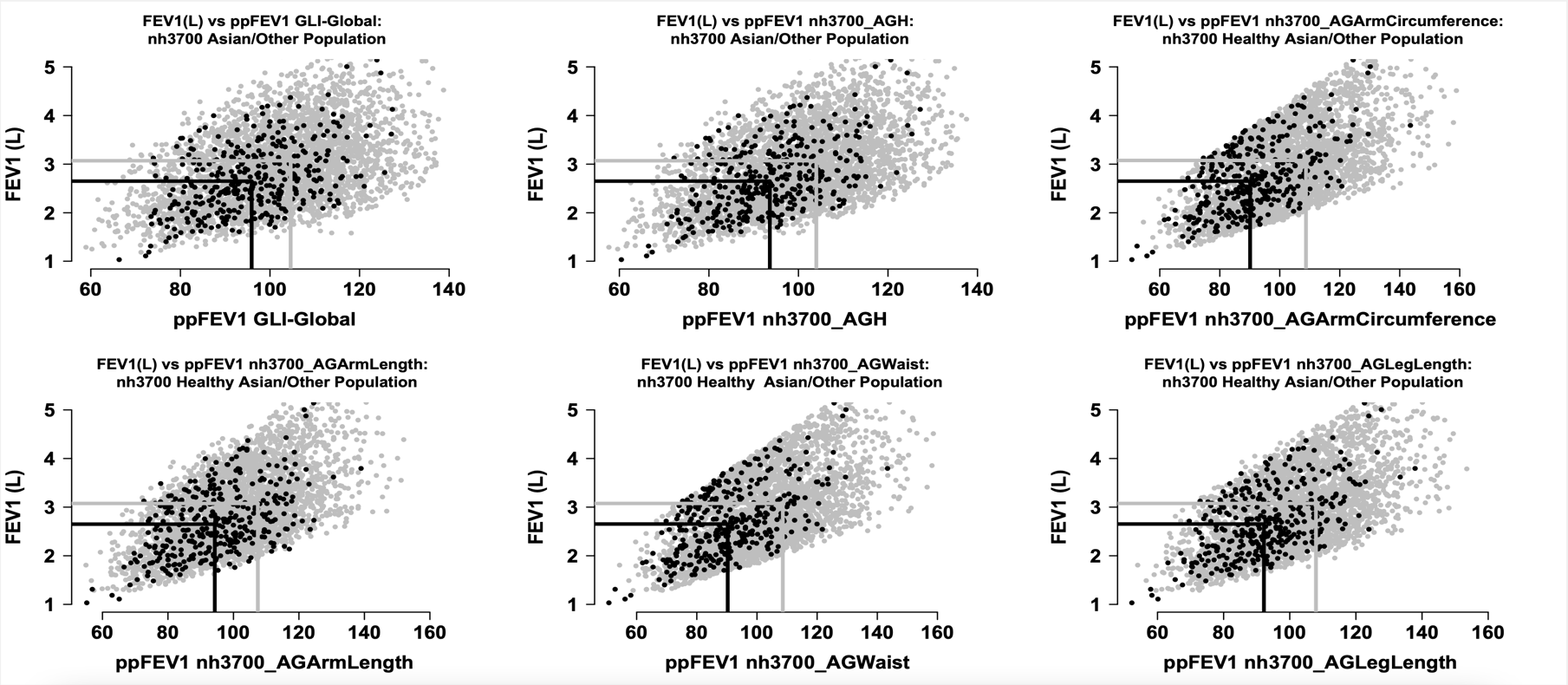


**A.**

**B.**

**C.**


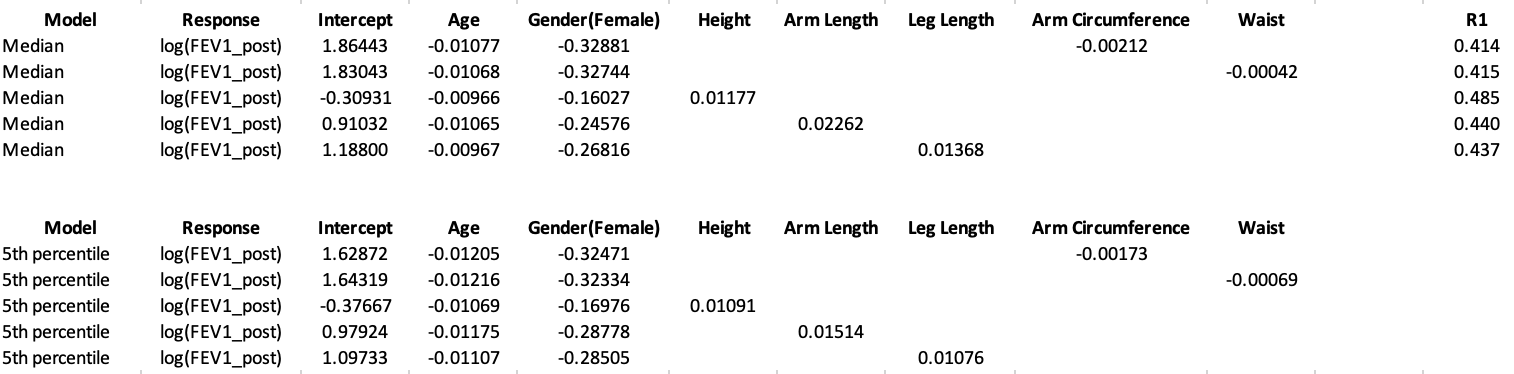


e-Figure 11. Measured FEV_1_ plotted against ppFEV_1_ derived from race-neutral spirometry reference equations using alternative anthropometric measures in place of standing height (e.g. arm circumference, upper arm length, waist, upper leg length) in healthy never smoking Black (A), Mexican-American (B) and Other/Mixed race/Asian (C) populations in NHANES (nh3700) cohort. The horizontal and vertical black lines represent the median values of the measured FEV_1_ and ppFEV_1_ respectively for the indicated participants (black points). The horizontal and vertical gray lines represent the median values of the measured FEV_1_ and ppFEV_1_ respectively for the White participants (gray points). Each graph demonstrates the effects of race-free models shown in the table. D. Tables of coefficients from models built to estimate FEV_1_ (median and 5^th^ quantile/percentile) using as covariates each anthropometric measure separately along with age and gender. Of the available anthropometric measures, standing height generated the best fitting model as assessed by the highest R1 value while the alternative anthropometrics continued to generate predicted FEV_1_ values that preserved the relative differences of the measured FEV_1_ between White participants and the Black and Other/Mixed race/Asian populations.

**D.**

# **Section 4. Effects of New Race-specific and Race-neutral Models on ppFEV_1_ and GOLD Classification in COPDGene and nh785 smokers.**

#
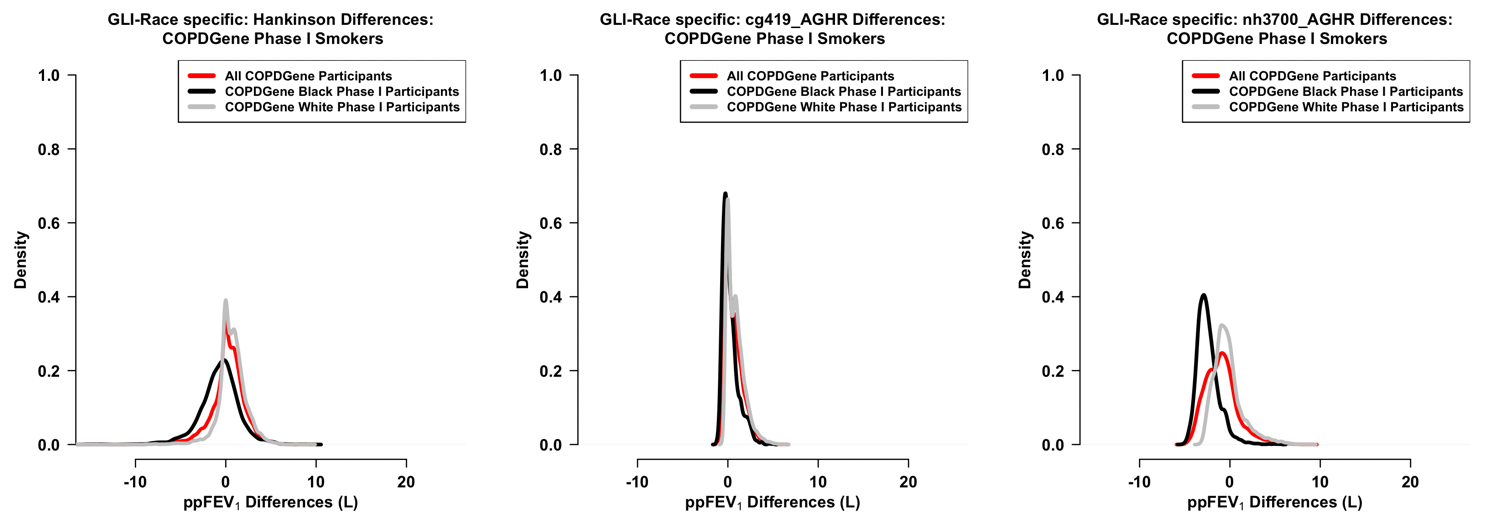


## **e-Figure 12. Density plots of differences between the GLI reference and race-specific equation values for ppFEV_1_ in COPDGene smokers**

#

e-Figure 12. Density plots of the differences between the ppFEV_1_ values derived from the race-specific equations relative to the GLI reference equations, including the Hankinson equation and the race-specific equations generated from the healthy never smokers in COPDGene (ppFEV_1_ cg419_AGHR) and NHANES datasets (ppFEV_1_ nh3700_AGHR). Differences were calculated by subtracting the ppFEV_1_ (derived from the stated model from the GLI-Race specific value). The race-specific equations show close agreement i.e. within 3% of the GLI standard in the Black and White smoking participants of the COPDGene study. The black, grey and red lines demonstrate the densities of the Black, White and total populations respectively.

## **e-Figure 13. Density plots of differences between the GLI-Global race-neutral and the race-free ppFEV_1_ values in smoking cohorts**


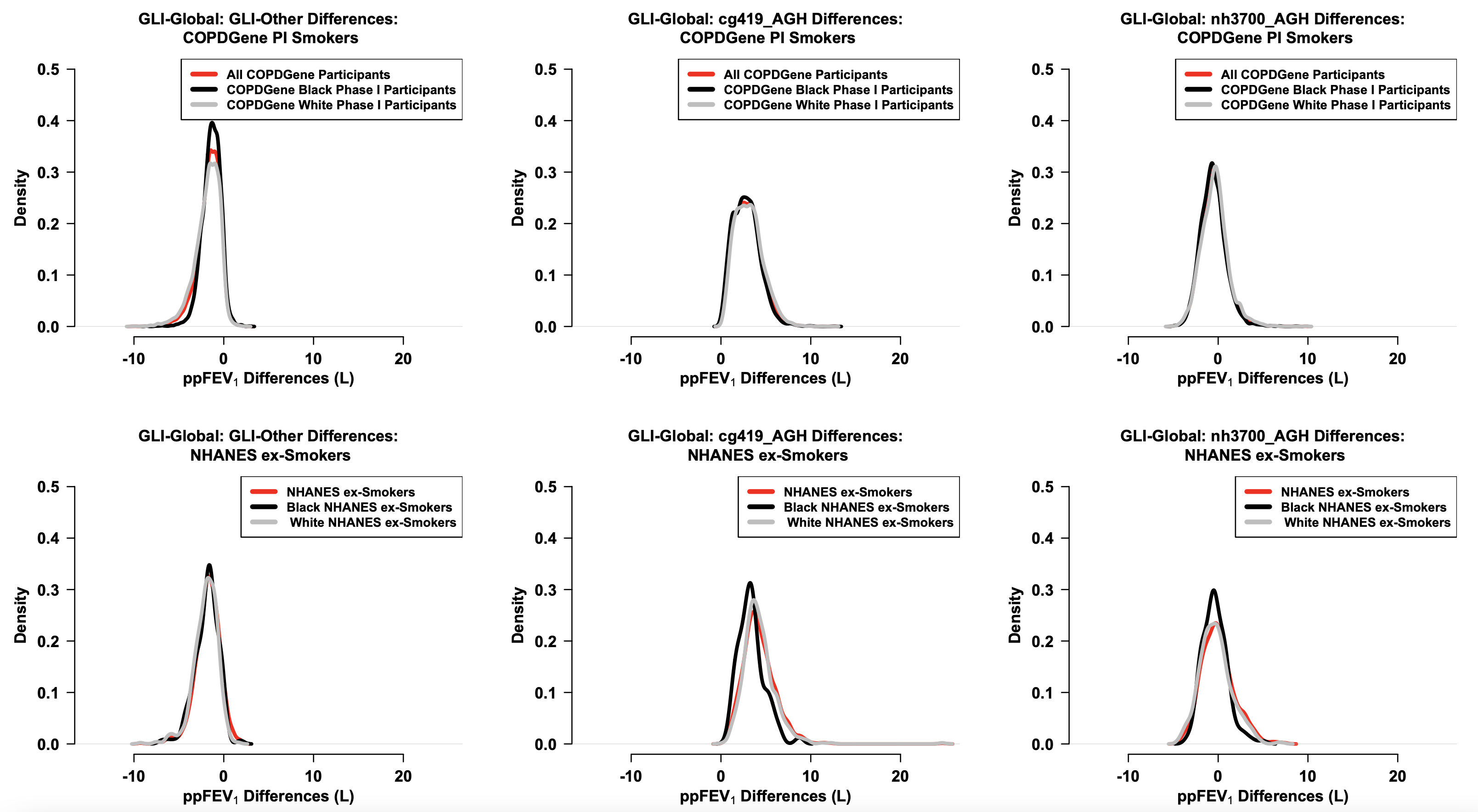


e-Figure 13. Density plots of Differences between the GLI-Global, GLI-Other and the race-free ppFEV_1_ values in COPDGene Smokers. Differences were calculated by subtracting the ppFEV_1_ (of the stated model) from the GLI-Global value. Relative to the GLI-Global values, the race-free equations generate higher predicted FEV_1_ and thus lower ppFEV_1_ values. As expected, there were no racial differences using these race neutral equations.

# **Section 5. Quantitative Chest CT Phenotype and Dyspnea Models in Smokers**

## **e-Figure 14. Race-specific sensitivity analysis of quantitative chest CT phenotypes**

**A.**

**B.**


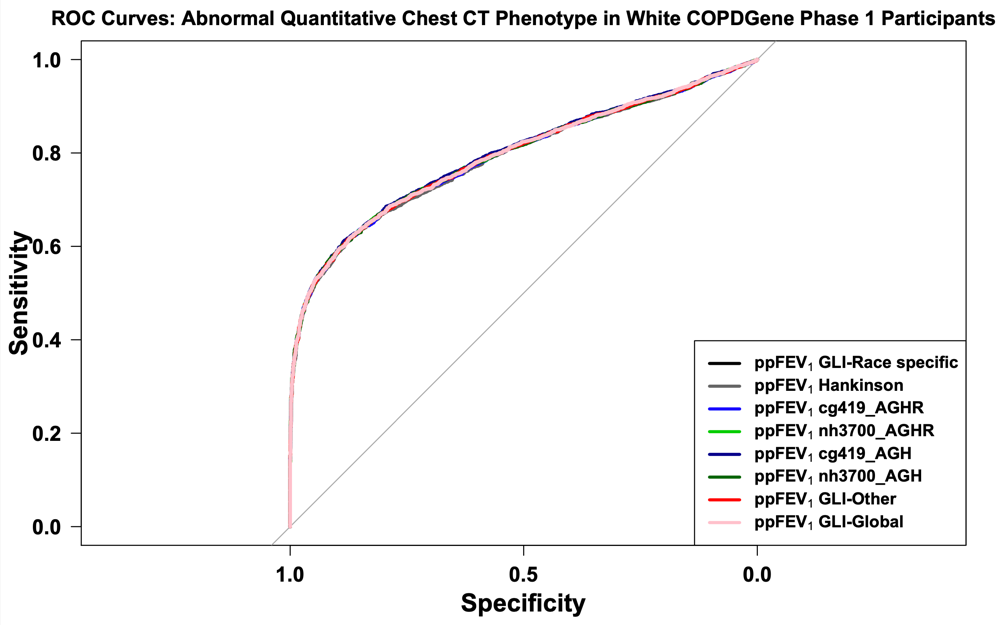

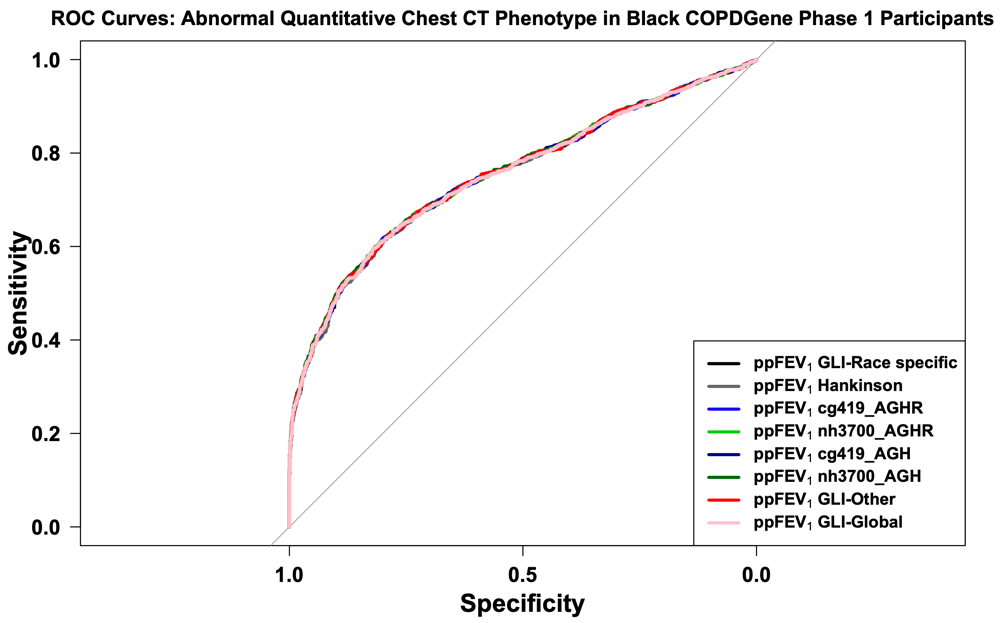


**C.**

**D.**

**E.**


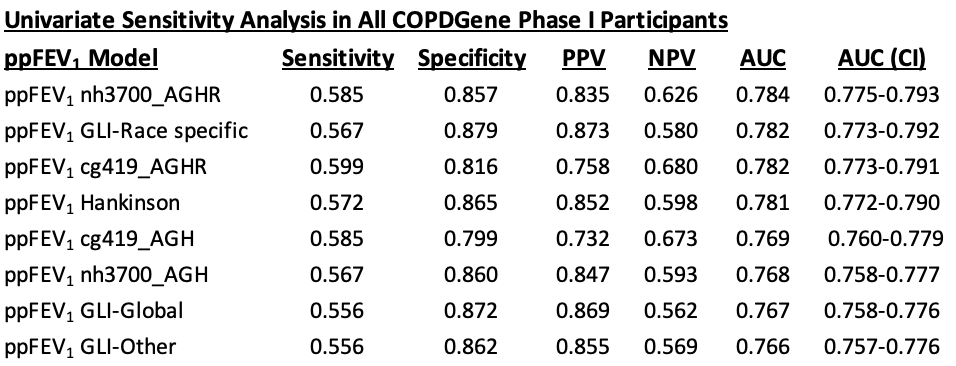

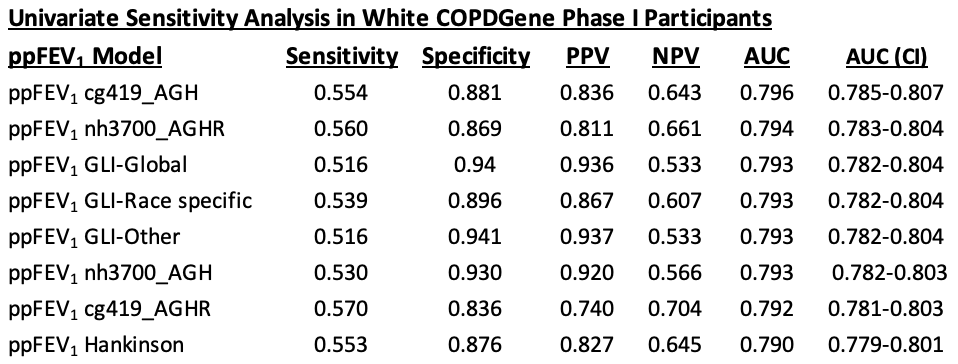

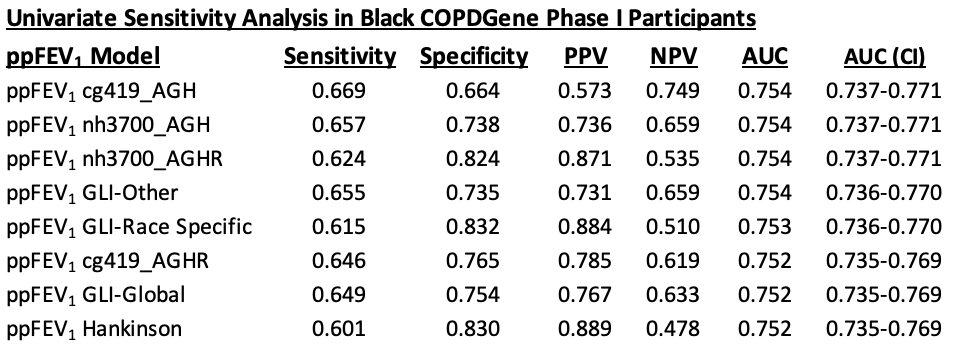


e-Figure 14. Race-specific univariable analysis of quantitative Chest CT phenotypes. ROC curves of any abnormal quantitative chest CT phenotypes, i.e. 1) percent emphysema > 5%, 2) percent air trapping > 15%, 3) airway wall thickness if the Pi10 > 2.5 mm) in Black (A) and White (B) individuals from the COPDGene smoking participants as predicted by the ppFEV_1_. Each colored line corresponds to a ROC curve using ppFEV1 values derived from the listed race-specific and race-neutral model equations. Sensitivity, specificity, positive predicted value (PPV), negative predictive value (NPV), area under the ROC curve, and the AUC confidence intervals (CI) of the listed abnormal chest CT phenotype models of in the White individuals (C), Black individuals (D) or Whole population (E) from COPDGene Phase 1 cohort. The sensitivity and specificity analysis used the LLN or the 5^th^ percentile for each of the models.

**e-Table 4. Race-specific mulitvariable logistic regression and Random Forest models of quantitative chest CT phenotypes.**

**Syntax: glm(abnl CT phenotype~ ppFEV_1_ GLI+ scanner make + smoking status + FEV_1_/FVC ratio + pack-years + gender(female), binomial)**

##

1. **Representative Multivariable Logistic Regression Model of Abnormal CT Findings)**


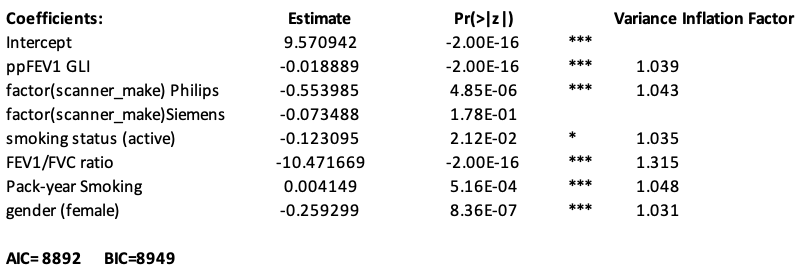


**B) Race specific AIC/BIC charts**


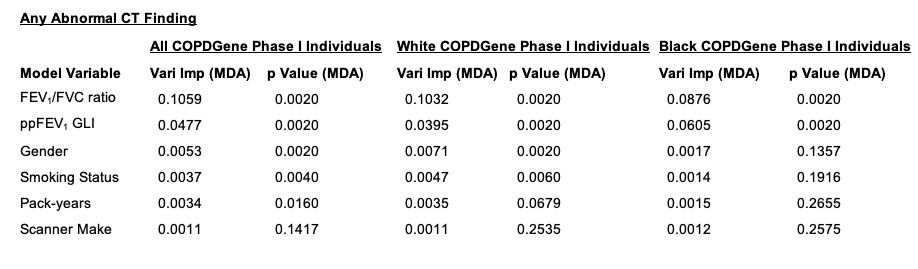

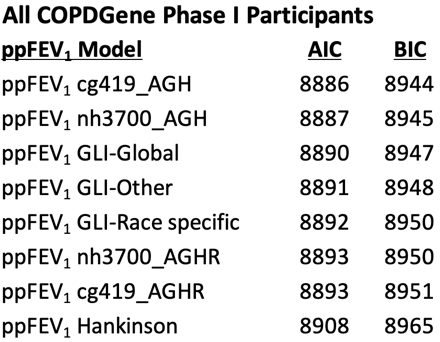

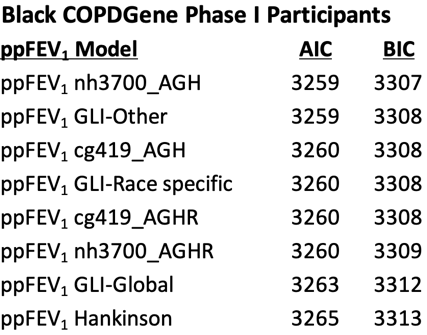

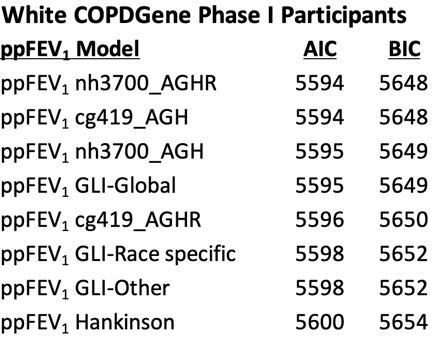


**C) Representative Random Forest Model of Abnormal CT Findings**

**rfPermute(Response~ ppFEV_1_ GLI+ scanner make + smoking status + FEV_1_/FVC ratio + pack-years + gender, ntree=100, nrep=100)**


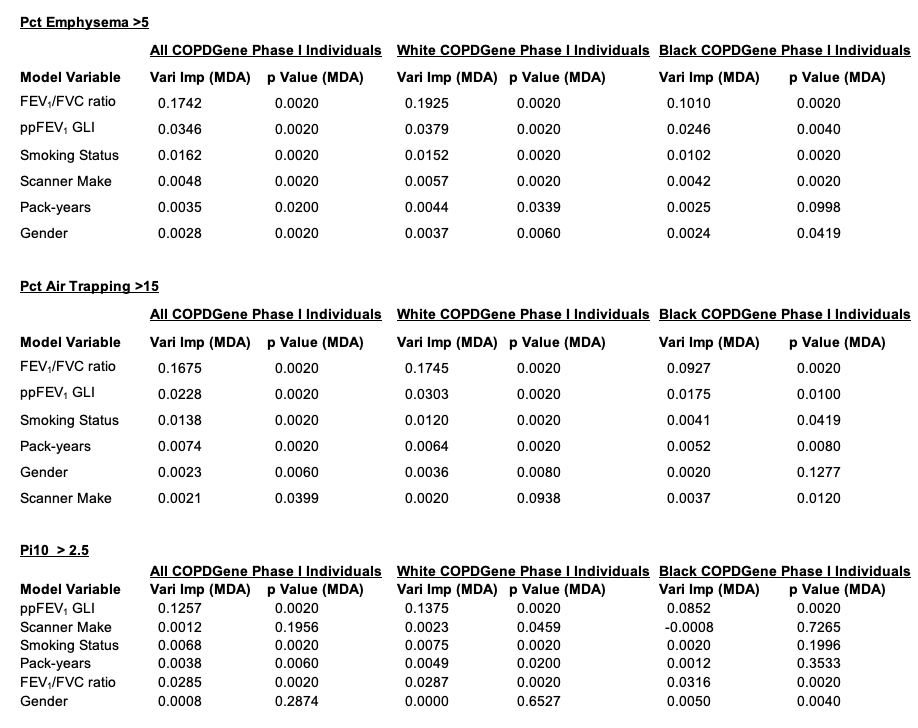


**D. Random Forests Classification Error Rates and Confidence Intervals (CI)**

| **Emphysema > 5%** | |  | | | | | |
| --- | --- | --- | --- | --- | --- | --- | --- |
| **Variable** | | **All (CI)** | | **White (CI)** | | **Black (CI)** | |
| ppFEV1 nh3700_AGH | 15.3 (14.6-16.0) | | 17.6 (16.9-18.8) | | 10.6 (9.5-11.7) | |  |
| ppFEV1 nh3700_AGHR | 15.4 (14.6-16.1) | | 17.6 (16.7-18.6) | | 10.7 (9.60-11.8) | |  |
| ppFEV1 cg419_AGH | 15.4 (14.6-16.1) | | 17.8 (16.6-18.5) | | 10.6 (9.5-11.7) | |  |
| ppFEV1 GLI-Race specific | 15.4 (14.7-16.1) | | 17.9 (16.9-18.8) | | 10.8 (9.70-12.0) | |  |
| ppFEV1 cg419_AGHR | 15.4 (14.7-16.2) | | 17.6 (16.7-18.6) | | 10.6 (9.50-11.7) | |  |
| ppFEV1 GLI-Global | 15.4 (14.7-16.2) | | 18.0 (17.0-18.9) | | 10.8 (9.7-11.9) | |  |
| ppFEV1 Hankinson | 15.5 (14.7-16.2) | | 17.8 (16.9-18.8) | | 10.8 (9.70-12.1) | |  |
| ppFEV1 GLI-Other | 15.6 (14.9-16.3) | | 17.9 (16.9-18.8) | | 10.8 (9.8-12.0) | |  |
|  |  | |  | |  | |  |
| **AirTrapping > 15%** |  | |  | |  | |  |
| **Variable** | **All (CI)** | | **White (CI)** | | **Black (CI)** | |  |
| ppFEV1 nh3700_AGHR | 21.1 (20.3-22.0) | | 22.3 (21.2-23.3) | | 19.5 (18.1-20.9) | |  |
| ppFEV1 GLI-Global | 21.2 (20.3-22.0) | | 22.5 (21.5-23.6) | | 19.6 (18.2-21.0) | |  |
| ppFEV1 GLI-Other | 21.2 (20.4-22.0) | | 22.5 (21.5-23.5) | | 19.6 (18.2-21.0) | |  |
| ppFEV1 nh3700_AGH | 21.2 (20.4-22.1) | | 22.5 (21.5-23.6) | | 19.5 (18.2-21.0) | |  |
| ppFEV1 GLI-Race specific | 21.3 (20.4-22.1) | | 22.5 (21.5-23.5) | | 19.5 (18.1-21.0) | |  |
| ppFEV1 cg419_AGH | 21.3 (20.4-22.1) | | 22.3 (21.3-23.4) | | 19.6 (18.2-21.0) | |  |
| ppFEV1 Hankinson | 21.3 (20.5-22.2) | | 22.4 (21.4-23.5) | | 19.7 (18.4-21.2) | |  |
| ppFEV1 cg419_AGHR | 21.4 (20.6-22.2) | | 22.6 (21.6-23.6) | | 19.6 (18.2-21.0) | |  |
|  |  | |  | |  | |  |
| **Pi10 >2.5mm** |  | |  | |  | |  |
| **Variable** | **All (CI)** | | **White (CI)** | | **Black (CI)** | |  |
| ppFEV1 GLI-Other | 23.4 (22.6-24.3) | | 22.0 (20.7-22.8) | | 26.7(25.2-28.3) | |  |
| ppFEV1 GLI-Global | 23.6 (22.7-24.4) | | 22.2 (21.2-23.3) | | 27.0 (25.5-28.7) | |  |
| ppFEV1 cg419_AGH | 23.6 (22.8-24.5) | | 22.0 (21.0-23.0) | | 26.7 (25.2-28.3) | |  |
| ppFEV1 nh3700_AGH | 23.7 (22.6-24.3) | | 22.0 (21.5-23.6) | | 26.7 (25.2-28.3) | |  |
| ppFEV1 GLI-Race specific | 23.8 (22.9-24.6) | | 21.8 (20.7-22.8) | | 26.2 (24.7-27.8) | |  |
| ppFEV1 cg419_AGHR | 23.8 (23.0-24.7) | | 21.9 (20.9-23.0) | | 26.5 (25.0- 28.1) | |  |
| ppFEV1 nh3700_AGHR | 23.8 (23.0-24.7) | | 22.0 (21.0-23.0) | | 26.3 (24.7-27.9) | |  |
| ppFEV1 Hankinson | 24.1 (23.0-25.0) | | 22.2 (21.2-23.2) | | 26.9 (25.3- 28.5) | |  |

e-Table 4. Multivariable Logistic Regression and Random Forest models of Quantitative Chest CT Phenotypes. A) Multivariable logistic regression models were generated initially using nine variables with known associations with the quantitative chest CT metrics and included the ppFEV1, the scanner make, smoking status (active or not), the FEV1_1_/FVC ratio, smoking history in pack-years, gender, height, age and BMI. Using the syntax shown in (A), the glm model was simplified with the step function eliminating BMI, height and age. The variance inflation factors for all remaining variables were < 1.5. The table in E5A shows estimated coefficients and p-values; (*** indicates p<0.0001, and * indicates p<0.05) for the representative model with the ppFEV_1_ GLI-Race specific values. B) AIC and BIC values comparing models based on different reference equations, and C) Random Forest Model Variable Importance (Vari Imp MDA) and p values (MDA). The *randomForest* (v4.6-14) and *rfPermute* (v2.1.81) packages were used to obtain the classification error rates, mean decrease in accuracy (MDA), and p-values. The default setting was used for mtry. The ntree and nrep parameters were set at 100 each. Increasing these values lowered the p values but did not change the classification error rates. These studies demonstrated that the models of quantitative chest CT did not improve with the race-specific equations. Race-specific classification error rates and confidence intervals (CI) are shown for each of the quantitative chest CT phenotypes (D).

## **e-Figure 15. Race specific performance of race-specific and race-neutral equation derived ppFEV_1_ values ability to identify abnormal mMRC values using three distinct modeling approaches.**


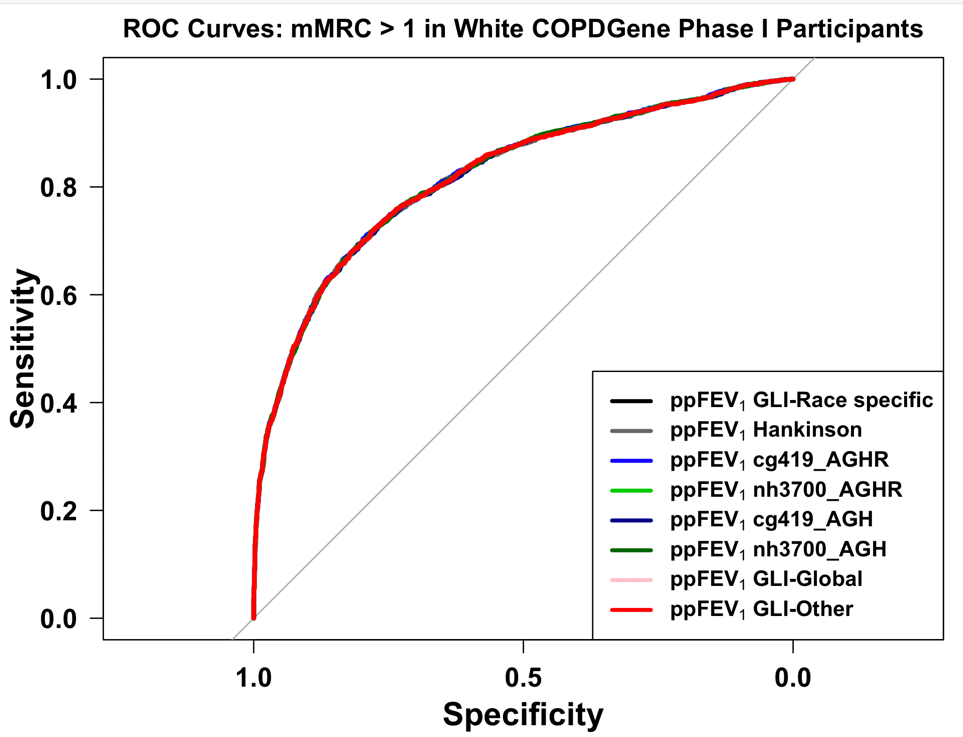

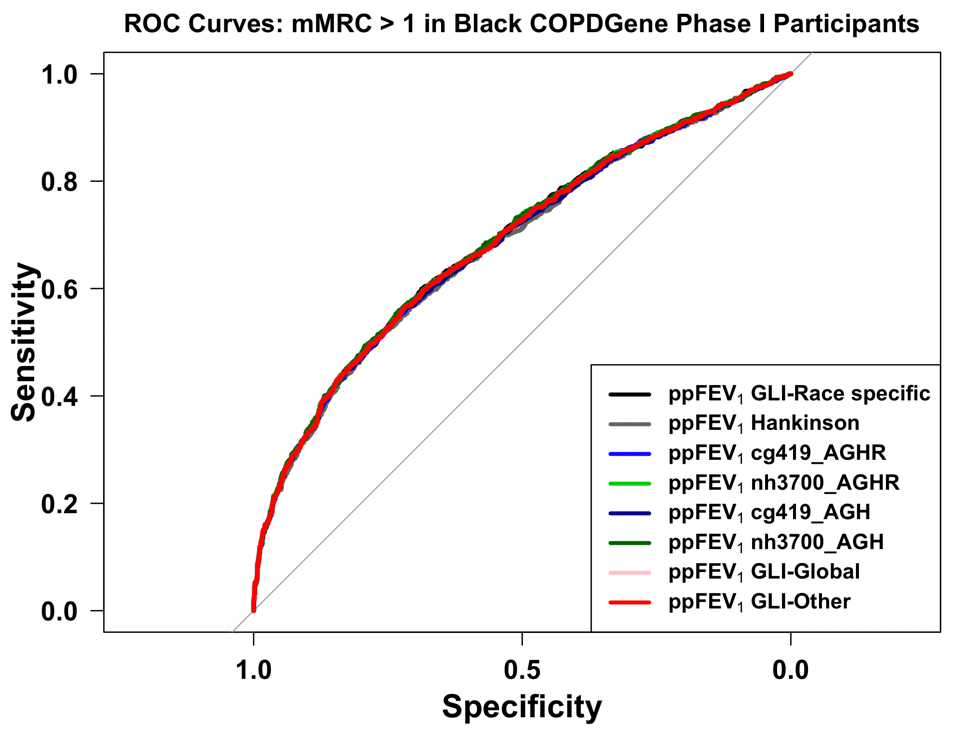

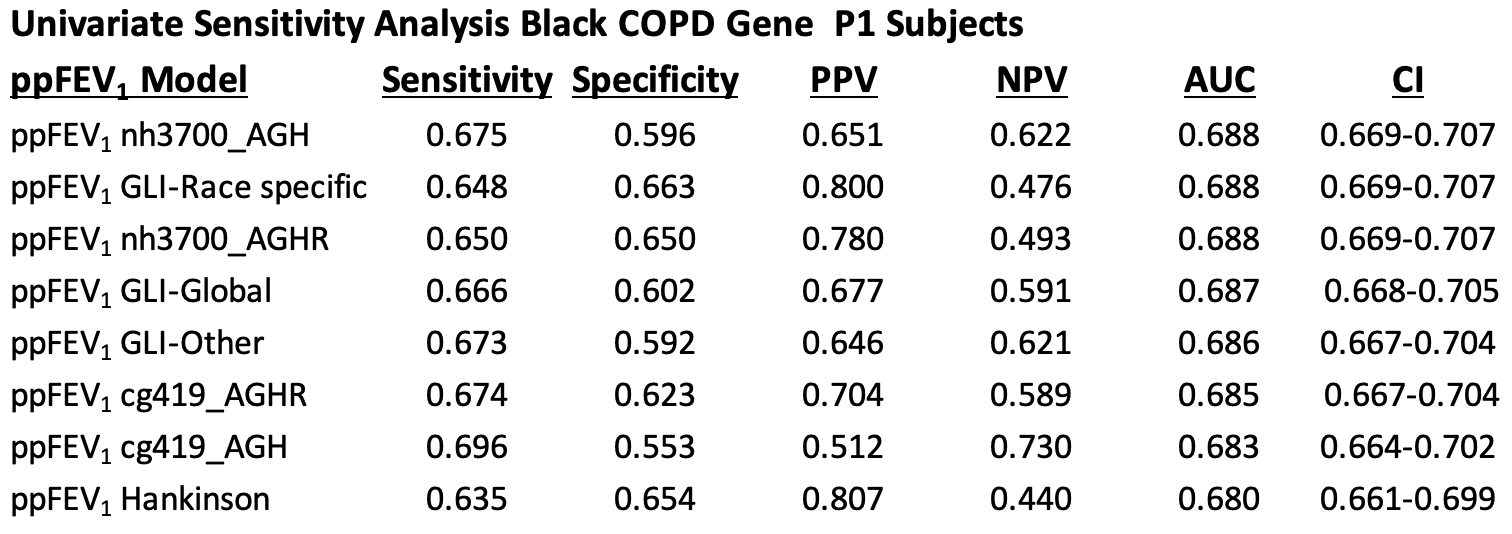

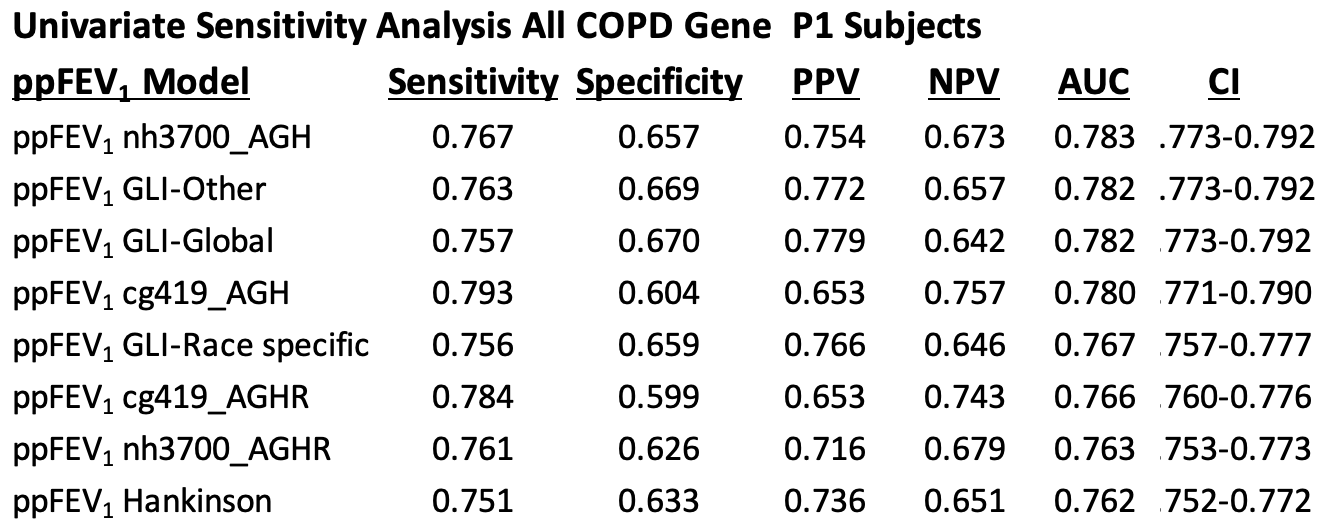

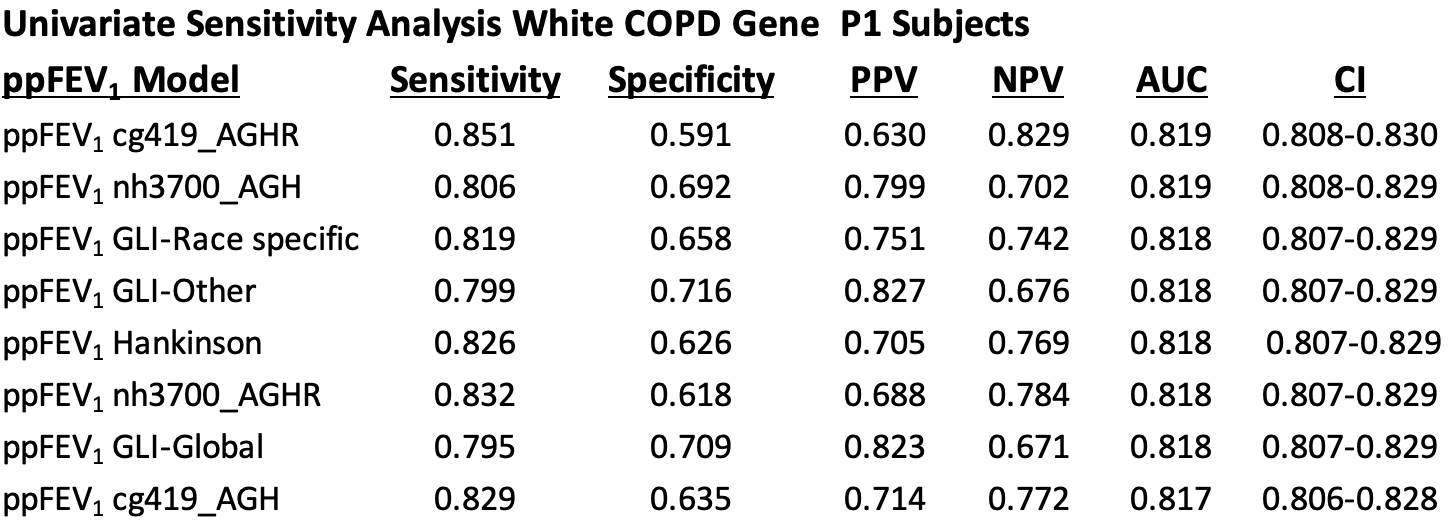


**C.**

**E.**

**B.**

e-Figure 15. Race-specific sensitivity explanatory model of dyspnea (i.e. mMRC > 1) using different race-specific and race neutral values for ppFEV_1_. ROC curves of White (A) and Black (B) COPDGene Phase I participants with a mMRC score of > 1. Each colored line corresponds to a ROC curve using different predFEV_1_ models for the calculation of ppFEV_1_. Sensitivity, specificity, positive predicted value (PPV), negative predicted value (NPV), area under the ROC curve and its confidence interval of the ppFEV_1_ to model a mMRC value of > 1 in all individuals (C), the White individuals (D) or Black individuals (E) from COPDGene Phase I cohort. The sensitivity and specificity analysis used the LLN or the 5^th^ percentile for each of the models. There are no differences in sensitivity/specificity between race-neutral and race-specific equations to identify subjects with mMRC values > 1. In general, spirometry has better sensitivity/specificity to identify an abnormal mMRC score in white than black subjects.

**A.**

**D.**

## **e-Table 5. Race-specific multivariable logistic regression analysis of dyspnea (MMRC > 1)**

1. **Representative Multivariable Logistic Regression Model of mMRC > 1**

**glm(mMRC > 1 ~ ppFEV_1_ GLI+ 6MWD+ bronchodilator response + Age +BMI + FEV_1_/FVC ratio + gender(female) + FEF25-75 + packyears ), binomial)**


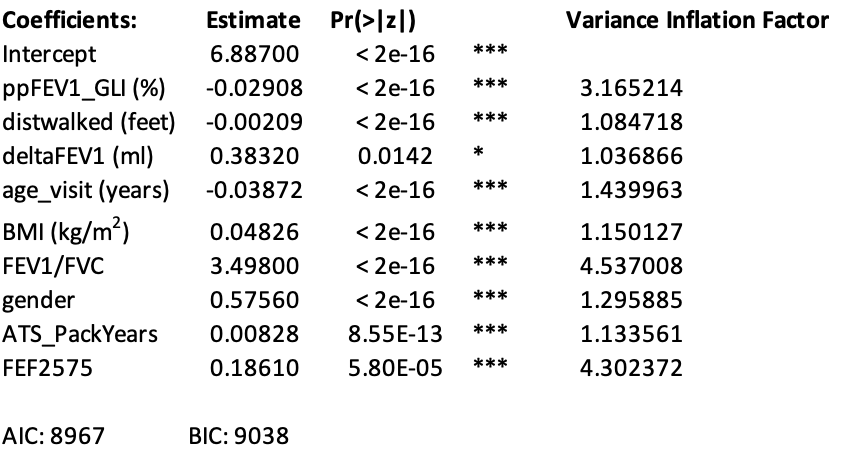


**B. Race specific AIC/BIC charts**


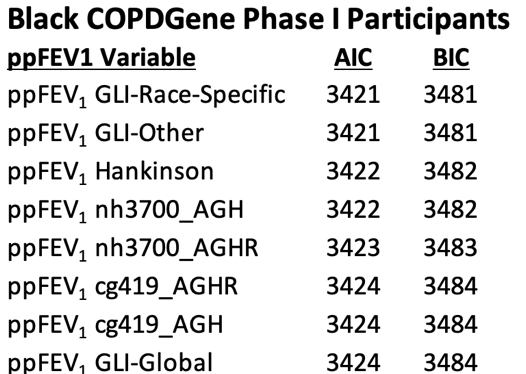

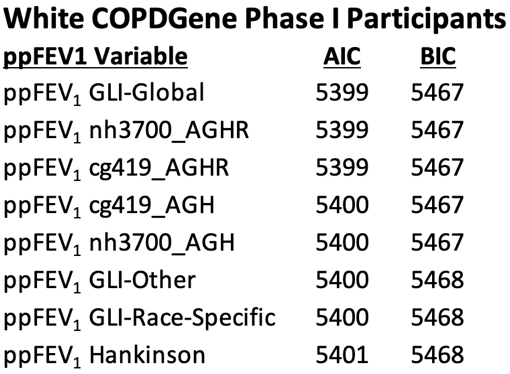

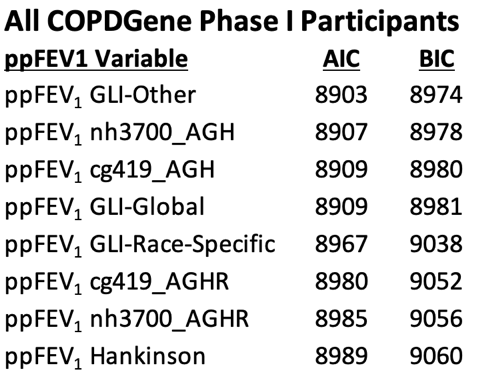


**C. Representative Random Forest Model of mMRC > 1. Syntax: rfPermute (mMRC > 1 ~ ppFEV_1_ GLI+ 6MWD+ bronchodilator response + Age + BMI + FEV_1_/FVC ratio + gender(female) + FEF25-75 + packyears, ntree=100, nrep=100)**


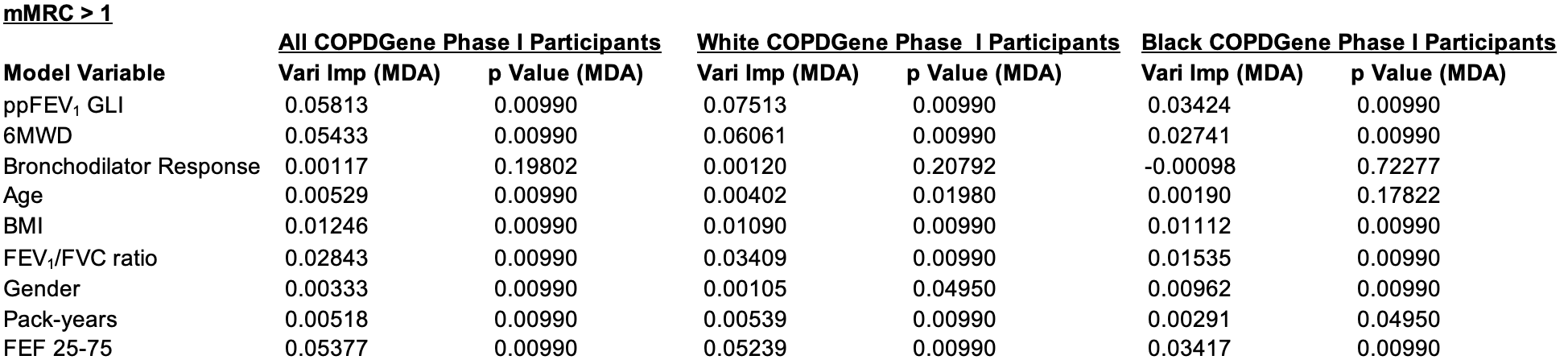


**
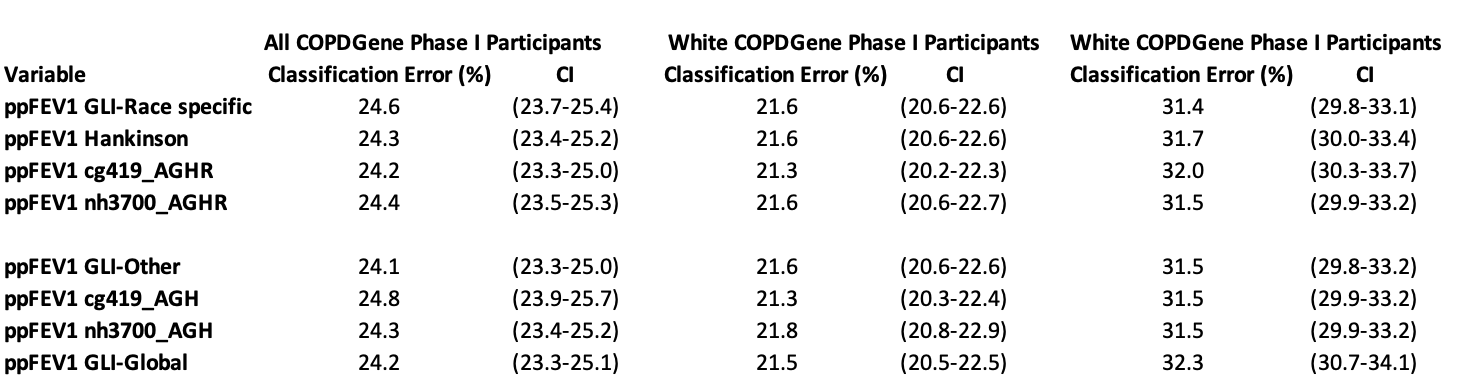
**

e-Table 5. Multivariable logistic regression and Random Forests Models of dyspnea i.e. mMMRC > 1. A) Multivariable logistic regression models were initially generated using the following variables associated with dyspnea: ppFEV_1_, FEV_1/_FVC ratio, pack-year smoking history, age, weight, height, BMI, six minute walking distance (6MWD), bronchodilator response (ml), current smoking status (active/inactive), total lung capacity, gender, and FEF 25-75. Using the step function and building the model up from ppFEV_1,_ variables that were most statistically significant, had the greatest effect in decreasing the AIC and had low variance inflation factors, were selected for the final model. Using this approach, the final model used: ppFEV_1_, FEV_1/_FVC ratio, pack-year smoking history, age, BMI, six minute walking distance (6MWD), bronchodilator response (ml), gender, and FEF 25-75. The variance inflation factors for the remaining variables were < 5. The table in E6A shows estimated coefficients and p-values; (*** indicates p<0.0001, and * indicates p<0.05) for the representative model with the ppFEV1GLI-Race specific values.. B) AIC and BIC values comparing models based on different reference equations, and C) Random Forest Model Variable Importance (Vari Imp MDA) and p values (MDA). The *randomForest* (v4.6-14) and *rfPermute* (v2.1.81) packages were used to obtain the classification error rates, mean decrease in accuracy (MDA), and p-values. The default setting was used for mtry. Although p values changed classification error rates did not change by increasing ntree and nrep to > 100 each. These studies show no advantage to using race-specific equations to predict quantitative chest CT abnormalities. Race-specific classification error rates and their confidence intervals are shown (D) The AIC/BIC values of the race-neutral models were lower in the multivariable logistic regression model suggesting a better fit but the clinical significance of these differences is unclear and likely represents differences of these models to predict dyspnea between the races. In no situation was there an advantage to using race-specific equations to predict dyspnea.

**D. Race-specific classification error rates and their confidence intervals are shown**

e-Figure 16. Comparisons of race-neutral GLI-Global and race free nh3700_AGH models in two distinct healthy populations.


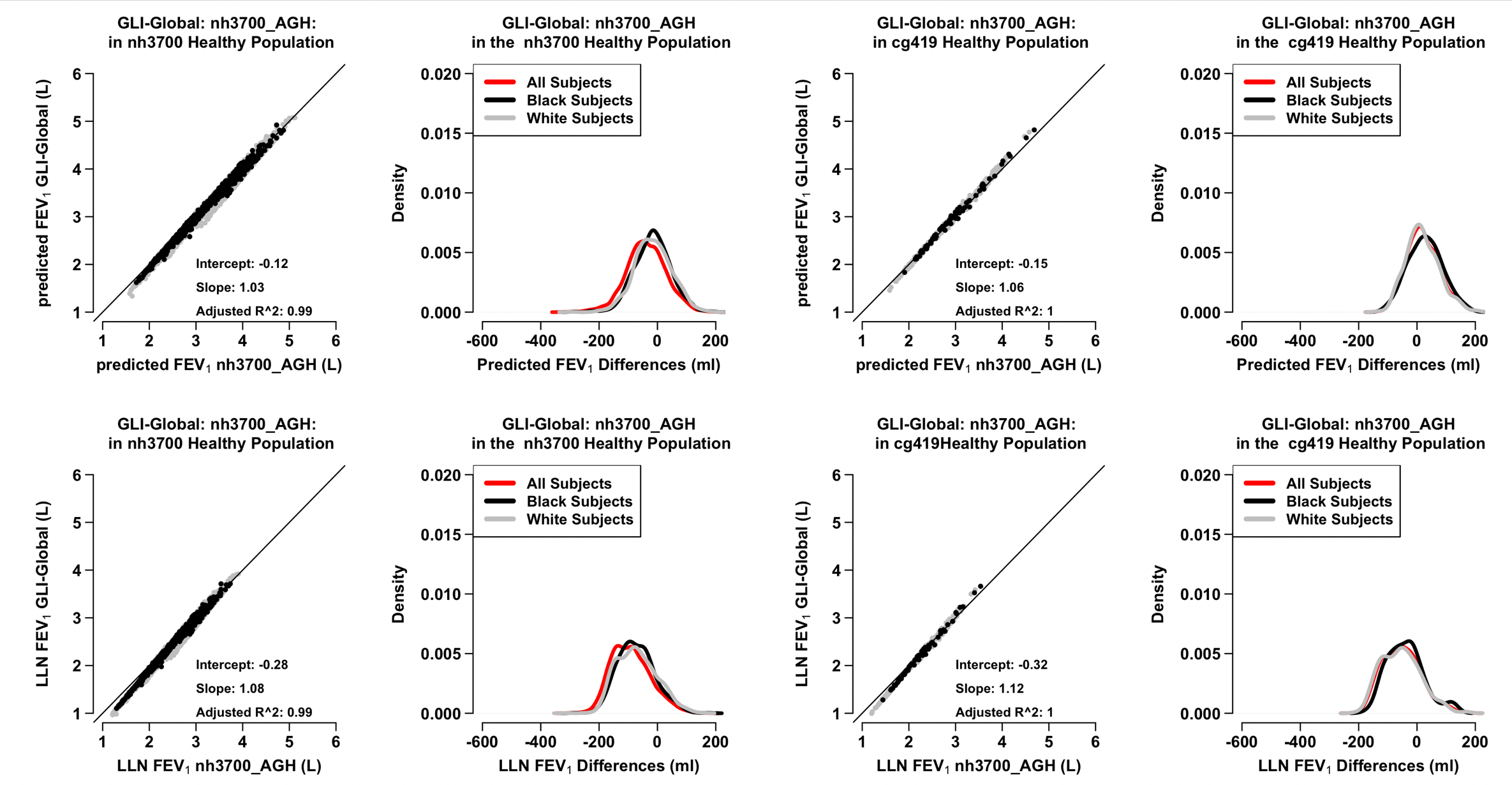


e-Figure 16. Identity and density plots of the differences of the predicted FEV_1_ (or LLN) between the race-neutral GLI-Global values and nh3700_AGH race free values in two different healthy never smoker populations. The figure shows the identity and density plots of the predicted FEV_1_ differences (top row) and the FEV_1_ LLN differences (bottom row). Density plot differences were calculated by subtracting the predicted FEV_1_ (or LLN) of the stated model from the GLI-Global value. The first two and the last two columns represent results from the nh3700 and cg419 healthy populations respectively. The black and gray points depict the Black and White populations respectively. The black, gray and red lines demonstrate the densities of the Black, White and total populations respectively. Compared to the GLI-Global the race free equations generate predicted FEV_1_ values that are higher (negative differences) and thus would generate lower ppFEV_1_ values. As expected, there were no racial differences with these race-neutral equations.

See attached excel file with list of SEQN of the nh3700 individuals included in these analyses.

References

E1. Schroeder JD, McKenzie AS, Zach JA, Wilson CG, Curran-Everett D, Stinson DS, Newell JD, Lynch DA. Relationships Between Airflow Obstruction and Quantitative CT Measurements of Emphysema, Air Trapping, and Airways in Subjects With and Without Chronic Obstructive Pulmonary Disease. *AJR Am J Roentgenol* 2013;201:W460–W470.

E2. Lowe KE, Regan EA, Anzueto A, Austin E, Austin JHM, Beaty TH, Benos PV, Benway CJ, Bhatt SP, Bleecker ER, Bodduluri S, Bon J, Boriek AM, Boueiz AR, Bowler RP, Budoff M, Casaburi R, Castaldi PJ, Charbonnier J-P, Cho MH, Comellas A, Conrad D, Costa Davis C, Criner GJ, Curran-Everett D, Curtis JL, DeMeo DL, Diaz AA, Dransfield MT, *et al.* COPDGene® 2019: Redefining the Diagnosis of Chronic Obstructive Pulmonary Disease. *Chronic Obstr Pulm Dis* 2019;6:384–399.

E3. Quanjer PH, Stanojevic S, Cole TJ, Baur X, Hall GL, Culver BH, Enright PL, Hankinson JL, Ip MSM, Zheng J, Stocks J, Initiative ERSGLF. Multi-ethnic reference values for spirometry for the 3-95-yr age range: the global lung function 2012 equations. *Eur Respir J*, 2012/06/27 ed. 2012;40:1324–1343.

E4. Hankinson JL, Odencrantz JR, Fedan KB. Spirometric reference values from a sample of the general U.S. population. *Am J Respir Crit Care Med* 1999;159:179–87.


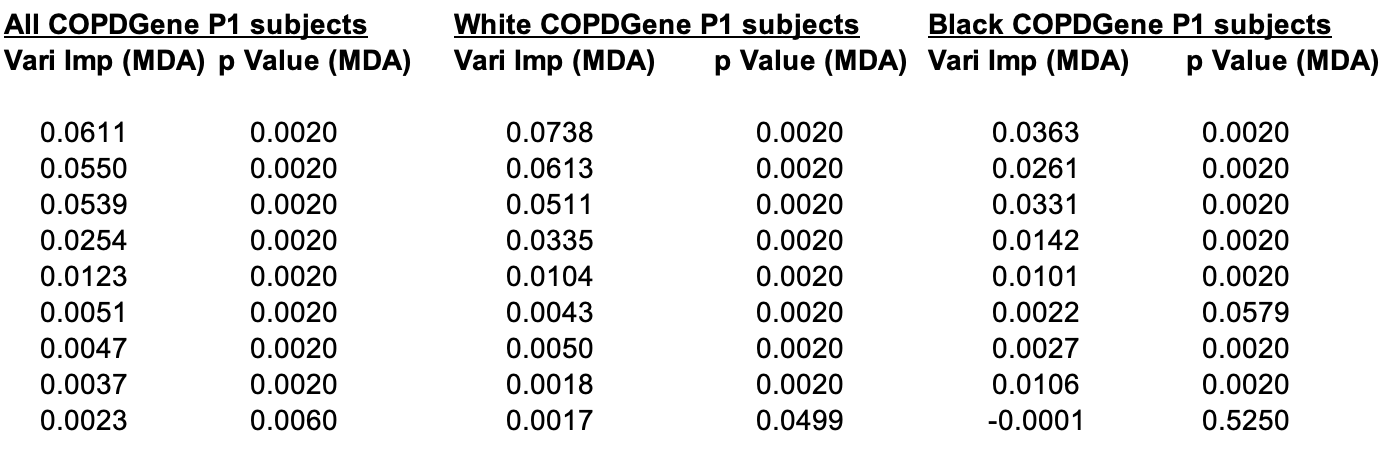


**C) Representative Random Forest Model of MMRC > 1**

**rfPermute(MMRC > 1 ~ ppFEV1 GLI+ FEV1/FVC ratio + FEF25-75 + packyears + age + bronchodilator response + 6MW distance + BMI + gender (female) , ntree=500, nrep=500)**
