# Supplementary material for: Race-Specific Spirometry Equations Do Not Improve Models of Dyspnea and Quantitative Chest CT Phenotypes
Source: Chest. 2023 Jul 26;164(6):1492–504. doi: 10.1016/j.chest.2023.07.019 (PMC10925545; doi:10.1016/j.chest.2023.07.019)
Supplement: e-Online Data [file mmc2.docx]

SEQN

41479

41483

41492

41506

41518

41522

41527

41531

41539

41542

41546

41549

41562

41563

41578

41583

41599

41605

41622

41624

41636

41639

41642

41668

41672

41677

41680

41691

41708

41718

41728

41733

41735

41740

41751

41756

41760

41761

41768

41770

41793

41796

41808

41811

41827

41839

41853

41864

41871

41873

41898

41899

41919

41923

41927

41961

41969

41981

42026

42029

42035

42047

42050

42053

42059

42064

42073

42096

42106

42120

42123

42127

42132

42133

42152

42157

42159

42160

42171

42172

42173

42189

42197

42200

42212

42239

42240

42244

42246

42251

42269

42275

42285

42291

42298

42300

42309

42321

42328

42340

42345

42346

42351

42364

42379

42382

42396

42402

42436

42437

42445

42459

42460

42471

42473

42484

42490

42491

42492

42502

42503

42523

42526

42544

42545

42559

42567

42568

42587

42591

42596

42600

42605

42612

42632

42636

42640

42642

42647

42648

42659

42665

42668

42680

42681

42689

42692

42693

42704

42712

42713

42725

42728

42736

42738

42746

42766

42772

42782

42787

42797

42802

42806

42827

42833

42834

42855

42868

42878

42890

42904

42941

42948

42949

42958

42960

42979

43004

43007

43023

43026

43030

43051

43056

43060

43062

43077

43079

43086

43087

43091

43093

43100

43106

43119

43124

43127

43134

43140

43167

43175

43180

43191

43195

43224

43227

43232

43235

43241

43244

43251

43256

43259

43296

43311

43313

43315

43329

43345

43379

43383

43399

43415

43420

43433

43453

43465

43469

43474

43478

43479

43489

43493

43496

43526

43529

43582

43588

43593

43594

43633

43635

43636

43672

43674

43678

43680

43684

43688

43691

43692

43710

43723

43727

43733

43736

43742

43758

43761

43764

43772

43775

43776

43779

43813

43824

43827

43848

43856

43865

43881

43920

43923

43924

43926

43945

43957

43960

43966

43971

43976

43991

44008

44039

44040

44041

44060

44071

44072

44086

44091

44117

44129

44158

44165

44178

44180

44190

44213

44218

44229

44232

44247

44252

44255

44257

44259

44261

44264

44267

44296

44299

44302

44304

44317

44326

44332

44351

44356

44360

44361

44363

44382

44386

44387

44391

44398

44401

44413

44415

44418

44434

44444

44451

44455

44456

44481

44514

44522

44527

44532

44550

44559

44564

44575

44576

44589

44604

44618

44633

44636

44643

44644

44647

44668

44672

44681

44683

44685

44695

44698

44706

44719

44731

44738

44758

44760

44774

44775

44778

44780

44781

44789

44791

44793

44817

44820

44848

44852

44855

44870

44877

44883

44885

44892

44898

44899

44910

44927

44930

44941

44970

44972

44973

44986

45005

45006

45013

45018

45028

45029

45031

45034

45035

45045

45051

45052

45058

45081

45087

45096

45099

45107

45113

45116

45137

45144

45171

45179

45187

45204

45207

45209

45222

45225

45229

45234

45257

45267

45271

45273

45276

45286

45287

45295

45296

45298

45308

45309

45316

45319

45338

45348

45351

45363

45368

45370

45416

45435

45437

45450

45451

45463

45483

45487

45496

45519

45536

45549

45564

45568

45573

45583

45605

45609

45616

45621

45625

45627

45631

45645

45649

45657

45674

45717

45734

45744

45763

45777

45782

45784

45811

45826

45828

45835

45836

45838

45848

45850

45851

45878

45903

45905

45927

45949

45951

45952

45957

45966

45970

45974

46029

46035

46038

46040

46061

46074

46085

46102

46103

46111

46141

46154

46159

46188

46193

46194

46195

46198

46202

46218

46232

46249

46261

46269

46275

46285

46288

46316

46320

46334

46337

46345

46349

46354

46357

46358

46368

46384

46385

46386

46400

46412

46419

46425

46433

46436

46452

46455

46457

46468

46481

46485

46493

46496

46504

46509

46512

46536

46537

46554

46565

46571

46574

46578

46580

46586

46593

46594

46597

46598

46609

46620

46660

46666

46674

46682

46684

46691

46704

46710

46731

46733

46736

46740

46741

46746

46754

46761

46778

46780

46781

46784

46794

46800

46820

46823

46836

46846

46847

46854

46864

46872

46883

46905

46918

46925

46936

46940

46955

46957

46972

46983

46984

46990

46997

47063

47068

47083

47096

47116

47136

47141

47146

47151

47162

47172

47173

47179

47191

47203

47216

47222

47223

47226

47228

47232

47244

47257

47274

47302

47303

47318

47319

47324

47327

47350

47352

47369

47395

47396

47403

47404

47410

47419

47425

47426

47430

47437

47444

47447

47460

47475

47497

47513

47518

47550

47552

47558

47563

47571

47579

47610

47619

47623

47624

47636

47661

47668

47671

47704

47706

47709

47716

47730

47754

47799

47802

47808

47809

47819

47825

47826

47831

47842

47872

47877

47880

47899

47903

47904

47911

47936

47940

47950

47958

47982

47987

48003

48040

48051

48054

48063

48070

48077

48081

48089

48092

48103

48140

48155

48156

48168

48181

48194

48211

48229

48250

48253

48256

48264

48266

48276

48278

48316

48325

48327

48335

48346

48348

48353

48360

48361

48370

48377

48382

48396

48401

48403

48413

48415

48422

48429

48438

48446

48460

48468

48491

48497

48500

48501

48513

48515

48522

48524

48538

48541

48552

48554

48569

48588

48589

48596

48597

48599

48611

48640

48670

48695

48701

48704

48705

48717

48719

48757

48788

48790

48796

48801

48803

48805

48817

48818

48865

48884

48887

48904

48905

48907

48922

48951

48960

48965

48971

48974

49000

49021

49026

49034

49035

49046

49049

49055

49061

49066

49068

49078

49081

49083

49103

49114

49118

49121

49123

49149

49157

49158

49166

49184

49194

49220

49234

49250

49271

49280

49291

49295

49307

49314

49316

49331

49334

49335

49348

49349

49363

49366

49395

49401

49406

49416

49445

49452

49458

49477

49489

49492

49501

49510

49516

49526

49529

49540

49563

49565

49575

49576

49584

49586

49599

49601

49612

49616

49622

49638

49641

49656

49664

49665

49667

49673

49680

49684

49707

49731

49736

49741

49742

49743

49748

49749

49751

49758

49759

49762

49770

49773

49782

49805

49815

49818

49832

49849

49866

49868

49884

49887

49890

49893

49901

49905

49907

49916

49917

49924

49925

49944

49945

49947

49967

49970

49981

49986

49995

49996

50001

50002

50026

50029

50037

50040

50044

50052

50054

50057

50074

50085

50094

50119

50129

50131

50133

50137

50142

50144

50146

50157

50173

50182

50185

50190

50194

50202

50205

50208

50211

50225

50228

50229

50236

50239

50247

50274

50277

50279

50302

50319

50321

50335

50347

50355

50357

50358

50385

50396

50406

50439

50450

50463

50464

50478

50487

50498

50509

50522

50523

50534

50543

50544

50545

50548

50556

50561

50567

50582

50590

50591

50595

50609

50615

50624

50625

50629

50635

50636

50649

50652

50656

50659

50665

50678

50679

50709

50714

50718

50750

50755

50770

50779

50780

50786

50787

50810

50814

50820

50835

50837

50842

50850

50855

50872

50879

50884

50894

50902

50905

50910

50915

50926

50927

50932

50936

50940

50941

50949

50967

50975

50985

50990

50994

50997

50998

51002

51003

51006

51009

51023

51024

51064

51089

51110

51111

51123

51147

51148

51155

51156

51165

51168

51183

51185

51200

51203

51204

51217

51226

51227

51231

51242

51264

51270

51286

51287

51290

51295

51304

51307

51321

51328

51329

51336

51338

51354

51356

51392

51399

51413

51415

51426

51430

51473

51482

51484

51497

51500

51503

51510

51541

51554

51564

51617

51622

51643

51645

51647

51653

51655

51656

51657

51661

51667

51690

51692

51694

51696

51701

51707

51709

51718

51721

51728

51735

51738

51744

51750

51754

51768

51779

51780

51796

51799

51805

51807

51808

51815

51818

51830

51831

51839

51848

51849

51865

51866

51867

51875

51877

51880

51892

51906

51911

51916

51936

51942

51944

51951

51952

51957

51963

51964

51967

51972

51973

51991

51993

51997

52009

52013

52023

52029

52032

52039

52063

52069

52084

52085

52086

52090

52099

52104

52112

52115

52131

52138

52148

52173

52175

52179

52182

52193

52202

52203

52212

52215

52216

52217

52222

52227

52231

52245

52251

52276

52282

52283

52295

52303

52310

52332

52333

52355

52361

52372

52376

52381

52392

52430

52431

52437

52445

52457

52458

52462

52467

52471

52473

52503

52510

52516

52535

52537

52541

52543

52549

52553

52557

52560

52565

52572

52581

52583

52584

52586

52591

52602

52607

52611

52621

52627

52629

52642

52643

52646

52648

52650

52652

52655

52656

52678

52686

52689

52693

52702

52717

52718

52727

52728

52731

52750

52761

52766

52795

52801

52814

52837

52838

52845

52846

52849

52853

52869

52874

52894

52902

52924

52927

52960

52979

52989

53003

53012

53019

53038

53040

53042

53044

53051

53075

53085

53096

53102

53110

53112

53117

53121

53125

53127

53130

53151

53161

53171

53175

53177

53181

53191

53193

53199

53211

53217

53220

53221

53222

53228

53234

53238

53247

53266

53268

53272

53278

53286

53291

53302

53306

53317

53341

53359

53366

53375

53381

53383

53395

53404

53420

53434

53439

53461

53467

53471

53492

53496

53501

53508

53528

53530

53534

53537

53544

53548

53550

53555

53557

53558

53565

53582

53592

53597

53604

53610

53617

53618

53625

53643

53670

53672

53678

53681

53682

53684

53686

53695

53696

53697

53706

53719

53723

53732

53743

53745

53746

53750

53762

53772

53774

53777

53785

53790

53791

53807

53831

53833

53875

53876

53877

53884

53900

53913

53918

53952

53958

53972

53987

53991

53998

54008

54027

54054

54062

54066

54077

54078

54085

54088

54091

54094

54095

54096

54119

54130

54159

54162

54166

54175

54192

54205

54219

54222

54234

54236

54238

54242

54245

54251

54253

54255

54291

54292

54295

54301

54302

54303

54304

54306

54312

54325

54326

54346

54356

54357

54363

54367

54391

54399

54401

54419

54430

54439

54440

54450

54451

54464

54466

54472

54476

54487

54491

54493

54524

54532

54533

54542

54558

54560

54565

54575

54577

54598

54612

54618

54631

54632

54646

54659

54679

54711

54719

54731

54734

54737

54746

54750

54758

54770

54773

54783

54784

54790

54802

54803

54808

54814

54825

54826

54829

54838

54846

54870

54873

54901

54902

54919

54920

54925

54927

54936

54948

54957

54959

54963

54966

54968

54984

54994

55004

55006

55007

55012

55014

55017

55020

55021

55026

55034

55036

55053

55059

55072

55075

55079

55089

55099

55108

55114

55115

55120

55127

55138

55143

55150

55158

55165

55170

55173

55190

55191

55194

55198

55202

55208

55211

55212

55234

55241

55246

55254

55258

55270

55272

55276

55289

55292

55295

55297

55303

55306

55310

55312

55313

55317

55319

55326

55328

55337

55340

55353

55368

55382

55384

55397

55405

55411

55413

55426

55435

55436

55441

55446

55447

55453

55459

55471

55473

55476

55483

55484

55493

55497

55505

55511

55529

55536

55559

55560

55562

55576

55580

55605

55607

55608

55620

55633

55636

55638

55646

55647

55667

55678

55685

55687

55697

55701

55721

55730

55734

55737

55740

55760

55763

55774

55783

55784

55797

55802

55808

55830

55839

55847

55848

55862

55864

55868

55881

55883

55884

55886

55891

55892

55902

55904

55913

55916

55924

55927

55932

55933

55936

55948

55953

55965

55966

55967

55973

55985

55996

56006

56032

56039

56066

56088

56096

56106

56107

56109

56113

56120

56123

56129

56131

56140

56147

56149

56159

56163

56173

56176

56179

56187

56191

56198

56201

56222

56233

56234

56240

56242

56267

56283

56294

56297

56299

56308

56314

56323

56328

56336

56337

56352

56357

56368

56371

56396

56402

56403

56408

56415

56420

56423

56430

56432

56435

56436

56439

56470

56481

56489

56505

56508

56511

56519

56527

56529

56539

56545

56546

56554

56581

56596

56610

56619

56628

56634

56641

56646

56650

56651

56658

56660

56686

56691

56703

56720

56727

56739

56755

56763

56764

56785

56792

56793

56798

56805

56809

56829

56830

56834

56850

56854

56855

56870

56881

56885

56890

56913

56941

56949

56967

56971

56973

56976

56980

56983

56984

57000

57009

57021

57028

57053

57086

57089

57095

57108

57109

57119

57129

57142

57145

57159

57175

57177

57178

57182

57185

57187

57193

57204

57211

57215

57229

57234

57247

57262

57272

57276

57280

57291

57293

57295

57301

57305

57327

57335

57343

57348

57367

57373

57375

57377

57388

57390

57396

57403

57408

57417

57420

57439

57457

57471

57475

57480

57502

57510

57512

57521

57524

57532

57549

57550

57553

57561

57563

57569

57577

57579

57587

57589

57601

57606

57612

57624

57626

57641

57661

57669

57674

57681

57685

57687

57693

57698

57702

57704

57710

57715

57720

57722

57746

57748

57751

57754

57785

57787

57788

57791

57797

57808

57814

57815

57818

57821

57822

57829

57831

57832

57833

57838

57844

57847

57858

57859

57873

57890

57893

57895

57907

57914

57920

57925

57930

57932

57933

57936

57939

57945

57948

57966

57985

57986

57997

58000

58009

58027

58028

58032

58033

58042

58060

58067

58069

58083

58088

58094

58108

58115

58134

58164

58168

58185

58190

58202

58219

58221

58225

58234

58236

58238

58242

58251

58257

58265

58269

58272

58274

58282

58284

58286

58290

58302

58319

58323

58329

58331

58332

58339

58348

58377

58384

58390

58398

58408

58415

58416

58417

58418

58421

58429

58433

58435

58450

58454

58458

58460

58471

58492

58495

58497

58504

58505

58507

58519

58520

58522

58525

58530

58532

58555

58559

58560

58561

58563

58564

58572

58573

58583

58586

58591

58633

58649

58670

58671

58673

58681

58684

58689

58693

58710

58738

58775

58779

58797

58798

58801

58806

58819

58822

58852

58853

58855

58860

58861

58864

58876

58884

58905

58916

58926

58928

58929

58933

58947

58961

58971

58975

58979

58980

58981

58989

59000

59006

59009

59018

59019

59028

59030

59035

59038

59046

59052

59053

59075

59076

59079

59113

59137

59140

59146

59148

59149

59156

59165

59174

59179

59185

59196

59205

59213

59214

59223

59224

59236

59247

59249

59253

59256

59271

59294

59295

59306

59314

59326

59333

59340

59342

59349

59370

59379

59384

59385

59390

59391

59397

59405

59418

59426

59432

59437

59439

59440

59446

59447

59461

59462

59468

59480

59484

59486

59497

59498

59502

59509

59515

59521

59524

59528

59530

59536

59537

59546

59556

59558

59560

59562

59571

59600

59603

59608

59610

59616

59618

59627

59631

59636

59665

59667

59674

59678

59689

59693

59707

59721

59742

59749

59754

59755

59758

59765

59780

59800

59803

59804

59806

59808

59810

59818

59836

59838

59844

59852

59865

59867

59882

59888

59909

59910

59916

59921

59940

59953

59957

59972

59992

60004

60024

60030

60039

60043

60044

60062

60066

60069

60086

60094

60101

60102

60104

60106

60109

60110

60111

60112

60116

60133

60152

60160

60161

60162

60170

60175

60195

60198

60216

60218

60231

60235

60242

60243

60247

60250

60269

60273

60296

60301

60306

60308

60318

60321

60328

60334

60347

60348

60377

60387

60388

60390

60397

60406

60421

60439

60440

60454

60456

60465

60469

60490

60506

60519

60533

60551

60559

60562

60567

60583

60584

60586

60605

60629

60639

60640

60655

60663

60664

60677

60690

60693

60706

60709

60718

60722

60735

60743

60749

60756

60763

60782

60791

60810

60829

60842

60845

60847

60850

60867

60871

60873

60881

60883

60893

60895

60905

60906

60916

60922

60923

60957

60969

60973

60977

60979

60989

60991

60994

61029

61036

61041

61044

61051

61062

61063

61073

61085

61091

61102

61103

61110

61111

61119

61134

61141

61163

61165

61166

61167

61169

61182

61189

61192

61196

61200

61202

61203

61217

61219

61221

61222

61229

61234

61248

61257

61258

61265

61270

61300

61303

61333

61340

61343

61353

61360

61380

61382

61415

61417

61422

61429

61430

61439

61447

61451

61480

61488

61496

61501

61510

61512

61523

61528

61534

61542

61550

61571

61572

61576

61577

61578

61580

61590

61595

61602

61617

61625

61630

61635

61649

61654

61657

61659

61663

61664

61672

61676

61679

61696

61699

61704

61713

61745

61755

61770

61775

61778

61780

61789

61790

61791

61792

61828

61830

61834

61858

61880

61885

61886

61890

61893

61899

61908

61917

61924

61938

61943

61948

61956

61958

61960

61965

61967

61968

61974

61979

61989

62013

62030

62040

62045

62050

62058

62063

62064

62065

62067

62080

62083

62097

62102

62103

62105

62108

62112

62125

62142

62152

62158

62160

62164

62179

62200

62202

62208

62209

62215

62217

62231

62236

62275

62284

62287

62302

62303

62308

62309

62320

62326

62333

62340

62342

62367

62372

62422

62429

62432

62434

62437

62449

62452

62454

62465

62481

62500

62506

62509

62512

62515

62536

62548

62549

62551

62589

62599

62616

62621

62625

62650

62671

62683

62706

62714

62719

62720

62724

62735

62744

62746

62747

62750

62752

62761

62768

62788

62792

62794

62805

62808

62818

62824

62841

62847

62848

62855

62860

62876

62880

62882

62885

62900

62901

62912

62937

62953

62971

62994

63002

63006

63008

63021

63025

63031

63033

63044

63045

63070

63071

63077

63089

63107

63110

63112

63123

63125

63126

63137

63148

63149

63154

63162

63164

63167

63174

63181

63194

63196

63213

63216

63221

63222

63225

63254

63261

63262

63263

63276

63283

63288

63291

63293

63296

63297

63313

63323

63328

63341

63345

63346

63349

63363

63365

63377

63382

63385

63388

63405

63412

63413

63422

63439

63449

63471

63474

63485

63491

63506

63510

63515

63524

63534

63540

63541

63543

63551

63554

63559

63560

63571

63579

63587

63607

63608

63621

63626

63629

63651

63663

63665

63684

63697

63699

63700

63701

63710

63713

63714

63723

63731

63749

63754

63759

63771

63777

63780

63781

63802

63813

63817

63828

63829

63830

63834

63838

63847

63850

63853

63860

63861

63868

63871

63875

63882

63889

63948

63949

63955

63963

63968

63971

63974

63992

63995

63998

64021

64032

64034

64042

64055

64058

64061

64064

64065

64071

64072

64077

64086

64091

64095

64098

64099

64131

64132

64134

64138

64142

64163

64173

64179

64186

64213

64217

64218

64222

64235

64256

64261

64263

64265

64281

64290

64305

64310

64315

64325

64330

64333

64360

64367

64396

64397

64402

64411

64427

64438

64439

64440

64471

64474

64477

64494

64495

64498

64515

64526

64532

64536

64544

64550

64552

64584

64585

64593

64599

64610

64611

64625

64628

64643

64648

64664

64669

64674

64675

64676

64680

64689

64705

64719

64725

64742

64756

64777

64785

64792

64800

64802

64807

64808

64821

64823

64828

64839

64873

64876

64877

64897

64938

64942

64948

64953

64961

64963

64973

64974

64975

64976

64978

64981

64992

64995

65006

65014

65040

65046

65047

65052

65061

65069

65075

65086

65095

65102

65112

65125

65126

65129

65135

65144

65173

65176

65184

65192

65201

65209

65214

65228

65230

65255

65256

65260

65261

65262

65265

65267

65298

65304

65327

65331

65337

65346

65348

65353

65375

65391

65393

65407

65410

65412

65427

65443

65447

65450

65453

65454

65458

65473

65474

65475

65477

65485

65492

65497

65503

65506

65512

65518

65520

65564

65571

65577

65585

65586

65587

65593

65603

65606

65613

65617

65629

65632

65634

65650

65658

65659

65676

65680

65682

65699

65702

65705

65730

65735

65743

65758

65760

65768

65781

65782

65787

65797

65807

65808

65827

65846

65854

65857

65867

65868

65874

65893

65898

65909

65910

65921

65927

65929

65944

65951

65963

65986

65992

65994

66010

66015

66026

66032

66041

66043

66053

66070

66093

66095

66108

66121

66124

66126

66127

66138

66141

66160

66162

66177

66178

66189

66197

66201

66206

66215

66217

66222

66240

66243

66254

66256

66264

66268

66278

66280

66298

66306

66317

66327

66334

66341

66362

66378

66384

66386

66397

66404

66410

66412

66416

66438

66442

66456

66460

66462

66465

66475

66511

66515

66520

66525

66527

66536

66544

66547

66550

66554

66556

66561

66563

66564

66575

66582

66587

66598

66614

66619

66620

66628

66630

66633

66654

66671

66672

66680

66686

66708

66720

66725

66728

66731

66736

66754

66759

66789

66808

66838

66848

66850

66869

66873

66881

66883

66889

66896

66898

66901

66903

66907

66914

66924

66938

66947

66949

66958

66959

66961

66988

66994

67000

67007

67034

67037

67051

67056

67070

67074

67098

67113

67148

67171

67175

67181

67182

67183

67185

67189

67193

67198

67201

67223

67226

67236

67249

67250

67255

67259

67269

67283

67285

67293

67306

67327

67332

67333

67337

67340

67341

67349

67352

67356

67365

67373

67375

67376

67386

67388

67395

67406

67408

67411

67417

67425

67434

67439

67442

67445

67449

67452

67466

67470

67474

67477

67480

67481

67483

67486

67487

67502

67505

67515

67516

67530

67534

67564

67571

67584

67585

67592

67596

67597

67607

67620

67631

67632

67646

67647

67649

67666

67670

67677

67710

67716

67717

67726

67740

67743

67763

67766

67767

67773

67779

67781

67782

67787

67788

67793

67797

67820

67826

67827

67841

67865

67872

67873

67877

67887

67897

67898

67901

67907

67916

67924

67926

67951

67960

67969

67975

67986

67992

68027

68034

68041

68061

68069

68077

68078

68080

68089

68091

68093

68095

68099

68102

68112

68137

68139

68142

68145

68147

68152

68154

68155

68156

68170

68197

68222

68226

68236

68252

68254

68255

68263

68270

68271

68275

68277

68283

68285

68293

68310

68312

68319

68338

68341

68342

68353

68364

68374

68380

68387

68394

68403

68404

68415

68418

68423

68424

68434

68438

68441

68442

68452

68453

68467

68474

68478

68507

68512

68531

68544

68548

68556

68564

68565

68575

68580

68582

68589

68591

68595

68602

68614

68632

68633

68635

68645

68646

68661

68662

68663

68667

68669

68672

68678

68687

68688

68689

68710

68722

68726

68753

68767

68769

68790

68791

68802

68818

68823

68837

68843

68846

68859

68871

68877

68884

68886

68901

68910

68914

68916

68920

68934

68946

68972

68976

68978

68979

68998

69020

69022

69025

69027

69045

69047

69057

69062

69068

69077

69085

69105

69110

69120

69137

69146

69147

69170

69171

69172

69203

69212

69213

69221

69227

69229

69237

69243

69248

69253

69258

69283

69288

69299

69301

69320

69328

69335

69356

69365

69366

69368

69391

69392

69400

69402

69404

69410

69419

69422

69425

69434

69450

69452

69453

69456

69458

69462

69465

69472

69479

69488

69489

69491

69494

69499

69502

69511

69524

69536

69537

69553

69556

69569

69618

69621

69626

69632

69633

69644

69652

69661

69666

69667

69672

69688

69689

69701

69702

69716

69738

69746

69755

69767

69778

69782

69787

69789

69803

69806

69815

69822

69823

69825

69833

69842

69843

69855

69856

69858

69861

69869

69870

69922

69923

69936

69941

69943

69959

69962

69979

69991

69996

69997

70008

70033

70046

70054

70055

70062

70063

70069

70077

70094

70095

70101

70115

70117

70120

70123

70130

70131

70136

70143

70150

70158

70163

70164

70175

70183

70186

70196

70200

70213

70217

70219

70223

70226

70228

70235

70246

70247

70248

70251

70254

70255

70265

70268

70293

70294

70295

70300

70313

70338

70341

70342

70355

70367

70368

70389

70405

70423

70426

70427

70438

70442

70443

70446

70457

70464

70473

70480

70489

70498

70499

70502

70505

70511

70521

70527

70531

70533

70534

70554

70561

70562

70574

70575

70581

70584

70607

70613

70635

70644

70649

70653

70670

70681

70686

70710

70721

70725

70727

70728

70730

70757

70759

70766

70774

70783

70784

70785

70786

70790

70794

70804

70805

70825

70865

70869

70877

70894

70897

70906

70920

70923

70932

70981

70982

70983

70984

70996

71003

71008

71011

71021

71037

71061

71066

71068

71071

71090

71091

71092

71105

71120

71123

71133

71135

71143

71159

71164

71169

71171

71172

71182

71200

71203

71212

71213

71215

71219

71224

71228

71231

71232

71266

71291

71292

71306

71310

71312

71314

71321

71328

71341

71351

71361

71372

71381

71407

71416

71417

71419

71420

71422

71428

71430

71436

71449

71452

71455

71468

71473

71482

71509

71525

71526

71531

71536

71540

71544

71555

71559

71577

71580

71581

71584

71591

71595

71604

71605

71615

71625

71626

71635

71639

71640

71655

71657

71660

71678

71702

71712

71715

71718

71730

71737

71741

71744

71748

71752

71765

71773

71786

71793

71803

71811

71813

71820

71824

71828

71841

71846

71849

71857

71863

71875

71880

71884

71885

71886

71891

71897

71898

71908

71915
