# Supplementary material for: Race-Specific Spirometry Equations Do Not Improve Models of Dyspnea and Quantitative Chest CT Phenotypes
Source: Chest. 2023 Jul 26;164(6):1492–504. doi: 10.1016/j.chest.2023.07.019 (PMC10925545; doi:10.1016/j.chest.2023.07.019)
Supplement: e-Online Data [file mmc3.docx]

SEQN

41482

41501

41514

41525

41528

41575

41589

41613

41699

41729

41791

41797

41865

41881

41897

41904

41952

41964

42107

42135

42185

42193

42211

42255

42260

42307

42310

42338

42424

42524

42546

42581

42594

42626

42649

42683

42696

42751

42820

42835

42906

42922

42927

42947

42985

43073

43078

43085

43168

43186

43237

43271

43275

43284

43330

43342

43391

43411

43418

43441

43476

43492

43500

43558

43562

43573

43575

43592

43595

43614

43639

43718

43756

43839

43859

43895

43906

43964

44012

44021

44027

44085

44176

44184

44189

44201

44206

44215

44223

44233

44260

44276

44333

44380

44385

44457

44465

44531

44545

44595

44600

44680

44844

44862

44882

44906

44928

44951

45056

45103

45118

45169

45174

45186

45233

45237

45243

45247

45277

45278

45282

45284

45299

45302

45318

45366

45401

45422

45444

45462

45588

45593

45640

45660

45754

45766

45787

45813

45882

45894

45918

45968

45992

45997

46050

46075

46101

46140

46158

46172

46176

46190

46281

46293

46346

46348

46360

46364

46406

46428

46484

46486

46522

46523

46531

46606

46657

46776

46828

46835

46853

46857

46897

46902

46927

47044

47073

47084

47154

47167

47176

47397

47418

47455

47469

47485

47505

47533

47588

47631

47640

47701

47746

47759

47788

47790

47813

47868

47869

47937

47943

47977

48002

48006

48018

48021

48068

48069

48164

48176

48182

48215

48247

48284

48289

48297

48298

48314

48433

48452

48471

48495

48561

48666

48671

48716

48744

48786

48827

48853

48859

48886

48924

48932

48948

49001

49040

49052

49071

49106

49116

49150

49196

49247

49251

49256

49281

49346

49405

49454

49567

49585

49590

49593

49609

49618

49635

49704

49719

49728

49745

49828

49879

49880

49906

49957

50041

50101

50102

50109

50116

50121

50135

50165

50221

50333

50334

50354

50383

50419

50423

50431

50484

50507

50517

50526

50587

50608

50647

50658

50689

50691

50788

50806

50823

50864

50875

50935

50943

50999

51008

51012

51054

51073

51077

51127

51131

51140

51177

51201

51267

51280

51282

51351

51365

51493

51518

51569

51587

51590

51606

51620

51678

51714

51821

51844

51919

52031

52042

52065

52068

52081

52088

52126

52129

52135

52184

52207

52226

52229

52273

52307

52366

52446

52497

52509

52545

52566

52576

52577

52632

52661

52723

52759

52813

52881

52942

53035

53068

53092

53124

53184

53188

53242

53269

53356

53436

53443

53526

53532

53546

53572

53595

53889

53895

53942

54010

54051

54104

54142

54170

54200

54254

54267

54270

54416

54482

54504

54509

54516

54569

54589

54629

54634

54636

54642

54698

54709

54738

54760

54812

54822

54861

54875

54945

55019

55174

55177

55256

55294

55342

55423

55427

55466

55527

55533

55592

55664

55736

55750

55814

55831

55849

55871

55877

55974

56041

56219

56220

56258

56266

56275

56305

56307

56382

56455

56466

56567

56573

56594

56632

56710

56716

56780

56784

56800

56827

56839

56851

56952

56969

56985

56989

57024

57031

57050

57122

57136

57137

57164

57179

57223

57249

57302

57358

57441

57482

57554

57570

57753

57800

57848

57884

57916

57940

57977

58024

58192

58215

58283

58296

58326

58380

58407

58571

58590

58624

58640

58642

58650

58656

58704

58714

58718

58764

58826

58857

58877

58887

58898

58974

59059

59125

59206

59230

59232

59296

59320

59321

59324

59334

59412

59495

59541

59580

59612

59692

59733

59802

59931

59982

60057

60058

60073

60131

60135

60146

60155

60183

60184

60229

60274

60279

60299

60332

60345

60374

60457

60475

60525

60535

60577

60628

60760

60772

60827

60833

60896

60908

60914

60918

60940

60995

61006

61107

61108

61126

61155

61207

61227

61253

61261

61329

61362

61366

61377

61414

61573

61642

61686

61717

61720

61727

61777

61839

61874

61875

61986

62019

62041

62076

62126

62148

62230

62233

62237

62259

62289

62317

62413

62431

62441

62474

62484

62634

62644

62651

62664

62793

62814

62821

62835

62859

62924

62982

63091

63106

63109

63159

63215

63248

63329

63465

63553

63569

63593

63687

63689

63707

63804

63806

63839

63887

63902

63916

63964

63965

64036

64048

64063

64082

64164

64181

64328

64347

64530

64582

64651

64867

64895

64930

64998

65032

65139

65143

65165

65183

65266

65285

65297

65431

65633

65660

65824

65837

65877

65885

65993

66068

66071

66077

66140

66276

66297

66441

66451

66504

66643

66695

66718

66781

66796

66804

66863

66944

67001

67092

67119

67141

67149

67194

67243

67300

67361

67399

67414

67447

67457

67493

67589

67614

67643

67658

67703

67714

67848

67861

67863

67939

67947

68035

68075

68181

68191

68196

68237

68365

68416

68482

68561

68573

68627

68740

68787

68799

68816

68854

68888

68947

68955

68966

69142

69151

69166

69256

69270

69284

69287

69403

69431

69473

69486

69509

69523

69547

69550

69624

69658

69668

69669

69684

69773

69777

69832

69866

69868

69892

69898

69983

70035

70102

70134

70152

70362

70386

70495

70565

70590

70605

70675

70746

70793

70821

70871

70911

70949

70974

70993

71040

71051

71227

71315

71346

71401

71534

71569

71578

71662

71671

71682

71704

71775

71796

71836

71901
